# Flow-over-volume elevation (FOVE) on Spirometry Allows for Early Recognition of Usual Interstitial Pneumonia

**DOI:** 10.2174/0118743064498371260922043417

**Published:** 2026-09-24

**Authors:** Douglas C. Johnson

**Affiliations:** 1 Department of Medicine, Baystate Medical Center, UMass Chan Medical School, Springfield, United States of America

**Keywords:** DLCO, Lung volume, Maximal voluntary ventilation, Race-neutral reference equations, Smoking, Specific conductance, Spirometry, Usual interstitial pneumonia

## Abstract

**Introduction:**

While most patients with usual interstitial pneumonia (UIP) have reduced DLCO at the time of diagnosis, many have normal FEV1, FVC, and FEV1/FVC. Other PFT parameters representing increased lung stiffness and elastic recoil may allow earlier identification of UIP.

**Methods:**

This retrospective cohort study evaluated the relationships among spirometry, including novel parameters, lung volume, and DLCO in patients with UIP. Findings were evaluated by first *versus* last study, smoking history, and whether “normal spirometry” by ATS/ERS criteria.

**Results:**

At first testing, 56.7% of 194 UIP patients had FOVE (flow-over-volume elevation) with PEF/FVC, FEF25-75/FVC, or FEF75/FVC above the ULN, and 68.6% were near FOVE. 53.1% had “normal spirometry,” and of those, 47.6% were FOVE and 63.1% near FOVE. At first DLCO testing, 21.2% had normal spirometry, lung volume, and KCO, and of those, 48.3% were FOVE, 58.6% near FOVE, and 37.9% low DACO (predicted DLCO adjusted for lung volume). Heavy smokers had higher FVC (*p*<.001) and TLC (*p*=.004) and lower flows/FVC, DLCO, DACO, and DLCO and DACO relative to FVC or TLC (*p*<.001). There were significant changes from first to final testing. DACO better measured diffusion than KCO.

**Discussion:**

Existing standards for PFT interpretation do not consider novel spirometry parameters. This study shows that patients with UIP often have FOVE with elevated flows/FVC. Recommendations are provided for race-neutral PFT reference equations and for improving PFT reporting and interpretation.

**Conclusion:**

FOVE is common in UIP patients with otherwise normal spirometry. PFT reporting and interpretation should include PEF/FVC and DACO. Identifying FOVE improves spirometry interpretation and may lead to earlier identification of UIP.

## 1 INTRODUCTION

Usual Interstitial pneumonia (UIP) is a progressive fibrosing interstitial lung disease (ILD) associated with a high mortality rate [1]. With pirfenidone and nintedanib [2, 3] available since 2014 for the treatment of UIP, earlier identification is important.

PFTs are recommended in the evaluation of patients with suspected interstitial lung disease and to assess the degree of impairment [4]. Patients with UIP often present with symptoms of cough and dyspnea on exertion, lower crackles on chest exam, or incidental findings of interstitial abnormalities on chest CT. Evaluation often starts with spirometry, and if abnormal, further testing includes lung volumes, DLCO, and chest CT.

ATS/ERS standards for interpretation of PFTs emphasize reductions in FEV1, FVC, and FEV1/FVC for spirometry, TLC for lung volumes, and DLCO not adjusted for lung volume [5], with PFTs having FEV1, FVC, and FEV1/FVC above the LLN considered “normal spirometry” even with abnormal findings such as increased flows relative to volume that are consistent with lung stiffness and elastic recoil. With reference equations now available, novel parameters such as PEF/FVC relating flows to volumes [6] can be evaluated.

DACO (adjusting predicted DLCO for lung volume) can help determine the contribution of low lung volume and lung parenchymal disease to low DLCO [7, 8].

This study evaluates spirometry, including novel parameters, maximal voluntary ventilation (MVV), TLC, sGaw, DLCO, KCO, and DACO among patients with UIP to evaluate for early findings. Comparisons of >20 pack-year *versus* <10 pack-year smoking, of first *versus* last study, and of those with “normal” *versus* abnormal spirometry were performed. Spirometry reference equations [6] were adjusted to be race-neutral and compared with the Global Lung Initiative (GLI).

## 2 STUDY DESIGN AND METHODS

To identify patients with UIP, Baystate Health system’s electronic medical record (EMR) was searched for patients prescribed nintedanib or pirfenidone from 2014 through April 2024. All PFTs through June 2024 were included except those without subsequent PFTs for 5 years, having DLCO without spirometry, or performed post-lung transplant. Exclusion criteria included not having health system PFTs, PFTs only after lung transplant, or not having UIP based on chest CT or lung biopsy. Ethnicity, race, smoking history, and clinical data were obtained at the time of PFT and from the EMR. This study was approved by Baystate Health IRB #2087280-2 with exemption from obtaining informed consent.

Clinical data were entered into REDCap. PFT results were exported from ComPAS to REDCap. Data included birth date and sex, ethnicity, smoking history, height, weight, hemoglobin, PFT values, and race-neutral predicted values. REDCap data were exported to Excel, and further analysis included counts, frequency graphs, mean, and standard deviation. Two-tailed Student t-test and Fisher’s exact test were used to compare subgroups of first *vs* last study and of <10 pack-year *vs* >20 pack-year smoking.

PFTs were analyzed by all studies, first and last PFT with spirometry, first and last PFT with DLCO, <10 pack-year (which includes never-smokers), and >20 pack-year smoking. PFTs were analyzed in patients with “normal spirometry” and those with PRISm (preserved ratio impaired spirometry) having FEV1 < 80% predicted and FEV1/FVC ≥ 70% [9]. Comparisons were made between spirometry, lung volume, DLCO, MVV/FEV1, and (VA+estimated dead space)/TLC.

PFTs were performed as outpatients using ComPAS (Morgan Scientific), with studies available since 2005. ATS/ERS standards were followed for spirometry [10, 11], lung volumes [12], DLCO [13] and MVV [10]. Spirometry included FEV05, FEV1, FEV3, FEV6, FEVPEF, FVC, FEF25-75, FEF50, FEF75, FEFPEF, PEF, PIF, and FET from which ratios FEV05, FEV1, FEV3, FEV6, FEVPEF over FVC, FEV1/FEV3, PEF/FEV1, ratios PEF, FEF25-75, FEF50, FEF75 over FVC and ratios FEF25-75, FEF50, FEF75, PIF over PEF were calculated. Flow/volume parameters are expressed as (liter/second)/liter and flow/PEF parameters as a percentage. Central and peripheral concavity scores were calculated [14]. TLC was determined by plethysmography or helium dilution and sGaw by plethysmography. Single-breath DLCO, VA, KCO, and VI were determined using helium as the tracer gas, except for four studies using CH_4_.

Equations for predicted mean, 5% lower limit of normal (LLN), and 5% upper limit of normal (ULN) used birth sex, height, and fractional age. 10%LLN and 10%ULN were calculated assuming normal distributions below and above the mean.

Predicted FEV1, FVC, and FEV1/FVC used GLI race-neutral equations [15] with Johnson race-neutral values also exported. Predicted values for other spirometry parameters were calculated by weighting the values from Johnson [6] as 2/3 Caucasian/Mexican American and 1/3 African-American. With NHANES and GLI not providing predicted FEF50, FEF50 predicted values are not reported.

Predicted TLC and RV used GLI equations [16] with a race-neutral factor of 0.96. For sGaw, predicted sGaw 0.23, LLN 0.12, and ULN 0.34 were used [17, 18].

Predicted DLCO used GLI equations [19], a race-neutral factor of 0.96, and was adjusted for hemoglobin [20]. Predicted VA equals predicted TLC minus predicted estimated dead space [13]. Predicted KCO equals predicted DLCO divided by predicted VA. Predicted DACO equals predicted DLCO*(0.58+0.42*(VA/predicted VA)) [21]. %predicted KACO equals %predicted DACO since predicted KACO equals predicted KCO*(.0.42+0.58/ (VA/predicted VA)). Predicted DLCO was compared to that using Crapo's equations [22], which have been used by IPF-PRO studies.

FOVE (flow-over-volume elevation) was defined as having elevated flow relative to FVC with FOVE1 elevated PEF/FVC, FOVE2 or elevated FEF25-75/FVC, and FOVE3 or elevated FEF75/FVC above the 5% ULN, and near FOVE as having ratios above the 10% ULN. For age ≥50, the ULN:10%ULN as %predicted were 135.6:127.7 for PEF/FVC, 170.5:162.0 for FEF25-75/FVC, and 282.6:242.3 for FEF75/FVC.

Race-neutral GLI spirometry prediction equations for FEV1, FVC, and FEV1/FVC were compared to those of Johnson. GLI race-neutral predicted FVC was compared to GLI predicted TLC – RV.

## 3 RESULTS

258 patients were identified as having been prescribed nintedanib or pirfenidone. Sixty-four patients were excluded: 28 without UIP, 54 without system PFTs, some also without UIP, and 1 with PFTs only following lung transplant. 194 met the inclusion criteria, of whom 182 had DLCO, with 2/3 male, over half smokers, 93 (48%) <10 pack-years, 73 (38%) >20 pack-years, with a mean age of 70.1 at first testing and 73.6 at last testing (Table **1**). UIP was diagnosed by chest CT in 190 and lung biopsy in 4, with idiopathic pulmonary fibrosis (IPF) in all but one, with Hermansky-Pudlak syndrome. Most patients had three or more spirometry and DLCO studies (Tables **2** and **3**).

Race-neutral GLI predicted FEV1, FVC, and FEV1/FVC mean, LLN, and ULN closely matched race-neutral Johnson equations (Table **4**).

FOVE1 (*p*=0.024) and near FOVE1 (*p*=0.053) were more common at last than at first testing, with other FOVE parameters more common at last than at first but *p* not significant (Table **5**). >20 pack-year smokers were less likely to have FOVE than <10 pack-year smokers, but these were not statistically significant (*p*>0.05).

Table **6** shows selected results including FOVE, near FOVE, and PRISm at first and last spirometry for all patients and for those with “normal spirometry” having FEV1, FVC, and FEV1/FVC all ≥ LLN. Flow over volume parameters (FOVE3, PEF/FVC (FOVE1), *p*<0.00001; FEF25-75/FVC, FEF75/FVC, *p*<0.01 first, *p*<0.00001 last) were much more likely to be above the upper limit of normal than flow parameters (FEV1/FVC, PEF, FEF25-75, FEF75) for all first and last studies. Among those with normal spirometry, FOVE3 and PEFR/FVC(FOVE1) were much more likely than FEV1/FVC to be above the upper limit of normal at the first (*p*< 0.0001) and last (*p*<0.0001, *p*=0.002) study.

Normal spirometry (56.9% v 48.4% first, 43.1% v 40.9% last) and lung volumes (48.1% v 39.7% first, 30.0% v 17.2% last) were more likely in heavy smokers than <10 pack-year smokers, but the differences were not significant (*p*>0.05).

At first DLCO testing, 29/137 (21.2%) of patients had normal FVC, TLC, and KCO, and of those, 6.9% had elevated FEV1/FVC, 48.3% had FOVE3, 58.6% had near FOVE3, and 37.9% had low DACO. At first DLCO testing, 15/137 (10.9%) of patients had normal FVC, TLC, and DLCO, and of those, 13.3% had elevated FEV1/FVC, 53.3% had FOVE3, and 60.0% had near FOVE3. At final testing, 5 patients had normal FVC, TLC, and KCO, and only 2 patients had normal FVC, TLC, and DLCO.

Many parameters are abnormal and show differences by smoking status and first/last study with Table **7a-7d** shows selected parameters from <10 pack-year, >20 pack-year first and last studies and (**Supplementary Tables**) including all parameters, DACOpp/FVCpp, DACOpp/TLCpp, TLCpp/FVCpp, central concavity and peripheral concavity by study timing (all/first spirometry/last spirometry/first DLCO/last DLCO) and smoking status (all/<10 pack-year/>20 pack-year).

Table **8** shows mean first and last values as well as *p*-values for comparisons of first *versus* last study and for comparisons of <10 pack-year *versus* >20 pack-year smokers of all, first, and last studies. Findings at last testing were more pronounced than at first testing for many parameters, including FVC (*p*=0.007), TLC (*p*<0.001), DLCO (*p*<0.001), and DACO (*p*<0.001). >20 pack-year smokers compared to <10 pack-year smokers had higher FEV1 (*p*=0.011), FVC (*p*<0.001), and TLC (*p*=.004), and lower flows/FVC (*p*<0.001) and sGaw (*p*=0.001)

For %predicted values, FVC was reduced more than FEV1, PEF was near normal, FEV1/FVC, FEF25-75, and FEF75 were increased, and flows/FVC were markedly increased. The expiratory curve was less concave than normal, with FEF25-75/PEF and FEF75/PEF above predicted. TLC was reduced similarly to FVC. DLCO was reduced more than FVC or TLC.

Percent predicted DACO was higher than percent predicted DLCO (DACOpp/DLCOpp 1.140 first, 1.199 last), and percent predicted KCO was much higher (KCOpp/DLCOpp 1.492 first, 1.754 last). DLCO and DACO were unlikely to be normal (13.7% first, 2.2% last; 23.6%, 5.5%) and rarely above 100% (2.2%, 0%; 2.7%, 0%). KCO was often normal (53.8% first, 26.4% last) and sometimes above 100% (17.0% first, 5.5% last).

Figure **1a-1d** shows the cumulative % of studies having %predicted FVC, PEF/FVC, TLC, and DACO values ≤ those on the x-axis for the first and last studies by smoking status. Figure **2** shows the cumulative % of studies having %predicted DLCO, DACO, and KCO values ≤ those on the x-axis for the first and last studies. **Supplementary Figures** show similar graphs of spirometry, lung volume, and DLCO parameters, and of DACO%predicted/FVC%predicted, DACO%predicted/TLC %predicted, MVV/FEV1, VI/FVC, and (VA+estimated dead space)/TLC. Most parameters had differences by smoking status and from the first to the last study.

MVV/FEV1 was 44.9±9.68 n=417 for all studies, with 69.1% having MVV/FEV1>40 without significant difference by smoking status.

Table **9** shows the first and last %predicted TLC, %<LLN, and %<80%predicted by smoking status. More patients had low TLC at last compared to first study (*p*=0.001).

DLCO-inspired volume/spirometry FVC was 0.949±0.064 n=737 for all studies, with no difference by smoking status.

VA plus estimated dead space (DSe) closely matched TLC measured by plethysmography or helium dilution, with (VA + DSe)/TLC of 0.964±0.119, n=533, without a difference by smoking status.

DACOpp/FVCpp and DACOpp/TLCpp were reduced in the first study, lower in the last study, and more reduced in heavy smokers compared to <10 pack-year smokers (*p*<0.001 for all comparisons), while TLCpp/FVCpp was not reduced (Table **10**).

Table **11** shows the % of patients having reduced FVC, TLC, DLCO, DACO, and KCO for first and last studies by smoking status, and by whether FVC is <LLN or ≥LLN or TLC is ≥LLN.

There was a wide range of yearly differences between first and last study results. Of the 150 patients having at least two DLCO studies, 120 also had first and last TLC. Some patients had higher PFT parameters on their last study compared to their first study, with 39 higher FVC, 20 higher TLC, 13 higher DLCO, and 15 higher DACO. The median, Q1, and Q3 for yearly % drops from first to last testing, having DLCO, relative % drops, and estimated years from 100% to initial value assuming constant relative % drop for FVC, TLC, DLCO, and DACO are shown in Table **12**. About 50% of patients were estimated to have taken 1.3 to 6.5 years to fall from 100% to their initial DLCO, and 75% under 6.5 years to fall from 100% to their initial FVC, TLC, DLCO, or DACO (Table **12**, Fig. **3**).

Predicted (TLC-RV)/FVC using GLI equations at last lung volume testing was 1.126±0.036 n=171.

DLCO at first testing was 54.9% predicted (median 54.6%), but using Crapo equations [22] was 42.3% predicted (median 42.7%).

## 4 DISCUSSION

This is the first study to evaluate flows relative to volume using reference equations. Most UIP patients had abnormal spirometry on first testing with FOVE or near FOVE even when FEV1, FVC, and FEV1/FVC were normal. FOVE was common when KCO was normal. Other findings include that many patients with normal FVC had restriction, significant differences in PFTs of heavy smokers, inconsistencies between GLI spirometry and lung volume equations, high sGaw, and high MVV relative to FEV1. Additionally, this study found that VA closely matches TLC, that race-neutral Johnson equations match those of GLI, and that DACO is a better measure than KCO of diffusion.

ILD increases lung stiffness and elastic recoil, leading to lower volumes and higher flows. FOVE or near FOVE was present in most patients on initial (56.7%, 68.6%) and last testing (61.3%, 70.6%) and was much more common in those with “normal spirometry” than elevated FEV1/FVC. While 5% of normal subjects are expected to have PEFR/FVC>ULN (FOVE1) and 10% >10%ULN (near FOVE1), with specificities of 95% and 90%, 38.1% had FOVE1 and 47.4% near FOVE1 (sensitivities) at first testing and 50.0% and 57.7% at last testing. Sensitivities for FOVE2 and FOVE3 were higher. Specificities for FOVE2 would be 90%-95% and for FOVE3 86%-95% depending on the correlations of PEF/FVC, FEF25-75/FVC, and FEF75/FVC. UIP patients had less concave expiratory flow-volume curves than normal. While most patients (84.5% first, 89.2% last) had FVC<predicted, many fewer (42.3% first and 56.7% last) had FVC<LLN.

High FEV1/FVC has been proposed as an indicator of early ILD [23] and increased flows relative to volume have been reported [24, 25]. This study found fewer patients with normal PFTs (FVC, TLC, and DLCO ≥ LLN) at initial testing (10.9% *vs* 30%) and a lower fraction of those having elevated FEV1/FVC (13.3% *vs* 37%) than a study of newly identified patients with ILD (8). Thus, UIP patients are more likely to have abnormal pulmonary function at initial testing than those with ILD in general. Studies are needed to evaluate FOVE in patients with other types of ILD.

With over half (53.1%) of UIP patients having “normal spirometry” at initial testing, further evaluation could be delayed if novel spirometry parameters are ignored. Of those with initial normal spirometry, FOVE3 (47.6%) and near FOVE3 (63.1%) were much more common than elevated FEV1/FVC (6.8%). Of those with initially normal FVC, TLC and KCO, FOVE3 (48.3%), near FOVE3 (58.6%), and low DACO (37.9%) were common, again emphasizing the importance of considering FOVE and DACO.

Recommendations to use race-neutral spirometry reference equations [26] were adopted after standards for PFT interpretation [5] and after many studies of PRISm, so adjustments to interpretations may be indicated. The method used for race-neutral novel spirometry equations is supported by findings that race-neutral Johnson equations matched race-neutral GLI equations for FEV1, FVC, and FEV1/FVC. There are inconsistencies in GLI spirometry and lung volume reference equations. While TLC-RV should equal FVC, GLI predicted TLC – RV was larger than GLI predicted race-neutral FVC, likely related to differences in the reference subjects. With GLI TLC–RV 12.6% higher than GLI race-neutral FVC, it is reasonable to apply a race correction factor (this study used 0.96) to GLI predicted lung volumes and DLCO to reduce this discrepancy.

Interpretation standards [5] recommend that in adults, a normal FVC and FEV_1_/FVC are highly reliable for ruling out restriction, citing a study which used non-standard spirometry and lung volume reference equations finding 2.4% of such patients had restriction, and that restriction was more common if FEV1/FVC was elevated [27]. However, using GLI race-neutral spirometry equations for FVC LLN and GLI equations for TLC LLN, a study [28] found that 14.1% of white and 34.8% of non-Hispanic blacks with normal FVC had restriction. The current study found that many UIP patients with normal FVC had restriction (34.8% first, 55.2% last). Nearly all UIP patients had TLC below 100% predicted (92.7% first, 96.2% last), and most had restriction (56.9% first, 77.7% last). Heavy smokers were less likely to have restriction. Nearly all (>95%) patients with reduced FVC had restriction. The rates of restriction would have been higher if predicted TLC had not been reduced by 4% for race correction.

PRISm [29, 30] is spirometry with reduced FEV1 (<80% predicted) without obstruction (FEV1/FVC≥70%) and has been associated with increased respiratory symptoms and mortality [31-34] and with increased odds (7% *vs* 4%) of having interstitial lung abnormalities on chest CT [35]. If race-neutral prediction equations, which have lower predicted FEV1, had been used, then fewer patients would meet criteria for PRISm. In the current study, nearly all patients had FEV1/FVC≥70%, so nearly all with FEV1 <80% met PRISm criteria. Many more UIP patients with “normal spirometry” had FOVE and near FOVE (47.6%, 63.1%) than PRISm (6.8%) or elevated FEV1/FVC (6.8%) at initial testing.

Predicted MVV is generally 35 to 40 times FEV1 [36], with lower MVV expected if significant respiratory muscle weakness or extrathoracic upper airway obstruction limits inspiratory flows. It is likely the higher flows relative to volume contribute to the higher MVV/FEV1 (44.9) found in this study.

sGaw measures conductance of the airways independent of lung volumes and is reduced in patients with significant obstruction. This study found that sGaw was elevated in UIP, but not as elevated in heavy smokers.

VA measured during DLCO is often lower than TLC in patients with COPD and close to TLC with ILD [21]. This study found that VA was very close to TLC minus estimated dead space in UIP patients, including those with heavy smoking. With VA on DLCO testing providing a very good estimate of TLC, independent measurement of lung volume provides confirmatory information in UIP patients.

Most patients with UIP had significant impairment on initial PFT, with many parameters including FEV1, FVC, lung volumes, and DLCO worsening with time. Other parameters, including FEF25-75, PEF, and FEV1/FVC, did not significantly change. There were wide variations in the rate of change of PFT parameters. Assuming a steady relative rate of change from initial normal PFTs, about 50% of patients had a 1.3 to 6.5 year delay from disease onset to initial DLCO.

An IPF-PRO Registry study [37] found initial median FVC and DLCO of 72.4% and 41.6%, which are lower than medians and means of the current study (77.0%,78.5% and 54.6%, 54.9%), suggesting that our patients were evaluated earlier. However, that study used prediction equations with higher predicted FVC and much higher predicted DLCO. Using Crapo DLCO equations, the initial DLCO was 42.3% (median 42.7%) for our patients, nearly identical to that of the Registry. Initial DLCO is an important factor to predict patient outcomes in the Registry [37]. There are no studies comparing DLCO, DACO, and patient outcomes.

The effects of emphysema on spirometry can counterbalance those of ILD, with emphysema reducing flows and increasing lung volumes, and ILD increasing flows and reducing lung volumes. Since both emphysema and ILD reduce DLCO, the presence of very low DLCO with normal spirometry and lung volumes in a patient with a heavy smoking history suggests combined emphysema and ILD, while pulmonary vascular disease is suspected if there is no heavy smoking. Fang *et al.* [38] found that patients with IPF and emphysema had higher FVC, similar FEV1, and lower FEV1/FVC than those with IPF alone. This study confirmed expectations, with heavy smokers and UIP having higher FEV1 and FVC, lower FEV1/FVC, higher lung volumes, lower sGaw, lower DACO/FVC, lower DACO/TLC, and being less likely to have FOVE.

The current study confirms that patients with UIP have low DLCO and low DACO, with KCO low, normal, or elevated. DLCO and DACO were more likely to be abnormal than any spirometry parameter, FOVE, or lung volume.

ATS/ERS interpretation standards do not include the known effects of lung volume on DLCO and KCO [5]. As VA becomes lower, DLCO decreases, and KCO increases, as expected from the membrane component of gas exchange proportional to surface area [21]. DACO allows one to evaluate whether the DLCO is lower than expected for the lung volume. While it has been argued that KCO is the best reflection of the lung’s ability to exchange gas [39], this study found that KCO was often normal (53.8% first, 26.4% last) and sometimes above 100% (17.0% first, 5.5% last). This shows that KCO is not a good indicator of diffusion. DACO, which adjusts predicted DLCO for lung volume, is a better indicator. Screening of subjects having a positive family history of IPF has been proposed, with mixed results [40]. This study supports that if spirometry shows FOVE, then further testing including DLCO should be performed.

A recommended PFT report (Fig. **4**) includes the novel parameter PEF/FVC with 95% values showing LLN for values ≤predicted, otherwise the ULN, and includes DACO. This patient with UIP had “normal spirometry”, FOVE with elevated PEF/FVC, TLC near LLN, and very reduced DLCO and DACO.

Some computer impressions we use include: “FEV1 and FVC are normal. However, increased PEF relative to FVC suggests possible interstitial lung disease.” “The finding of reduced DLCO, elevated PEF relative to FVC, and restriction suggests interstitial lung disease”, and “The finding of very reduced DLCO with normal spirometry and lung volumes with much smoking history suggests combined emphysema and interstitial lung disease”.

There are limitations to this study. Some patients only had one spirometry or DLCO, which were included as both first and last study, but analysis of rate of change only included those with more than one DLCO. Some patients, including those referred for lung transplantation, had PFTs done at other facilities, and these outside results were not included. For some patients, the last PFT was the last PFT before death, while others had PFTs after the study cut-off date. Therefore, last PFTs would have been worse if all patients had been followed to death.

## CONCLUSION

Race-neutral prediction equations for novel spirometry parameters allow assessment of FOVE (flow-over-volume elevation). This comprehensive evaluation of PFTs in patients with UIP found that FOVE and near-FOVE are common, including at first PFT and in those with normal FEV1, FVC, FEV1/FVC, and KCO. Finding FOVE should increase concern that a patient may have UIP or an interstitial lung disease, leading to checking DLCO and considering HRCT. DACO, which adjusts predicted DLCO for lung volume, is a better measure of diffusion than KCO since KCO can be low, normal, or elevated in UIP. With heavy smokers with UIP having higher FEV1, FVC, and TLC, it is important to consider smoking status along with FOVE and DACO when interpreting PFTs. Further studies are needed to determine the sensitivity of FOVE in other interstitial lung diseases and whether earlier diagnosis and treatment affect disease course.

## Figures and Tables

**Fig. (1):**
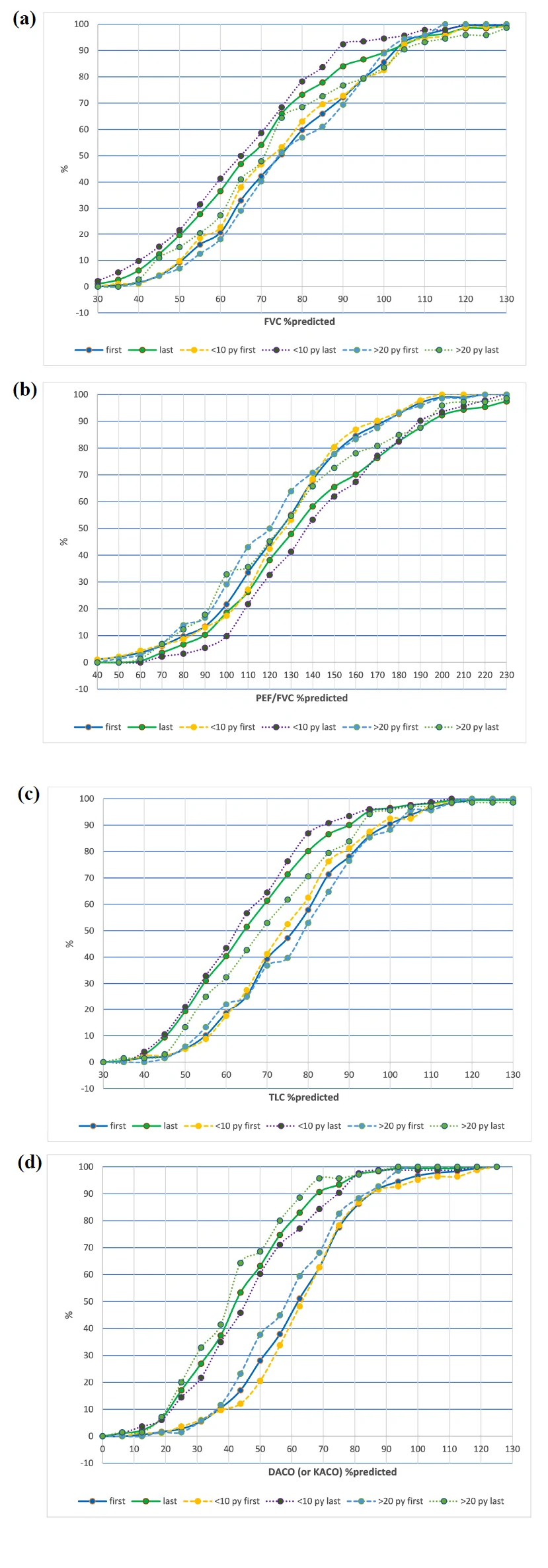
Cumulative % of studies having %predicted FVC (**a**), PEF/FVC (**b**), TLC (**c**), and DACO (**d**) values ≤ that on the x-axis for first and last studies by smoking status.

**Fig. (2):**
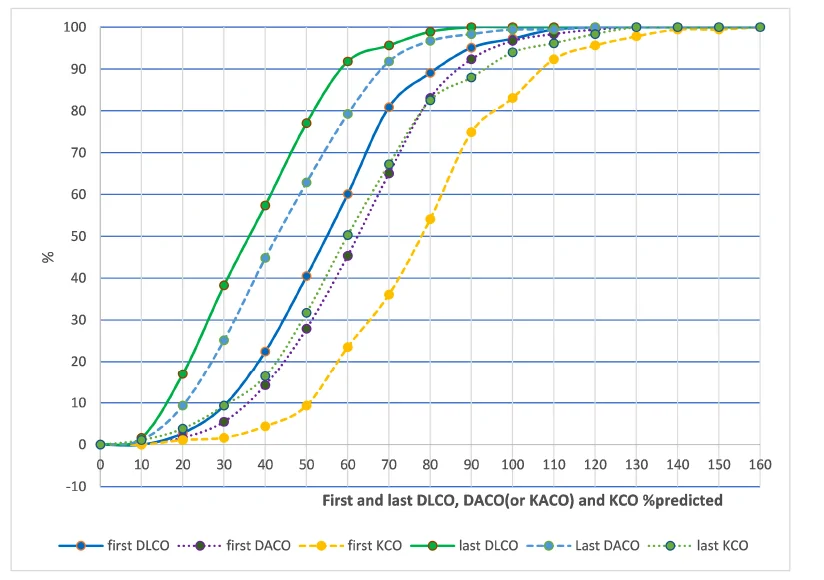
Cumulative % of studies having % predicted DLCO, DACO (or KACO), and KCO values ≤ those on the x-axis for first and last studies.

**Fig. (3):**
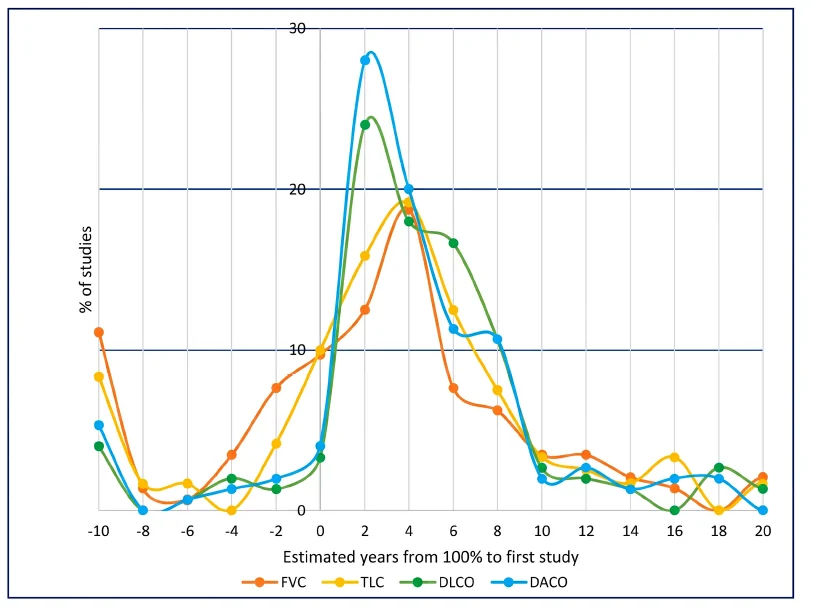
Estimated years from 100% of FVC, TLC, DLCO, and DACO to the value at first PFT with DLCO, assuming a constant relative rate of change from first to last PFT.

**Fig. (4):**
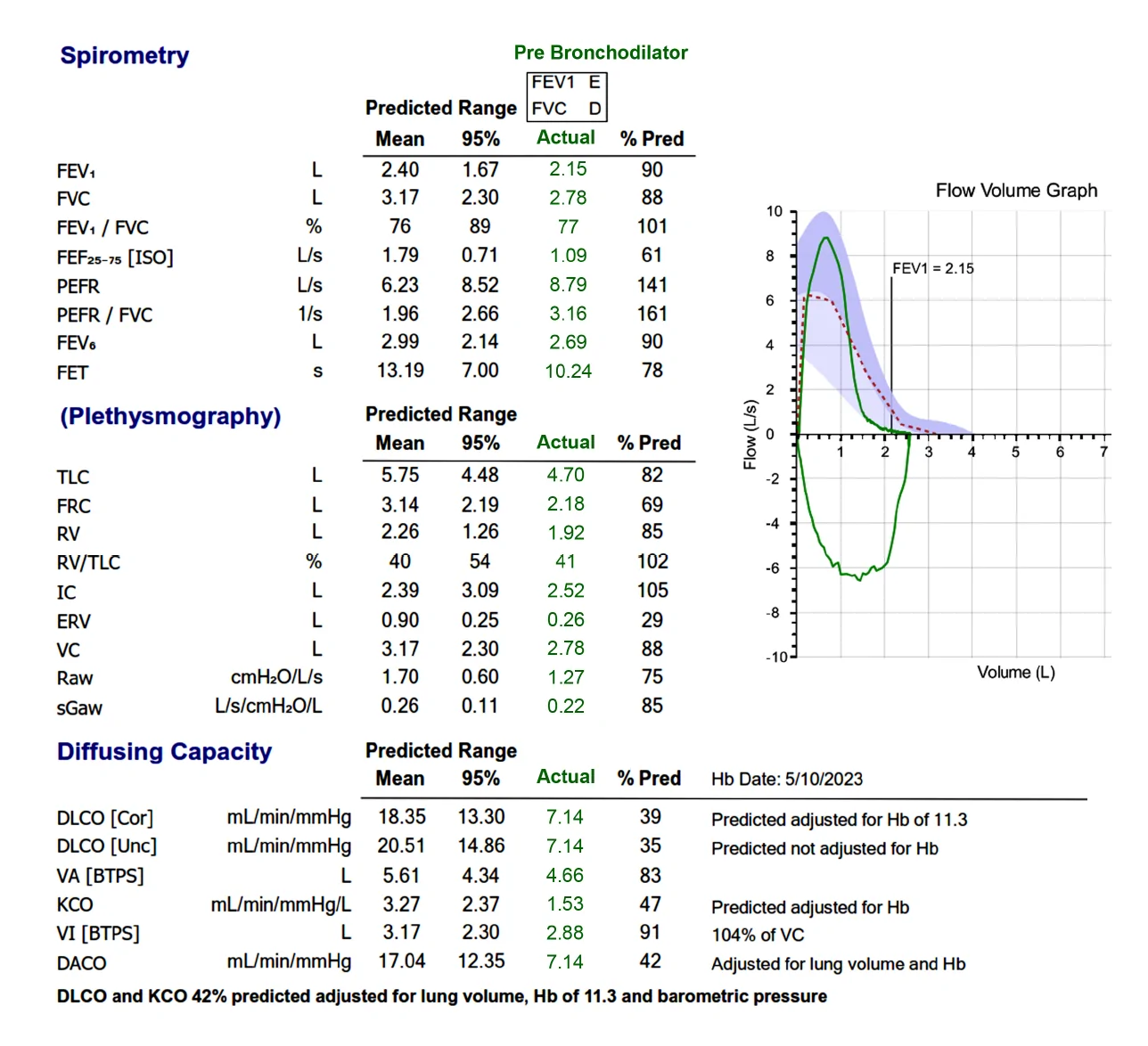
PFT report of a usual interstitial pneumonia (UIP) patient.

**Table 1:** Demographics.

-	All	-	Male	-	Female	-
-	mean	SD	mean	SD	mean	SD
age first PFT	70.1	9.2	70.4	8.0	69.4	11.2
age last PFT	73.6	8.9	73.7	7.8	73.5	10.6
BMI first PFT	30.1	6.4	29.7	5.6	30.9	7.7
BMI last PFT	28.5	6.2	28.7	5.5	28.1	7.4
-	n	%	n	%	n	%
male sex,	127	65%	-	-	-	-
female sex	67	35%	-	-	-	-
never smoker	74	38%	40	31%	34	51%
smoking <10 pk yr	93	48%	53	42%	40	60%
smoke 10-20 pk yr at first	28	14%	19	15%	9	13%
smoking >20 pk yr at first	73	38%	55	43%	18	27%
White	184	95%	124	98%	60	90%
non-Hispanic	161	83%	113	89%	48	72%
Hispanic	23	12%	11	9%	12	18%
Black	9	5%	3	2%	6	9%
Asian	1	1%	0	0%	1	1%
Total	194	-	127	-	67	-

**Table 2:** Number of spirometry and DLCO studies.

Studies per	Number of Patients		Number of Studies	
patient	spirometry	DLCO	spirometry	DLCO
1	23	29	23	29
2	40	46	80	92
3	30	26	90	78
4	18	18	72	72
5	24	14	120	70
6	10	11	60	66
7	9	14	63	98
8	14	5	112	40
9	4	8	36	72
10	9	5	90	50
11	7	3	77	33
12	3	2	36	24
13	2	1	26	13
14	1	0	14	0
total	194	182	899	737

**Table 3:** Number of PFT studies by smoking status.

PFT Studies	All	Never Smoker	<10 pk yr	10-20 pk yr	>20 pk yr
first with spirometry	194	74	93	29	72
first with DLCO	182	66	84	28	70
last with spirometry	194	74	93	28	73
last with DLCO	182	66	84	28	70
All with spirometry	899	354	442	154	303
All with DLCO	737	277	356	130	251
All with Plethysmography	504	192	242	100	162
All with sGaw	447	168	213	89	145
All with Helium dilution	48	15	18	6	24
All with MVV	417	168	209	75	133

**Table 4:** Race neutral predicteds GLI/Johnson.

-	Mean	LLN	ULN
FEV1	0.995 ± 0.040	0.980 ± 0.051	1.007 ± 0.051
FVC	1.019 ± 0.036	1.000 ± 0.030	1.011 ± 0.049
FEV1/FVC	0.969 ± 0.017	0.992 ± 0.018	0.979 ± 0.022

**Table 5:** Flow Over Volume Elevated (FOVE) first and last studies by smoking status.

Smoking Status					
-	All	never	<10 pk yr	10-20 pk yr	>20 pk yr
first FOVE1	38.1%	33.8%	37.6%	48.3%	34.7%
first FOVE2	51.5%	47.3%	50.5%	62.1%	48.6%
first FOVE3	56.7%	56.8%	60.2%	62.1%	50.0%
last FOVE1	50.0%	51.4%	54.8%	52.2%	43.8%
last FOVE2	57.2%	62.2%	64.5%	56.5%	49.3%
last FOVE3	61.3%	67.6%	69.9%	65.2%	50.7%
first near FOVE1	47.4%	47.3%	48.4%	62.1%	40.3%
first near FOVE2	62.4%	62.2%	63.4%	72.4%	56.9%
first near FOVE3	68.6%	73.0%	74.2%	72.4%	59.7%
last near FOVE1	57.7%	62.2%	65.6%	56.5%	49.3%
last near FOVE2	64.4%	70.3%	73.1%	65.2%	54.8%
last near FOVE3	70.6%	77.0%	79.6%	73.9%	60.3%

**Note:** FOVE1 - having PEF/FVC > ULN; FOVE2 - or FEF25-75 > ULN; FOVE3 - or FEF75 > ULN near FOVE - having parameters > 10% ULN Predicted ULN/mean: PEF/FVC 1.35, FEF25-75/FVC 1.75, FEF75/FVC 2.81 Predicted 10%ULN/mean: PEF/FVC 1.27, FEF25-75/FVC 1.57, FEF75/FVC 2.38

**Table 6:** Selected parameters for first and last studies, all patients and those with "normal spirometry".

	n	%	FEV1/FVC	PEF	FEF25-75	FEF75	PRISm	FOVE3	near
			>ULN	>ULN	>ULN	>ULN			FOVE3
First all	194		16.5%	9.3%	11.3%	12.9%	40.2%	56.7%	68.6%
"normal spirometry"	103	53.1%	6.8%	16.5%	14.6%	21.4%	6.8%	47.6%	63.1%
Last all	194		19.6%	12.9%	9.8%	12.4%	55.7%	61.3%	70.6%
"normal spirometry"	80	41.2%	13.8%	23.8%	12.5%	26.3%	16.3%	45.0%	56.3%

**Table:** 

	PEF/FVC	FEF25-75/FVC	FEF75/FVC	PEF/FVC	FEF25-75/FVC	FEF75/FVC
	>ULN	>ULN	>ULN	>10% ULN	>10% ULN	>10% ULN
First all	38.1%	29.4%	28.4%	47.4%	42.3%	39.2%
"normal spirometry"	31.1%	23.3%	21.4%	40.8%	36.9%	33.0%
Last all	50.0%	36.6%	33.0%	57.7%	45.4%	44.8%
"normal spirometry"	36.3%	26.3%	26.3%	45.0%	30.0%	36.3%

**Note:** “normal spirometry” - FEV1, FVC, and FEV1/FVC all ≥ LLN FOVE3 (Flow Over Volume Elevated) having PEF/FVC, FEF25-75/FVC, or FEF75/FVC > ULN Near FOVE3 – having PEF/FVC, FEF25-75/FVC, or FEF75/FVC > 10% ULN PRISm - FEV1/FVC ≥ 70%, FEV1 < 80% predicted.

**Table 7a:** Selected parameters of <10 pack year smokers from first spirometry or DLCO study.

<10 Pack Year First		-	-	-	>5%	>10%	<5%	<10%	-
parameter	Mean	SD	n	%pred	ULN	ULN	LLN	LLN	>100%
FEV1	2.04	0.64	93	80.3	3.2%	4.3%	33.3%	44.1%	20.4%
FVC	2.53	0.91	93	75.5	1.1%	1.1%	47.3%	60.2%	17.2%
FEF25-75	2.32	1.05	93	115.1	11.8%	19.4%	9.7%	17.2%	57.0%
FEF75	0.99	0.79	93	182.8	14.0%	23.7%	0.0%	7.5%	75.3%
PEF	6.14	2.14	93	94.2	7.5%	17.2%	9.7%	14.0%	38.7%
FEV1/FVC %	81.95	8.73	93	106.7	17.2%	33.3%	4.3%	6.5%	79.6%
PEF/FEV1	3.05	0.75	93	116.3	31.2%	40.9%	7.5%	7.5%	71.0%
PEF/FVC	2.50	0.65	93	125.6	37.6%	47.3%	5.4%	6.5%	82.8%
FEF25-75/FVC	0.98	0.47	93	154.1	25.8%	43.0%	3.2%	5.4%	77.4%
FEF75/FVC	0.41	0.29	93	286.5	36.6%	47.3%	0.0%	1.1%	88.2%
FEF25-75/PEF %	40.02	17.07	93	125.7	20.4%	24.7%	5.4%	8.6%	62.4%
FEF75/PEF %	17.31	12.26	93	239.7	29.0%	33.3%	0.0%	1.1%	74.2%
TLC	4.05	1.13	63	74.8	0.0%	0.0%	60.3%	69.8%	6.3%
sGaw	0.30	0.18	44	128.3	31.8%	38.6%	4.5%	15.9%	52.3%
DLCO	11.67	4.59	84	55.9	0.0%	0.0%	86.9%	90.5%	3.6%
KCO	3.08	0.91	84	81.0	2.4%	3.6%	44.0%	52.4%	16.7%
VA	3.87	1.12	84	70.3	0.0%	0.0%	69.0%	78.6%	3.6%
DACO	11.67	4.59	84	63.3	0.0%	1.2%	78.6%	86.9%	4.8%

**Note:** %pred - %predicted.

**Table 7b:** Selected parameters of >20 pack year smokers from first spirometry or DLCO study.

>20 Pack Year First		-	-	-	>5%	>10%	<5%	<10%	-
parameter	Mean	SD	n	%pred	ULN	ULN	LLN	LLN	>100%
FEV1	2.29	0.60	72	81.6	1.4%	2.8%	26.4%	40.3%	18.1%
FVC	2.89	0.81	72	77.8	0.0%	1.4%	38.9%	50.0%	13.9%
FEF25-75	2.54	1.14	72	112.3	9.7%	16.7%	6.9%	12.5%	52.8%
FEF75	0.90	0.49	72	151.0	12.5%	13.9%	0.0%	2.8%	70.8%
PEF	6.77	2.08	72	93.4	6.9%	12.5%	8.3%	20.8%	40.3%
FEV1/FVC %	80.17	7.68	72	105.6	11.1%	27.8%	2.8%	8.3%	76.4%
PEF/FEV1	3.01	0.83	72	114.4	30.6%	36.1%	8.3%	12.5%	68.1%
PEF/FVC	2.43	0.74	72	121.5	34.7%	40.3%	6.9%	9.7%	72.2%
FEF25-75/FVC	0.93	0.45	72	146.2	29.2%	38.9%	0.0%	2.8%	69.4%
FEF75/FVC	0.33	0.19	72	216.1	22.2%	31.9%	0.0%	1.4%	80.6%
FEF25-75/PEF %	39.32	16.87	72	123.9	19.4%	23.6%	2.8%	8.3%	59.7%
FEF75/PEF %	14.69	9.53	72	186.0	16.7%	19.4%	0.0%	1.4%	76.4%
TLC	4.70	1.16	52	77.9	0.0%	1.9%	51.9%	65.4%	11.5%
sGaw	0.25	0.27	36	108.6	11.1%	11.1%	11.1%	22.2%	38.9%
DLCO	11.58	4.52	70	52.6	0.0%	0.0%	87.1%	88.6%	0.0%
KCO	2.68	0.82	70	72.1	0.0%	1.4%	54.3%	61.4%	12.9%
VA	4.43	1.11	70	75.0	1.4%	1.4%	58.6%	70.0%	7.1%
DACO	11.58	4.52	70	58.0	0.0%	0.0%	80.0%	85.7%	0.0%

**Note:** %pred - %predicted.

**Table 7c:** Selected parameters of <10 pack year smokers from last spirometry or DLCO study.

<10 Pack Year Last		-	-	-	>5%	>10%	<5%	<10%	-
parameter	Mean	SD	n	%pred	ULN	ULN	LLN	LLN	>100%
FEV1	1.75	0.56	93	72.1	0.0%	0.0%	49.5%	57.0%	6.5%
FVC	2.13	0.75	93	66.2	0.0%	0.0%	58.1%	76.3%	5.4%
FEF25-75	2.19	1.02	93	117.9	10.8%	16.1%	8.6%	16.1%	60.2%
FEF75	0.87	0.64	93	181.4	17.2%	22.6%	2.2%	8.6%	68.8%
PEF	5.93	2.13	93	95.4	12.9%	17.2%	8.6%	14.0%	38.7%
FEV1/FVC %	83.14	7.64	93	108.5	19.4%	32.3%	1.1%	3.2%	87.1%
PEF/FEV1	3.43	0.83	93	129.5	48.4%	60.2%	2.2%	3.2%	84.9%
PEF/FVC	2.84	0.74	93	142.9	54.8%	65.6%	1.1%	4.3%	90.3%
FEF25-75/FVC	1.10	0.57	93	178.5	36.6%	44.1%	2.2%	4.3%	83.9%
FEF75/FVC	0.45	0.37	93	380.4	39.8%	51.6%	1.1%	1.1%	91.4%
FEF25-75/PEF %	39.01	17.70	93	127.5	16.1%	22.6%	6.5%	9.7%	63.4%
FEF75/PEF %	16.38	13.21	93	323.8	26.9%	32.3%	1.1%	1.1%	81.7%
TLC	3.52	1.03	58	64.4	0.0%	0.0%	82.8%	89.7%	5.2%
sGaw	0.38	0.51	51	165.6	35.3%	39.2%	0.0%	13.7%	60.8%
DLCO	7.87	3.56	84	38.7	0.0%	0.0%	97.6%	100.0%	0.0%
KCO	2.49	0.94	84	66.7	0.0%	1.2%	63.1%	72.6%	9.5%
VA	3.25	0.95	84	59.3	0.0%	0.0%	88.1%	91.7%	0.0%
DACO	7.87	3.56	84	46.2	0.0%	0.0%	91.7%	96.4%	0.0%

**Note:** %pred - %predicted

**Table 7d:** Selected parameters of >20 pack year smokers from last spirometry or DLCO study.

>20 Pack Year Last		-	-	-	>5%	>10%	<5%	<10%	-
parameter	Mean	SD	n	%pred	ULN	ULN	LLN	LLN	>100%
FEV1	2.04	0.60	73	77.4	1.4%	2.7%	39.7%	52.1%	16.4%
FVC	2.57	0.83	73	73.7	2.7%	2.7%	53.4%	63.0%	16.4%
FEF25-75	2.29	1.07	73	113.2	9.6%	15.1%	6.8%	11.0%	52.1%
FEF75	0.81	0.44	73	152.8	11.0%	16.4%	1.4%	1.4%	68.5%
PEF	6.32	2.14	73	92.3	9.6%	20.5%	15.1%	24.7%	37.0%
FEV1/FVC %	80.38	8.95	73	105.9	19.2%	32.9%	4.1%	6.8%	75.3%
PEF/FEV1	3.16	0.87	73	119.0	39.7%	45.2%	6.8%	11.0%	68.5%
PEF/FVC	2.56	0.85	73	128.4	43.8%	49.3%	4.1%	9.6%	65.8%
FEF25-75/FVC	0.97	0.56	73	159.6	34.2%	37.0%	2.7%	5.5%	72.6%
FEF75/FVC	0.34	0.21	73	251.3	26.0%	35.6%	0.0%	0.0%	87.7%
FEF25-75/PEF %	37.47	14.61	73	122.9	16.4%	21.9%	2.7%	11.0%	65.8%
FEF75/PEF %	13.47	6.64	73	191.8	15.1%	20.5%	0.0%	1.4%	79.5%
TLC	4.15	1.00	50	70.7	0.0%	0.0%	70.0%	78.0%	4.0%
sGaw	0.29	0.29	39	125.7	17.9%	20.5%	17.9%	28.2%	51.3%
DLCO	7.61	3.38	70	35.3	0.0%	0.0%	98.6%	98.6%	0.0%
KCO	2.01	0.75	70	55.0	0.0%	0.0%	84.3%	88.6%	1.4%
VA	3.83	1.00	70	65.2	0.0%	0.0%	80.0%	81.4%	1.4%
DACO	7.61	3.38	70	40.9	0.0%	0.0%	97.1%	98.6%	0.0%

**Note:** %pred - %predicted

**Table 8:** Means of first and last studies, *p*-values for comparisons of first *versus* last study, and for comparisons of <10 pack-year *versus* >20 pack-year smokers of all, first, and last studies.

-	Mean	Mean	*p*-value	-	*p*-values	-
-	first	last	first *vs* last	<10 *vs* >20 pack year		
parameter	-	-	study	all	first	last
FEV05 pp	86.3	79.6	0.003	0.232	0.339	0.755
FEV1 pp	80.9	74.4	0.017	0.011	0.638	0.209
FEV3 pp	81.6	73.4	0.004	0.005	0.430	0.146
FEV6 pp	81.0	75.4	0.059	0.068	0.860	0.718
FVC pp	76.5	69.4	0.007	<0.001	0.430	0.434
FEF25-75 pp	115.2	115.6	0.919	0.020	0.753	0.214
FEF75 pp	166.8	164.9	0.127	0.070	0.209	0.275
PEF pp	95.3	95.5	0.416	0.368	0.209	0.876
FEV05/FVC pp	110.7	113.4	0.417	<0.001	0.084	0.042
FEV1/FEV3 pp	102.0	103.3	0.662	<0.001	0.141	0.095
FEV1/FVC %	81.1	81.9	0.917	<0.001	0.038	0.027
FEV1/FVC pp	106.2	107.3	0.539	0.002	0.341	0.156
FEV3/FVC %	94.1	93.3	0.342	<0.001	0.273	0.256
FEV6/FVC %	98.0	97.5	0.465	0.123	0.289	0.260
PEF/FEV1 pp	116.9	127.5	0.011	<0.001	0.531	0.114
PEF/FVC pp	125.4	139.3	0.104	<0.001	0.432	0.087
FEF25-75/FVC pp	151.6	169.5	0.346	<0.001	0.056	0.013
FEF75/FVC pp	249.1	310.2	0.760	<0.001	0.059	0.350
FEF25-75/PEF pp	124.1	123.4	0.755	0.368	0.874	0.872
FEF75/PEF pp	208.5	251.0	0.758	0.823	0.112	0.058
TLC pp	75.9	66.1	<0.001	0.004	0.102	0.231
RV pp	77.7	67.5	<0.001	0.734	0.413	0.735
FRC pp	83.8	77.4	0.056	0.167	0.861	0.732
sGaw pp	118.5	145.2	0.264	0.001	0.069	0.524
DLCO pp	54.9	37.1	<0.001	0.562	0.188	0.621
KCO pp	77.9	61.2	<0.001	<0.001	0.001	0.006
VA pp	72.0	61.7	<0.001	<0.001	0.024	0.035
DACO pp	61.6	43.7	<0.001	0.238	0.187	0.023
DACOpp/FVCpp	0.82	0.65	<0.001	0.021	0.031	0.006
DACOpp/TLCpp	0.85	0.68	<0.001	0.006	0.015	0.081
TLCpp/FVCpp	0.96	0.92	0.013	0.762	0.557	0.336
(VA+Dse)/TLC	0.98	0.98	0.529	0.241	1.000	1.000
VI/FVC	0.95	0.95	0.529	0.489	0.865	0.501
MVV/FEV1	41.80	43.72	0.060	0.006	0.207	0.474

**Abbreviation:** pp - %predicted, % - the ratio * 100, otherwise the ratio.

**Table 9:** TLC - %predicted (mean, SD, n), % <LLN, and %< 80% predicted at first and last study having DLCO by smoking status.

-	First	-	-	Last	-	-
-	mean, SD, n	%<LLN	%<80% pred	mean, SD, n	%<LLN	<80% pred
all	75.0±17.6 n=138	56.9%	57.7%	64.3±17.7 n=130	77.7%	80.8%
<10 py	74.1±17.7 n=63	60.3%	60.3%	62.6±17.2 n=58	82.8%	86.2%
>20 py	76.5±18.5 n=52	51.9%	53.8%	68.5±18.0 n=50	70.0%	72.0%

**Note:** More patients had TLC <LLN (*p*=0.005) and <80% predicted (*p*=0.001) at last *versus* first study. There were not significant differences (*p*>0.05) by smoking status.

**Table 10:** Ratios of DACOpp/FVCpp, DACOpp/TLCpp, and TLCpp/FVCpp.

-	All	<10 Pack-Year	>20 Pack-Year
DACO pp/FVC pp	-	-	-
all	0.75 ± 0.27 n=737	0.79 ± 0.38 n=356	0.70 ± 0.25 n=251
first	0.82 ± 0.28 n=182	0.85 ± 0.28 n=84	0.75 ± 0.25 n=70
last	0.65 ± 0.28 n=182	0.70 ± 0.29 n=84	0.58 ± 0.26 n=70
DACO pp/TLC pp	-	-	-
all	0.78 ± 0.25 n=533	0.81 ± 0.40 n=248	0.72 ± 0.25 n=185
first	0.85 ± 0.28 n=138	0.88 ± 0.26 n=63	0.78 ± 0.28 n=52
last	0.68 ± 0.27 n=130	0.74 ± 0.29 n=58	0.60 ± 0.26 n=50
TLC pp/FVC pp	-	-	-
all	0.96 ± 0.17 n=533	0.98 ± 0.43 n=248	0.95 ± 0.14 n=185
first	0.96 ± 0.15 n=138	0.96 ± 0.17 n=63	0.95 ± 0.13 n=52
last	0.92 ± 0.18 n=130	0.94 ± 0.21 n=58	0.92 ± 0.15 n=50

**Table 11:** FVC, TLC, DLCO, DACO, KCO relations by smoking status at first and last study that included DLCO.

-	First DLCO			Last DLCO		
smoking status	all	<10 py	>20 py	all	<10 py	>20 py
FVC<LLN	26.4%	29.8%	21.4%	39.6%	39.3%	35.7%
TLC<LLN	56.9%	60.3%	51.9%	77.7%	82.8%	70.0%
DLCO<LLN	86.3%	86.9%	87.1%	97.8%	97.6%	98.6%
DACO<LLN	76.4%	78.6%	80.0%	94.5%	91.7%	97.1%
KCO<LLN	46.2%	44.0%	54.3%	73.6%	63.1%	84.3%
FVC<LLN and TLC<LLN	25.8%	28.6%	21.4%	37.9%	36.9%	34.3%
any of FVC, TLC, DLCO<LLN	89.1%	88.9%	90.4%	99.2%	98.3%	100.0%
of FVC<LLN, TLC<LLN	97.9%	96.0%	100.0%	95.8%	93.9%	96.0%
of FVC<LLN, DLCO<LLN	97.0%	94.1%	100.0%	98.0%	95.8%	100.0%
of FVC<LLN, DACO<LLN	86.6%	82.4%	95.5%	97.0%	93.8%	100.0%
of FVC<LLN, KCO<LLN	40.3%	26.5%	63.6%	69.3%	56.3%	78.4%
of FVC≥LLN, TLC<LLN	34.8%	36.8%	32.4%	55.2%	68.0%	44.0%
of FVC≥LLN, DLCO<LLN	80.0%	82.0%	81.3%	96.3%	97.2%	97.0%
of FVC≥LLN, DACO<LLN	70.4%	76.0%	72.9%	90.1%	86.1%	93.9%
of FVC≥LLN, KCO<LLN	49.6%	56.0%	50.0%	77.8%	69.4%	90.9%
of TLC≥LLN, DLCO<LLN	81.6%	80.4%	88.4%	96.3%	97.2%	97.1%
of TLC≥LLN, DACO<LLN	74.8%	76.1%	79.1%	91.4%	86.1%	97.1%

**Abbreviation:** py – pack year smoking.

**Table 12:** Median, Q1, and Q3 for yearly %drops from first to last testing having DLCO, yearly relative %drops, and estimated years from 100% to parameter at first testing.

-	Yearly %drop	Yearly Relative %drop	Estimated Years from 100%
FVC	2.13 (-0.41, 4.71)	3.16 (-0.43, 7.42)	2.27 (-1.67, 6.54)
TLC	3.28 (1.11, 6.59)	5.05 (1.41, 10.00)	2.78 (-0.10, 6.31)
DLCO	5.15 (2.58, 9.13)	12.25 (5.13, 21.41)	3.21 (1.31, 6.53)
DACO	5.40 (2.70, 9.24)	11.02 (4.94, 19.86)	2.68 (1.00, 6.30)

## Data Availability

All the data and supporting material are available within the article.

## LIST OF ABBREVIATIONS

CT: Computerized tomography
Predicted DACO: Predicted DLCO also adjusted for lung volume
DLCO: Diffusing capacity of carbon monoxide
DSe: Estimated dead space
FEF25–75%: Forced expiratory flow at 25–75% of FVC
FEF50%, FEF75%: The flow at 50% or 75% of FVC
FEV05, FEV1, FEV3, FEV6: The expiratory volume in the first 0.5, 1, 3, or 6 seconds of a FVC maneuver
FEVPEF: The expiratory volume at PEF
FET: Forced expiratory time
FOVE: Flow-over-volume elevation
FRC: Functional residual capacity
FVC: Forced vital capacity
GLI: Global Lung Initiative
LLN: Lower limit of normal
Predicted KACO: Predicted KCO also adjusted for lung volume
KCO: DLCO/ VA
HRCT: High-resolution computed tomography
ILD: Interstitial lung disease
MVV: Maximal voluntary ventilation
NHANES: National Health and Nutrition Examination Survey
PEF: Peak expiratory flow
PIF: Peak inspiratory flow
PFT: Pulmonary function testing
PRISm: Preserved ratio impaired spirometry
RV: Residual Volume
SD: Standard deviation
sGaw: Specific conductance
TLC: Total lung capacity
UIP: Usual interstitial pneumonia
ULN: Upper limit of normal
VA: Alveolar volume
VI: Inspired volume during DLCO maneuver

## STANDARDS OF REPORTING

STROBE guidelines were followed.
