# Supplementary material for: Flow-over-volume elevation (FOVE) on Spirometry Allows for Early Recognition of Usual Interstitial Pneumonia
Source: Open Respir Med J. 2026 Sep 24;20:e18743064498371. doi: 10.2174/0118743064498371260922043417 (PMC13756048; doi:10.2174/0118743064498371260922043417)
Supplement: Supplementary file 1 [file TORMJ-20-E18743064498371_SD1.pdf]

The Open Respiratory Medicine Journal
DOI: 10.2174/0118743064498371260922043417, 2026, 20, e18743064498371
ISSN: 1874-3064

SUPPLEMENTARY MATERIAL

OPEN ACCESS

Flow-over-volume elevation (FOVE) on Spirometry Allows for Early Recognition of Usual Interstitial Pneumonia

Douglas C. Johnson[1,*]

[1]Department of Medicine, Baystate Medical Center, UMass Chan Medical School, Springfield, United States of America

© 2026 The Author(s). Published by Bentham Open.
This is an open access article distributed under the terms of the Creative Commons Attribution 4.0 International Public License (CC-BY 4.0), a copy of which is available at: https://creativecommons.org/licenses/by/4.0/legalcode. This license permits unrestricted use, distribution, and reproduction in any medium, provided the original author and source are credited.

*Address correspondence to this author at the Department of Medicine, Baystate Medical Center, 759 Chestnut Street, Springfield, MA 01199, United States of America;
E-mails: dougjohnsonmd@gmail.com, douglas.johnsonmd@baystatehealth.org

Cite as: Johnson DC. Flow-over-volume elevation (FOVE) on Spirometry Allows for Early Recognition of Usual Interstitial Pneumonia. Open Respir Med J, 2026; 20: e18743064498371.
http://dx.doi.org/10.2174/0118743064498371260922043417

Received: March 05, 2026
Revised: June 04, 2026
Accepted: June 15, 2026
Published: September 24, 2026

Send Orders for Reprints to reprints@benthamscience.net

Supplementary Tables All parameters by study timing (all/first spirometry/last spirometry/first DLCO/last DLCO) and smoking status (all/<10 pack-year/>20 pack-year)

Table S1. Parameters from all studies.

all, all	-	-	-	-	>5%	>10%	<5%	<10%	-
	Mean	SD	n	% pred	ULN	ULN	LLN	LLN	≥100%
FEVPEF	0.61	0.24	819	-	-	-	-	-	-
FEV05	1.73	0.54	899	85.1	3.6%	5.7%	21.7%	34.8%	27.1%
FEV1	2.07	0.64	899	79.7	1.4%	3.3%	34.1%	45.5%	18.5%
FEV3	2.40	0.75	879	79.7	0.8%	1.9%	38.5%	49.9%	19.2%
FEV6	2.61	0.79	727	80.6	1.0%	1.7%	36.6%	47.9%	19.8%
FVC	2.57	0.84	899	75.2	0.9%	1.0%	43.8%	55.2%	14.7%
FEF25-75	2.32	1.07	899	113.3	10.0%	16.0%	8.1%	16.5%	56.4%
FEF50	3.24	1.44	899	-	-	-	-	-	-
FEF75	0.84	0.52	899	154.8	10.2%	16.7%	1.6%	6.3%	69.5%
PEF	6.67	2.36	899	99.4	14.7%	21.1%	8.7%	16.4%	47.5%
PIF	4.00	1.42	899	-	-	-	-	-	-
FET	7.67	2.82	899	-	-	-	-	-	-
FEVPEF/FVC %	24.43	7.92	819	-	-	-	-	-	-
FEV05/FVC %	68.26	9.62	899	110.9	27.7%	40.2%	3.4%	5.0%	80.3%
FEV1/FEV3 %	86.53	5.74	879	102.5	17.2%	34.1%	5.9%	7.6%	76.6%
FEV1/FVC %	81.15	7.58	899	106.3	11.8%	26.9%	2.4%	5.5%	79.8%
FEV3/FVC %	93.38	5.14	879	104.9	13.1%	26.8%	0.9%	1.8%	83.7%
FEV6/FVC %	97.54	4.98	727	104.1	5.1%	38.1%	1.5%	2.5%	83.8%
PEF/FEV1	3.28	0.89	899	124.6	43.6%	50.9%	5.2%	7.0%	75.6%
PEF/FVC	2.67	0.79	899	133.9	46.7%	53.8%	3.1%	5.3%	80.1%
FEF25-75/FVC	0.95	0.45	899	150.2	26.9%	37.4%	1.2%	4.9%	74.3%
FEF50/FVC	1.13	0.36	899	-	-	-	-	-	-

(Table) contd.....

all, all	-	-	-	-	>5%	>10%	<5%	<10%	-
FEF75/FVC	0.35	0.10	899	239.0	24.8%	35.0%	0.4%	1.1%	84.4%
FEF25-75/PEF %	36.81	15.76	899	116.3	12.0%	18.5%	6.5%	13.7%	58.2%
FEF50/PEF %	51.31	21.04	899	-	-	-	-	-	-
FEF75/PEF %	13.75	9.17	899	193.6	14.5%	18.9%	1.3%	3.9%	73.7%
PIF/PEF	0.63	0.22	899	-	-	-	-	-	-
TLC	4.07	1.15	551	72.4	0.2%	0.5%	65.5%	75.7%	4.9%
RV	1.48	0.57	552	74.8	0.7%	2.4%	34.8%	49.5%	14.9%
FRC	2.53	0.81	552	82.8	1.3%	2.9%	31.9%	46.2%	22.8%
sGaw	0.27	0.25	447	109.8	18.6%	23.9%	8.3%	17.2%	48.5%
DLCO	10.27	4.19	737	48.5	0.0%	0.0%	92.4%	95.9%	0.5%
KCO	2.72	0.87	737	72.2	0.7%	1.5%	56.4%	67.0%	11.7%
VA	3.84	1.10	737	68.1	0.3%	0.3%	73.0%	81.7%	2.4%
DACO	10.27	4.19	737	55.4	0.0%	0.1%	84.4%	90.9%	0.7%
DACOpp/FVCpp	0.75	0.27	737	-	-	-	-	-	-
DACOpp/TLCpp	0.78	0.25	533	-	-	-	-	-	-
TLCpp/FVCpp	0.96	0.17	533	-	-	-	-	-	-
central convexity	21.99	29.83	899	-	-	-	-	-	-
peripheral convexity	57.84	28.46	899	-	-	-	-	-	-

Abbreviations: DACO – %predicted DLCO with predicted DLCO also adjusted for lung volume; DLCO – diffusing capacity of the lung of carbon monoxide; FEF50, FEF75 – forced expiratory flow at 50% and at 75% of FVC; FEF25-75% - forced expired flow from 25 to 75% of FVC; FET – forced expired time; FEV05, FEV1, FEV3, FEV6 - forced expired volume in 0.5, 1, 3, and 6 seconds, FEVPEF – forced expired volume at PEF; FRC – functional residual capacity; FVC – forced vital capacity; KCO – DLCO/VA; LLN – lower limit of normal, MVV – maximal voluntary ventilation; n – number of studies; PEF – peak expiratory flow; PIF – peak inspiratory flow; pp - %predicted; py – pack-year of smoking; RV – residual volume; SD – standard deviation; sGaw – specific conductance; TLC – total lung capacity; ULN – upper limit of normal; VA – alveolar volume (as determined from tracer gas during DLCO testing); VI – inspired volume during DLCO testing

Table S2. Parameters from all studies of <10 pack-year smoking.

all <10 py	-	-	-	-	>5%	>10%	<5%	<10%	-
	Mean	SD	n	% pred	ULN	ULN	LLN	LLN	≥100%
FEVPEF	0.59	0.33	397	-	-	-	-	-	-
FEV05	1.64	0.81	442	84.1	3.6%	7.0%	25.3%	38.5%	26.9%
FEV1	1.95	0.97	442	77.9	2.3%	3.6%	38.9%	49.8%	16.7%
FEV3	2.24	1.15	431	77.6	1.4%	2.3%	44.5%	55.5%	18.8%
FEV6	2.43	1.34	341	78.3	1.8%	1.8%	42.8%	52.5%	17.6%
FVC	2.39	1.23	442	72.6	1.1%	1.1%	50.0%	60.2%	12.7%
FEF25-75	2.25	1.27	442	114.7	10.2%	15.8%	8.6%	17.2%	58.4%
FEF50	3.13	1.73	442	-	-	-	-	-	-
FEF75	0.84	0.60	442	162.1	10.6%	17.9%	1.1%	7.5%	69.7%
PEF	6.37	3.31	442	99.1	14.7%	20.8%	10.2%	15.6%	47.7%
PIF	3.80	2.00	442	-	-	-	-	-	-
FET	7.43	3.94	442	-	-	-	-	-	-
FEVPEF/FVC %/FVC %	25.74	10.64	397	-	-	-	-	-	-
FEV05/FVC %	69.60	28.71	442	113.0	28.3%	43.7%	1.6%	2.7%	85.3%
FEV1/FEV3 %	87.25	36.21	431	103.4	18.6%	37.6%	3.0%	4.4%	80.0%
FEV1/FVC %	82.27	33.25	442	107.5	14.0%	29.2%	1.1%	3.2%	83.9%
FEV3/FVC %	93.98	39.00	431	105.6	17.4%	31.6%	1.4%	1.9%	87.7%
FEV6/FVC %	97.67	47.62	341	104.7	5.6%	38.4%	2.1%	2.9%	88.0%
PEF/FEV1	3.33	1.53	442	126.3	47.3%	55.0%	4.3%	6.1%	76.9%
PEF/FVC	2.73	1.27	442	137.0	51.1%	59.7%	2.3%	4.1%	83.7%
FEF25-75/FVC	1.00	0.58	442	157.0	27.1%	40.0%	1.4%	3.6%	79.2%
FEF50/FVC	1.18	0.51	442	-	-	-	-	-	-
FEF75/FVC	0.38	0.28	442	270.5	29.9%	41.6%	0.7%	1.1%	87.3%
FEF25-75/PEF %	37.83	21.07	442	119.4	13.6%	19.7%	6.8%	12.7%	60.9%
FEF50/PEF %	52.52	25.41	442	-	-	-	-	-	-
FEF75/PEF %	14.73	11.07	442	222.1	18.1%	23.5%	0.9%	3.8%	75.6%

(Table) contd.....

all <10 py	-	-	-	-	>5%	>10%	<5%	<10%	-
PIF/PEF	0.63	0.27	442	-	-	-	-	-	-
TLC	3.75	1.77	260	70.4	0.0%	0.0%	70.4%	78.1%	5.4%
RV	1.44	0.76	259	74.7	0.4%	3.1%	34.7%	46.7%	14.7%
FRC	2.39	1.17	260	81.8	0.4%	2.3%	31.2%	45.4%	22.3%
sGaw	0.31	0.25	213	133.2	24.4%	30.0%	7.0%	15.0%	54.5%
DLCO	10.04	5.87	356	48.7	0.0%	0.0%	91.0%	95.5%	0.8%
KCO	2.84	1.54	356	75.5	1.1%	2.0%	53.1%	62.4%	15.4%
VA	3.60	1.94	356	65.3	0.3%	0.3%	77.8%	83.1%	1.4%
DACO	10.04	5.87	356	56.2	0.0%	0.3%	82.6%	89.0%	1.1%
DACOpp/FVCpp	0.79	0.38	356	-	-	-	-	-	-
DACOpp/TLCpp	0.81	0.40	248	-	-	-	-	-	-
TLCpp/FVCpp	0.98	0.43	248	-	-	-	-	-	-
central convexity	20.85	29.91	338	-	-	-	-	-	-
peripheral convexity	55.18	35.00	409	-	-	-	-	-	-

Table S3. Parameters from all studies of >20 pack-year smoking.

all >20 py	-	-	-	-	>5%	>10%	<5%	<10%	-
	Mean	SD	n	%pred	ULN	ULN	LLN	LLN	>100%
FEVPEF	0.64	0.31	280	-	-	-	-	-	-
FEV05	1.82	0.55	303	84.7	1.1%	2.6%	16.5%	30.7%	23.8%
FEV1	2.20	0.64	303	80.8	0.7%	2.3%	27.7%	41.6%	17.5%
FEV3	2.59	-	296	81.7	-	0.7%	30.4%	46.3%	18.9%
FEV6	2.79	1.20	261	82.2	0.3%	1.5%	30.7%	46.0%	20.7%
FVC	2.79	0.82	303	77.7	0.7%	1.3%	37.6%	52.5%	14.5%
FEF25-75	2.38	1.21	303	109.9	7.5%	17.2%	8.9%	17.8%	49.5%
FEF50	3.34	1.66	303	-	-	-	-	-	-
FEF75	0.84	0.51	303	146.7	7.5%	16.5%	1.3%	4.0%	66.7%
PEF	6.68	2.27	303	94.6	6.6%	16.8%	9.6%	21.8%	39.9%
PIF	4.01	1.35	303	-	-	-	-	-	-
FET	7.91	2.76	303	-	-	-	-	-	-
FEVPEF/FVC %	23.30	8.32	280	-	-	-	-	-	-
FEV05/FVC %	65.93	11.31	303	107.4	17.9%	33.0%	6.3%	8.9%	70.0%
FEV1/FEV3 %	85.30	14.90	296	101.1	12.8%	29.7%	10.5%	13.5%	66.6%
FEV1/FVC %	79.45	9.40	303	104.5	7.2%	25.7%	5.0%	9.9%	71.9%
FEV3/FVC %	92.55	15.42	296	104.0	7.2%	22.6%	0.7%	2.4%	78.7%
FEV6/FVC %	97.52	34.12	261	103.3	2.3%	35.2%	1.1%	2.7%	80.1%
PEF/FEV1	3.08	0.85	303	116.9	23.3%	39.3%	8.3%	10.2%	70.3%
PEF/FVC	2.47	0.78	303	123.5	24.0%	40.6%	5.3%	8.6%	71.6%
FEF25-75/FVC	0.89	0.46	303	141.6	18.3%	32.0%	1.0%	6.6%	65.0%
FEF50/FVC	1.05	0.30	303	-	-	-	-	-	-
FEF75/FVC	0.32	0.19	303	210.3	14.3%	29.7%	0.3%	1.7%	79.9%
FEF25-75/PEF %	36.59	15.52	303	116.3	8.6%	20.1%	5.0%	14.5%	56.8%
FEF50/PEF %	51.20	21.20	303	-	-	-	-	-	-
FEF75/PEF %	13.36	7.95	303	173.3	8.6%	16.5%	0.7%	2.3%	73.9%
PIF/PEF	0.64	0.24	303	-	-	-	-	-	-
TLC	4.47	1.17	186	75.9	0.4%	1.6%	60.8%	71.5%	6.5%
RV	1.55	0.59	186	77.9	1.2%	2.7%	36.0%	53.8%	15.6%
FRC	2.71	0.81	186	85.6	1.5%	3.8%	29.6%	45.2%	25.8%
sGaw	0.23	0.17	145	99.8	8.5%	15.2%	13.1%	24.1%	37.2%
DLCO	10.25	5.43	251	47.1	0.0%	0.0%	94.0%	96.0%	0.0%
KCO	2.50	1.19	251	67.0	0.0%	0.8%	62.5%	72.5%	7.2%
VA	4.14	1.85	251	71.4	0.3%	0.4%	70.1%	80.1%	4.0%
DACO	10.25	5.43	251	53.0	0.0%	0.0%	87.6%	93.6%	0.0%
DACOpp/FVCpp	0.70	0.26	251	-	-	-	-	-	-

(Table) contd.....

all >20 py	-	-	-	-	>5%	>10%	<5%	<10%	-
DACOpp/TLCpp	0.72	0.26	185	-	-	-	-	-	-
TLCpp/FVCpp	0.95	0.16	185	-	-	-	-	-	-
central concavity	21.51	29.57	228	-	-	-	-	-	-
peripheral concavity	58.66	25.19	292	-	-	-	-	-	-

Table S4. Parameters from all first spirometry studies.

first spiro all	-	-	-	-	>5%	>10%	<5%	<10%	-
	Mean	SD	n	% pred	ULN	ULN	LLN	LLN	≥100%
FEVPEF	0.65	0.25	159	-	-	-	-	-	-
FEV05	1.80	0.53	194	86.3	4.1%	5.7%	19.6%	29.9%	27.3%
FEV1	2.16	0.63	194	80.9	2.1%	3.6%	30.4%	41.2%	19.6%
FEV3	2.52	0.75	192	81.6	1.0%	2.1%	34.4%	44.8%	22.9%
FEV6	2.71	0.83	154	81.0	1.9%	2.6%	35.7%	46.8%	21.4%
FVC	2.70	0.87	194	76.5	0.5%	1.0%	42.3%	54.1%	15.5%
FEF25-75	2.46	1.11	194	115.2	11.3%	18.6%	8.2%	15.5%	56.7%
FEF50	3.39	1.44	194	-	-	-	-	-	-
FEF75	0.95	0.65	194	166.8	12.9%	18.0%	0.0%	5.7%	72.7%
PEF	6.55	2.22	194	95.3	9.3%	16.0%	8.8%	16.5%	42.3%
PIF	3.86	1.43	194	-	-	-	-	-	-
FET	7.42	2.97	194	-	-	-	-	-	-
FEVPEF/FVC %	25.24	8.62	159	-	-	-	-	-	-
FEV05/FVC %	68.13	10.65	194	110.7	28.9%	43.3%	6.2%	7.2%	79.4%
FEV1/FEV3 %	86.15	7.20	192	102.0	17.2%	37.5%	8.3%	9.4%	74.0%
FEV1/FVC %	81.13	8.49	194	106.2	14.4%	29.9%	4.1%	7.7%	79.4%
FEV3/FVC %	94.10	7.66	192	105.5	19.8%	32.3%	1.6%	3.1%	83.9%
FEV6/FVC %	98.03	7.51	154	104.6	8.4%	40.9%	1.9%	5.2%	83.8%
PEF/FEV1	3.07	0.77	194	116.9	33.0%	41.2%	7.2%	8.8%	71.6%
PEF/FVC	2.50	0.69	194	125.4	38.1%	46.9%	5.7%	7.2%	79.4%
FEF25-75/FVC	0.96	0.45	194	151.6	27.8%	41.8%	1.5%	4.6%	75.3%
FEF50/FVC	1.10	0.35	194	-	-	-	-	-	-
FEF75/FVC	0.37	0.24	194	249.1	28.4%	38.7%	0.0%	1.0%	85.1%
FEF25-75/PEF %	39.47	16.50	194	124.1	18.6%	22.7%	4.1%	8.8%	62.9%
FEF50/PEF %	54.07	20.37	194	-	-	-	-	-	-
FEF75/PEF %	15.69	10.64	194	208.5	21.6%	24.7%	0.0%	1.5%	76.3%
PIF/PEF	0.62	0.25	194	-	-	-	-	-	-
TLC	4.29	1.21	93	75.0	0.0%	1.1%	60.2%	72.0%	7.5%
RV	1.52	0.52	92	77.1	1.1%	2.2%	33.7%	50.0%	13.0%
FRC	2.49	0.83	94	81.7	1.1%	4.3%	33.0%	48.9%	20.2%
sGaw	0.29	0.24	59	124.3	23.7%	27.1%	5.1%	20.3%	49.2%
DLCO	12.08	5.00	118	55.7	0.0%	0.0%	83.9%	87.3%	3.4%
KCO	2.97	0.96	118	78.8	2.5%	5.1%	47.5%	55.9%	19.5%
VA	4.16	1.17	118	71.8	0.0%	0.0%	63.6%	75.4%	4.2%
DACO	12.08	5.00	118	62.5	0.0%	0.8%	72.0%	81.4%	4.2%
DACOpp/FVCpp	0.82	0.30	118	-	-	-	-	-	-
DACOpp/TLCpp	0.86	0.29	90	-	-	-	-	-	-
TLCpp/FVCpp	0.96	0.15	90	-	-	-	-	-	-
central convexity	30.47	19.18	141	-	-	-	-	-	-
peripheral convexity	57.58	19.76	182	-	-	-	-	-	-

Table S5. Parameters from first spirometry studies of <10 pack-year smoking.

first spiro <10 py	-	-	-	-	>5%	>10%	<5%	<10%	-
	Mean	SD	n	% pred	ULN	ULN	LLN	LLN	≥100%
FEVPEF	0.62	0.33	76	-	-	-	-	-	-
FEV05	1.71	0.52	93	86.3	5.4%	7.5%	21.5%	30.1%	25.8%
FEV1	2.04	0.64	93	80.3	3.2%	4.3%	33.3%	44.1%	20.4%
FEV3	2.37	0.82	92	81.0	2.2%	4.3%	38.0%	50.0%	25.0%
FEV6	2.52	1.32	71	79.9	4.2%	4.2%	39.4%	50.7%	21.1%
FVC	2.53	0.91	93	75.5	1.1%	1.1%	47.3%	60.2%	17.2%
FEF25-75	2.32	1.05	93	115.1	11.8%	19.4%	9.7%	17.2%	57.0%
FEF50	3.20	1.32	93	-	-	-	-	-	-
FEF75	0.99	0.79	93	182.8	14.0%	23.7%	0.0%	7.5%	75.3%
PEF	6.14	2.14	93	94.2	7.5%	17.2%	9.7%	14.0%	38.7%
PIF	3.57	1.39	93	-	-	-	-	-	-
FET	7.26	3.10	93	-	-	-	-	-	-
FEVPEF/FVC %	26.19	8.44	76	-	-	-	-	-	-
FEV05/FVC %	69.20	10.67	93	112.3	32.3%	46.2%	5.4%	6.5%	83.9%
FEV1/FEV3 %	86.91	11.92	92	102.9	20.7%	42.4%	6.5%	6.5%	72.8%
FEV1/FVC %	81.95	8.73	93	106.7	17.2%	33.3%	4.3%	6.5%	79.6%
FEV3/FVC %	94.50	14.05	92	106.0	22.8%	35.9%	3.3%	5.4%	83.7%
FEV6/FVC %	98.11	42.93	71	105.3	9.9%	43.7%	4.2%	7.0%	84.5%
PEF/FEV1	3.05	0.75	93	116.3	31.2%	40.9%	7.5%	7.5%	71.0%
PEF/FVC	2.50	0.65	93	125.6	37.6%	47.3%	5.4%	6.5%	82.8%
FEF25-75/FVC	0.98	0.47	93	154.1	25.8%	43.0%	3.2%	5.4%	77.4%
FEF50/FVC	1.17	0.39	93	-	-	-	-	-	-
FEF75/FVC	0.41	0.29	93	286.5	36.6%	47.3%	0.0%	1.1%	88.2%
FEF25-75/PEF %	40.02	17.07	93	125.7	20.4%	24.7%	5.4%	8.6%	62.4%
FEF50/PEF %	54.83	19.83	93	-	-	-	-	-	-
FEF75/PEF %	17.31	12.26	93	239.7	29.0%	33.3%	0.0%	1.1%	74.2%
PIF/PEF	0.62	0.25	93	-	-	-	-	-	-
TLC	3.90	1.13	42	73.6	0.0%	0.0%	61.9%	69.0%	7.1%
RV	1.44	0.51	41	74.3	0.0%	0.0%	36.6%	43.9%	12.2%
FRC	2.30	0.79	42	80.1	0.0%	2.4%	35.7%	47.6%	23.8%
sGaw	0.30	0.16	24	130.9	37.5%	41.7%	4.2%	20.8%	50.0%
DLCO	11.72	6.93	50	56.3	0.0%	0.0%	86.0%	90.0%	6.0%
KCO	3.07	1.72	50	81.0	4.0%	6.0%	50.0%	58.0%	18.0%
VA	3.93	2.15	50	71.2	0.0%	0.0%	64.0%	74.0%	4.0%
DACO	11.72	6.93	50	63.5	0.0%	2.0%	78.0%	86.0%	8.0%
DACOpp/FVCpp	0.84	0.33	50	-	-	-	-	-	-
DACOpp/TLCpp	0.87	0.29	38	-	-	-	-	-	-
TLCpp/FVCpp	0.96	0.17	38	-	-	-	-	-	-
central convexity	15.29	30.72	68	-	-	-	-	-	-
peripheral convexity	46.12	40.13	86	-	-	-	-	-	-

Table S6. Parameters from first spirometry studies of >20 pack-year smoking.

first spiro >20 py	-	-	-	-	>5%	>10%	<5%	<10%	-
	Mean	SD	n	% pred	ULN	ULN	LLN	LLN	≥100%
FEVPEF	0.68	0.34	61	-	-	-	-	-	-
FEV05	1.90	0.52	72	85.6	2.8%	4.2%	15.3%	29.2%	25.0%
FEV1	2.29	0.60	72	81.6	1.4%	2.8%	26.4%	40.3%	18.1%
FEV3	2.67	0.69	71	82.0	0.0%	0.0%	31.0%	40.8%	21.1%
FEV6	2.88	0.76	58	81.4	0.0%	1.7%	34.5%	44.8%	20.7%
FVC	2.89	0.81	72	77.8	0.0%	1.4%	38.9%	50.0%	13.9%
FEF25-75	2.54	1.14	72	112.3	9.7%	16.7%	6.9%	12.5%	52.8%

(Table) contd.....

first spiro >20 py	-	-	-	-	>5%	>10%	<5%	<10%	-
FEF50	3.52	1.53	72	-	-	-	-	-	-
FEF75	0.90	0.49	72	151.0	12.5%	13.9%	0.0%	2.8%	70.8%
PEF	6.77	2.08	72	93.4	6.9%	12.5%	8.3%	20.8%	40.3%
PIF	4.10	1.37	72	-	-	-	-	-	-
FET	7.82	3.11	72	-	-	-	-	-	-
FEVPEF/FVC %	24.11	9.22	61	-	-	-	-	-	-
FEV05/FVC %	66.56	10.20	72	108.3	25.0%	34.7%	6.9%	8.3%	70.8%
FEV1/FEV3 %	85.39	5.73	71	101.2	15.5%	33.8%	9.9%	12.7%	70.4%
FEV1/FVC %	80.17	7.68	72	105.6	11.1%	27.8%	2.8%	8.3%	76.4%
FEV3/FVC %	93.44	4.49	71	104.8	19.7%	31.0%	0.0%	1.4%	83.1%
FEV6/FVC %	97.34	2.67	58	103.6	5.2%	31.0%	0.0%	5.2%	82.8%
PEF/FEV1	3.01	0.83	72	114.4	30.6%	36.1%	8.3%	12.5%	68.1%
PEF/FVC	2.43	0.74	72	121.5	34.7%	40.3%	6.9%	9.7%	72.2%
FEF25-75/FVC	0.93	0.45	72	146.2	29.2%	38.9%	0.0%	2.8%	69.4%
FEF50/FVC	1.02	0.28	72	-	-	-	-	-	-
FEF75/FVC	0.33	0.19	72	216.1	22.2%	31.9%	0.0%	1.4%	80.6%
FEF25-75/PEF %	39.32	16.87	72	123.9	19.4%	23.6%	2.8%	8.3%	59.7%
FEF50/PEF %	53.83	21.05	72	-	-	-	-	-	-
FEF75/PEF %	14.69	9.53	72	186.0	16.7%	19.4%	0.0%	1.4%	76.4%
PIF/PEF	0.64	0.25	72	-	-	-	-	-	-
TLC	4.70	1.18	35	76.9	0.0%	2.9%	57.1%	68.6%	11.4%
RV	1.56	0.58	35	80.3	2.9%	5.7%	31.4%	60.0%	14.3%
FRC	2.72	0.81	35	85.3	2.9%	8.6%	28.6%	48.6%	20.0%
sGaw	0.28	0.33	23	121.7	13.0%	13.0%	8.7%	21.7%	39.1%
DLCO	11.96	4.81	47	53.3	0.0%	0.0%	83.0%	85.1%	0.0%
KCO	2.75	0.84	47	73.8	0.0%	2.1%	51.1%	59.6%	17.0%
VA	4.41	1.07	47	73.2	0.0%	0.0%	61.7%	74.5%	6.4%
DACO	11.96	4.81	47	59.1	0.0%	0.0%	74.5%	80.9%	0.0%
DACOpp/FVCpp	0.77	0.26	47	-	-	-	-	-	-
DACOpp/TLCpp	0.81	0.28	35	-	-	-	-	-	-
TLCpp/FVCpp	0.96	0.12	35	-	-	-	-	-	-
central convexity	16.67	29.68	51	-	-	-	-	-	-
peripheral convexity	54.52	29.00	67	-	-	-	-	-	-

Table S7. Parameters from all first DLCO studies.

first DLCO all	-	-	-	-	>5%	>10%	<5%	<10%	-
	Mean	SD	n	% pred	ULN	ULN	LLN	LLN	≥100%
FEVPEF	0.67	0.25	151	-	-	-	-	-	-
FEV05	1.88	0.51	182	89.0	4.9%	7.1%	17.0%	26.9%	31.9%
FEV1	2.24	0.61	182	83.3	2.2%	4.4%	26.9%	36.8%	22.0%
FEV3	2.60	0.73	181	83.4	2.2%	2.8%	30.9%	43.6%	23.8%
FEV6	2.78	0.80	149	83.5	3.4%	4.7%	31.5%	43.6%	23.5%
FVC	2.78	0.82	182	78.5	2.2%	2.7%	36.8%	48.9%	16.5%
FEF25-75	2.57	1.08	182	119.5	12.1%	17.6%	4.4%	11.5%	62.6%
FEF50	3.60	1.48	182	-	-	-	-	-	-
FEF75	0.90	0.44	182	157.5	9.9%	17.0%	0.0%	4.9%	73.6%
PEF	6.95	2.17	182	99.9	12.1%	20.3%	4.9%	12.6%	48.4%
PIF	4.19	1.36	182	-	-	-	-	-	-
FET	7.46	2.51	182	-	-	-	-	-	-
FEVPEF/FVC %	25.03	7.89	151	-	-	-	-	-	-
FEV05/FVC %	68.65	9.74	182	111.5	29.1%	46.2%	4.9%	6.0%	81.9%
FEV1/FEV3 %	86.46	6.44	181	102.4	17.7%	38.1%	6.6%	7.7%	75.1%
FEV1/FVC %	81.29	7.62	182	106.6	11.0%	27.5%	3.8%	4.9%	80.2%
FEV3/FVC %	93.83	4.24	181	105.2	16.0%	28.2%	1.1%	2.2%	85.6%

(Table) contd.....

first DLCO all	-	-	-	-	>5%	>10%	<5%	<10%	-
FEV6/FVC %	98.11	2.05	149	104.5	6.7%	45.0%	0.0%	1.3%	85.9%
PEF/FEV1	3.16	0.80	182	120.3	37.9%	46.2%	4.9%	6.6%	75.8%
PEF/FVC	2.58	0.71	182	129.4	44.0%	52.7%	3.8%	5.5%	80.2%
FEF25-75/FVC	0.97	0.42	182	152.4	29.1%	44.0%	1.1%	3.8%	75.8%
FEF50/FVC	1.08	0.32	182	-	-	-	-	-	-
FEF75/FVC	0.34	0.19	182	222.8	24.2%	34.6%	0.0%	0.5%	81.3%
FEF25-75/PEF %	38.64	15.18	182	121.3	13.2%	20.3%	3.8%	9.9%	65.4%
FEF50/PEF %	54.16	21.23	182	-	-	-	-	-	-
FEF75/PEF %	13.78	7.74	182	178.3	12.1%	17.6%	0.5%	2.7%	76.9%
PIF/PEF	0.64	0.23	182	-	-	-	-	-	-
TLC	4.36	1.17	137	75.9	0.0%	0.7%	56.9%	70.1%	7.3%
RV	1.53	0.52	137	77.7	0.7%	2.2%	29.2%	47.4%	14.6%
FRC	2.59	0.83	138	83.8	2.2%	5.8%	30.4%	47.1%	24.6%
sGaw	0.27	0.21	97	118.5	21.6%	26.8%	6.2%	17.5%	48.5%
DLCO	11.82	4.64	182	54.9	0.0%	0.0%	86.3%	89.6%	2.2%
KCO	2.94	0.90	182	77.9	1.6%	3.3%	46.2%	54.4%	17.0%
VA	4.12	1.15	182	72.0	0.5%	0.5%	64.8%	75.3%	4.4%
DACO	11.82	4.64	182	61.6	0.0%	0.5%	76.4%	84.6%	2.7%
DACOpp/FVCpp	0.82	0.28	182	-	-	-	-	-	-
DACOpp/TLCpp	0.85	0.28	138	-	-	-	-	-	-
TLCpp/FVCpp	0.96	0.15	138	-	-	-	-	-	-
central convexity	29.56	18.94	136	-	-	-	-	-	-
peripheral convexity	59.49	18.64	177	-	-	-	-	-	-

Table S8. Parameters from first DLCO studies of <10 pack-year smoking.

first DLCO <10 py	-	-	-	-	>5%	>10%	<5%	<10%	-
	Mean	SD	n	% pred	ULN	ULN	LLN	LLN	≥100%
FEVPEF	0.64	0.32	68	-	-	-	-	-	-
FEV05	1.81	0.49	84	90.0	6.0%	9.5%	17.9%	27.4%	31.0%
FEV1	2.15	0.60	84	83.6	2.4%	4.8%	27.4%	35.7%	22.6%
FEV3	2.47	0.76	83	83.3	3.6%	4.8%	32.5%	48.2%	25.3%
FEV6	2.59	0.82	67	83.4	6.0%	6.0%	35.8%	47.8%	22.4%
FVC	2.62	0.81	84	77.7	2.4%	2.4%	40.5%	54.8%	15.5%
FEF25-75	2.53	0.99	84	122.8	11.9%	20.2%	3.6%	9.5%	66.7%
FEF50	3.55	1.35	84	-	-	-	-	-	-
FEF75	0.92	0.46	84	170.3	11.9%	21.4%	0.0%	6.0%	77.4%
PEF	6.71	2.11	84	100.9	13.1%	22.6%	3.6%	10.7%	50.0%
PIF	4.01	1.49	84	-	-	-	-	-	-
FET	7.12	2.44	84	-	-	-	-	-	-
FEVPEF/FVC %	25.69	7.72	68	-	-	-	-	-	-
FEV05/FVC %	70.4	8.7	84	114.1	29.8%	52.4%	2.4%	2.4%	86.9%
FEV1/FEV3 %	87.6	6.1	83	103.7	20.5%	42.2%	3.6%	4.8%	77.1%
FEV1/FVC %	82.8	11.2	84	107.8	14.3%	29.8%	1.2%	1.2%	82.1%
FEV3/FVC %	94.4	4.4	83	105.7	20.5%	32.5%	2.4%	2.4%	89.2%
FEV6/FVC %	98.3	1.8	67	105.3	9.0%	46.3%	0.0%	0.0%	91.0%
PEF/FEV1	3.18	0.76	84	121.2	36.9%	46.4%	3.6%	3.6%	76.2%
PEF/FVC	2.63	0.64	84	132.0	45.2%	53.6%	2.4%	3.6%	86.9%
FEF25-75/FVC	1.02	0.42	84	158.9	27.4%	48.8%	1.2%	2.4%	79.8%
FEF50/FVC	1.14	0.36	84	-	-	-	-	-	-
FEF75/FVC	0.37	0.21	84	247.3	31.0%	40.5%	0.0%	0.0%	85.7%
FEF25-75/PEF %	39.8	15.4	84	124.3	15.5%	19.0%	6.0%	8.3%	70.2%
FEF50/PEF %	55.92	20.67	84	-	-	-	-	-	-
FEF75/PEF %	14.7	8.2	84	193.6	14.3%	21.4%	0.0%	3.6%	77.4%
PIF/PEF	0.63	0.22	84	-	-	-	-	-	-

(Table) contd.....

first DLCO <10 py	-	-	-	-	>5%	>10%	<5%	<10%	-
TLC	4.05	1.13	63	74.8	0.0%	0.0%	60.3%	69.8%	6.3%
RV	1.46	0.54	63	76.0	0.0%	1.6%	27.0%	42.9%	12.7%
FRC	2.45	0.82	63	83.3	1.6%	4.8%	30.2%	44.4%	27.0%
sGaw	0.30	0.18	44	128.3	31.8%	38.6%	4.5%	15.9%	52.3%
DLCO	11.67	4.59	84	55.9	0.0%	0.0%	86.9%	90.5%	3.6%
KCO	3.08	0.91	84	81.0	2.4%	3.6%	44.0%	52.4%	16.7%
VA	3.87	1.12	84	70.3	0.0%	0.0%	69.0%	78.6%	3.6%
DACO	11.67	4.59	84	63.3	0.0%	1.2%	78.6%	86.9%	4.8%
DACOpp/FVCpp	0.85	0.29	84	-	-	-	-	-	-
DACOpp/TLCpp	0.88	0.26	63	-	-	-	-	-	-
TLCpp/FVCpp	0.96	0.17	63	-	-	-	-	-	-
central convexity	13.13	30.51	59	-	-	-	-	-	-
peripheral convexity	53.83	25.59	81	-	-	-	-	-	-

Table S9. Parameters from first DLCO studies of >20 pack-year smoking.

first DLCO >20 py	-	-	-	-	>5%	>10%	<5%	<10%	-
	Mean	SD	n	%pred	ULN	ULN	LLN	LLN	>100%
FEVPEF	0.71	0.35	61	-	-	-	-	-	-
FEV05	1.95	0.52	26	87.6	4.3%	5.7%	14.3%	27.1%	30.0%
FEV1	2.34	0.60	70	83.3	2.9%	2.9%	25.7%	38.6%	21.4%
FEV3	2.75	0.70	70	83.8	1.4%	1.4%	28.6%	40.0%	22.9%
FEV6	2.96	0.77	60	83.8	1.7%	5.0%	28.3%	40.0%	25.0%
FVC	2.97	0.82	70	80.2	2.9%	4.3%	31.4%	42.9%	17.1%
FEF25-75	2.54	1.20	70	112.2	11.4%	14.3%	5.7%	14.3%	54.3%
FEF50	3.60	1.73	26	-	-	-	-	-	-
FEF75	0.85	0.43	70	140.5	7.1%	14.3%	0.0%	2.9%	67.1%
PEF	6.99	2.07	70	96.0	7.1%	17.1%	7.1%	18.6%	42.9%
PIF	4.28	1.21	70	-	-	-	-	-	-
FET	8.09	2.84	70	-	-	-	-	-	-
FEVPEF/FVC %	24.12	7.99	61	-	-	-	-	-	-
FEV05/FVC %	66.28	9.96	70	107.9	27.1%	34.3%	7.1%	10.0%	71.4%
FEV1/FEV3 %	85.21	5.79	70	101.0	17.1%	34.3%	10.0%	11.4%	65.7%
FEV1/FVC %	79.43	7.58	70	104.8	7.1%	27.1%	5.7%	8.6%	72.9%
FEV3/FVC %	93.01	4.36	70	104.4	14.3%	24.3%	0.0%	2.9%	78.6%
FEV6/FVC %	97.67	2.49	60	103.4	3.3%	40.0%	0.0%	3.3%	78.3%
PEF/FEV1	3.05	0.82	70	116.0	34.3%	40.0%	7.1%	11.4%	72.9%
PEF/FVC	2.44	0.75	70	122.3	38.6%	44.3%	5.7%	8.6%	71.4%
FEF25-75/FVC	0.89	0.43	70	141.0	30.0%	35.7%	1.4%	4.3%	65.7%
FEF50/FVC	1.01	0.26	70	-	-	-	-	-	-
FEF75/FVC	0.30	0.16	70	195.5	18.6%	30.0%	0.0%	1.4%	72.9%
FEF25-75/PEF %	37.46	15.97	70	118.3	12.9%	24.3%	1.4%	12.9%	58.6%
FEF50/PEF %	52.80	22.57	70	-	-	-	-	-	-
FEF75/PEF %	13.00	7.97	70	164.4	10.0%	15.7%	0.0%	1.4%	72.9%
PIF/PEF	0.65	0.24	70	-	-	-	-	-	-
TLC	4.70	1.16	52	77.9	0.0%	1.9%	51.9%	65.4%	11.5%
RV	1.58	0.53	52	79.8	1.9%	3.8%	32.7%	55.8%	15.4%
FRC	2.78	0.81	52	86.0	1.9%	7.7%	30.8%	46.2%	26.9%
sGaw	0.25	0.27	36	108.6	11.1%	11.1%	11.1%	22.2%	38.9%
DLCO	11.58	4.52	70	52.6	0.0%	0.0%	87.1%	88.6%	0.0%
KCO	2.68	0.82	70	72.1	0.0%	1.4%	54.3%	61.4%	12.9%
VA	4.43	1.11	70	75.0	1.4%	1.4%	58.6%	70.0%	7.1%
DACO	11.58	4.52	70	58.0	0.0%	0.0%	80.0%	85.7%	0.0%
DACOpp/FVCpp	0.75	0.25	70	-	-	-	-	-	-
DACOpp/TLCpp	0.78	0.28	52	-	-	-	-	-	-

(Table) contd.....

first DLCO >20 py	-	-	-	-	>5%	>10%	<5%	<10%	-
TLCpp/FVCpp	0.95	0.13	52	-	-	-	-	-	-
central concavity	19.67	28.09	54	-	-	-	-	-	-
peripheral concavity	60.13	23.29	68	-	-	-	-	-	-

Table S10. Parameters from all last spirometry studies.

last spiro all	-	-	-	-	>5%	>10%	<5%	<10%	-
	Mean	SD	n	% pred	ULN	ULN	LLN	LLN	≥100%
FEVPEF	0.57	0.23	188	-	-	-	-	-	-
FEV05	1.58	0.48	194	79.6	3.6%	4.6%	28.9%	45.4%	19.6%
FEV1	1.88	0.59	194	74.4	0.5%	1.5%	46.4%	55.2%	11.9%
FEV3	2.19	0.72	185	73.4	0.0%	1.6%	49.2%	60.0%	13.5%
FEV6	2.43	0.78	146	75.4	0.7%	2.1%	47.9%	59.6%	16.4%
FVC	2.33	0.80	194	69.4	1.0%	1.0%	56.7%	70.1%	10.8%
FEF25-75	2.24	1.03	194	115.6	9.8%	15.5%	7.7%	13.4%	56.7%
FEF50	3.17	1.39	194	-	-	-	-	-	-
FEF75	0.82	0.53	194	164.9	12.4%	18.6%	2.6%	5.7%	70.1%
PEF	6.21	2.12	194	95.5	12.9%	20.6%	10.3%	18.0%	40.7%
PIF	3.67	1.40	194	-	-	-	-	-	-
FET	7.11	2.53	194	-	-	-	-	-	-
FEVPEF/FVC %	25.39	8.13	188	-	-	-	-	-	-
FEV05/FVC %	69.44	10.43	194	113.4	39.2%	47.9%	3.1%	5.2%	80.4%
FEV1/FEV3 %	86.98	6.28	185	103.3	22.2%	37.8%	5.9%	8.1%	75.7%
FEV1/FVC %	81.88	8.05	194	107.3	18.0%	31.4%	2.1%	4.1%	81.4%
FEV3/FVC %	93.32	4.24	185	105.4	16.8%	29.7%	0.5%	2.2%	85.9%
FEV6/FVC %	97.47	3.18	146	104.5	6.2%	42.5%	2.7%	4.1%	84.2%
PEF/FEV1	3.38	0.92	194	127.5	44.3%	54.6%	3.6%	5.7%	78.4%
PEF/FVC	2.78	0.85	194	139.3	50.0%	57.2%	2.1%	5.7%	81.4%
FEF25-75/FVC	1.04	0.55	194	169.5	35.6%	42.8%	2.6%	5.2%	79.4%
FEF50/FVC	1.12	0.41	194	-	-	-	-	-	-
FEF75/FVC	0.39	0.30	194	310.2	33.0%	43.8%	0.5%	0.5%	89.7%
FEF25-75/PEF %	37.68	16.07	194	123.4	14.9%	21.6%	5.2%	10.8%	63.4%
FEF50/PEF %	53.47	20.80	194	-	-	-	-	-	-
FEF75/PEF %	14.52	10.40	194	251.0	19.6%	24.2%	1.0%	2.6%	78.9%
PIF/PEF	0.62	0.21	194	-	-	-	-	-	-
TLC	3.71	1.11	132	65.3	0.0%	0.0%	78.8%	84.8%	3.8%
RV	1.36	0.55	131	66.3	0.0%	0.0%	47.3%	61.1%	9.9%
FRC	2.39	0.80	132	76.4	0.0%	0.8%	45.5%	59.1%	18.2%
sGaw	0.34	0.41	107	145.9	27.1%	29.9%	9.3%	20.6%	54.2%
DLCO	7.64	3.47	165	36.1	0.0%	0.0%	98.8%	99.4%	0.0%
KCO	2.21	0.84	165	59.7	0.0%	0.6%	76.4%	84.8%	4.2%
VA	3.54	1.02	165	61.6	0.0%	0.0%	84.8%	87.9%	1.2%
DACO	7.64	3.47	165	42.6	0.0%	0.0%	95.2%	97.6%	0.0%
DACOpp/FVCpp	0.63	0.26	165	-	-	-	-	-	-
DACOpp/TLCpp	0.67	0.27	123	-	-	-	-	-	-
TLCpp/FVCpp	0.92	0.17	123	-	-	-	-	-	-
central convexity	32.55	20.36	147	-	-	-	-	-	-
peripheral convexity	62.91	18.88	182	-	-	-	-	-	-

Table S11. Parameters from last spirometry studies of <10 pack-year smoking.

last spiro <10 py	-	-	-	-	>5%	>10%	<5%	<10%	-
	Mean	SD	n	% pred	ULN	ULN	LLN	LLN	≥100%
FEVPEF	0.55	0.24	90	-	-	-	-	-	-
FEV05	1.49	0.47	93	78.6	3.2%	4.3%	29.0%	45.2%	19.4%
FEV1	1.75	0.56	93	72.1	0.0%	0.0%	49.5%	57.0%	6.5%
FEV3	2.01	0.67	90	71.0	0.0%	1.1%	53.3%	63.3%	8.9%
FEV6	2.22	0.74	69	73.0	1.4%	1.4%	50.7%	63.8%	10.1%
FVC	2.13	0.75	93	66.2	0.0%	0.0%	58.1%	76.3%	5.4%
FEF25-75	2.19	1.02	93	117.9	10.8%	16.1%	8.6%	16.1%	60.2%
FEF50	3.05	1.24	93	-	-	-	-	-	-
FEF75	0.87	0.64	93	181.4	17.2%	22.6%	2.2%	8.6%	68.8%
PEF	5.93	2.13	93	95.4	12.9%	17.2%	8.6%	14.0%	38.7%
PIF	3.47	1.43	93	-	-	-	-	-	-
FET	6.91	2.48	93	-	-	-	-	-	-
FEVPEF/FVC %	26.75	8.03	90	-	-	-	-	-	-
FEV05/FVC %	71.12	9.57	93	115.9	40.9%	53.8%	1.1%	2.2%	84.9%
FEV1/FEV3 %	87.98	6.20	90	104.4	22.2%	42.2%	2.2%	4.4%	80.0%
FEV1/FVC %	83.14	7.64	93	108.5	19.4%	32.3%	1.1%	3.2%	87.1%
FEV3/FVC %	93.87	4.02	90	106.0	21.1%	33.3%	0.0%	0.0%	86.7%
FEV6/FVC %	97.43	3.77	69	105.0	5.8%	44.9%	4.3%	5.8%	85.5%
PEF/FEV1	3.43	0.83	93	129.5	48.4%	60.2%	2.2%	3.2%	84.9%
PEF/FVC	2.84	0.74	93	142.9	54.8%	65.6%	1.1%	4.3%	90.3%
FEF25-75/FVC	1.10	0.57	93	178.5	36.6%	44.1%	2.2%	4.3%	83.9%
FEF50/FVC	1.20	0.46	93	-	-	-	-	-	-
FEF75/FVC	0.45	0.37	93	380.4	39.8%	51.6%	1.1%	1.1%	91.4%
FEF25-75/PEF %	39.01	17.70	93	127.5	16.1%	22.6%	6.5%	9.7%	63.4%
FEF50/PEF %	54.21	20.39	93	-	-	-	-	-	-
FEF75/PEF %	16.38	13.21	93	323.8	26.9%	32.3%	1.1%	1.1%	81.7%
PIF/PEF	0.61	0.22	93	-	-	-	-	-	-
TLC	3.42	1.11	60	63.2	0.0%	0.0%	83.3%	88.3%	5.0%
RV	1.35	0.61	59	67.0	0.0%	0.0%	47.5%	55.9%	13.6%
FRC	2.28	0.83	60	75.9	0.0%	0.0%	43.3%	56.7%	20.0%
sGaw	0.39	0.53	50	168.5	34.0%	36.0%	4.0%	18.0%	56.0%
DLCO	7.74	3.64	73	37.5	0.0%	0.0%	98.6%	100.0%	0.0%
KCO	2.41	0.91	73	64.7	0.0%	1.4%	67.1%	76.7%	8.2%
VA	3.29	0.98	73	59.0	0.0%	0.0%	89.0%	91.8%	0.0%
DACO	7.74	3.64	73	44.8	0.0%	0.0%	91.8%	95.9%	0.0%
DACOpp/FVCpp	0.68	0.28	8	-	-	-	-	-	-
DACOpp/TLCpp	0.73	0.29	7	-	-	-	-	-	-
TLCpp/FVCpp	0.93	0.19	7	-	-	-	-	-	-
central convexity	21.89	25.44	75	-	-	-	-	-	-
peripheral convexity	52.36	38.41	82	-	-	-	-	-	-

Table S12. Parameters from last spirometry studies of >20 pack-year smoking.

last spiro >20 py	-	-	-	-	>5%	>10%	<5%	<10%	-
	Mean	SD	n	% pred	ULN	ULN	LLN	LLN	≥100%
FEVPEF	0.60	0.25	71	-	-	-	-	-	-
FEV05	1.69	0.47	73	80.9	1.4%	2.7%	24.7%	39.7%	19.2%
FEV1	2.04	0.60	73	77.4	1.4%	2.7%	39.7%	52.1%	16.4%
FEV3	2.42	0.71	69	76.9	0.0%	1.4%	42.0%	56.5%	18.8%
FEV6	2.64	0.81	56	77.9	0.0%	3.6%	44.6%	55.4%	23.2%
FVC	2.57	0.83	73	73.7	2.7%	2.7%	53.4%	63.0%	16.4%
FEF25-75	2.29	1.07	73	113.2	9.6%	15.1%	6.8%	11.0%	52.1%

(Table) contd.....

last spiro >20 py	-	-	-	-	>5%	>10%	<5%	<10%	-
FEF50	3.33	1.55	73	-	-	-	-	-	-
FEF75	0.81	0.44	73	152.8	11.0%	16.4%	1.4%	1.4%	68.5%
PEF	6.32	2.14	73	92.3	9.6%	20.5%	15.1%	24.7%	37.0%
PIF	3.84	1.35	73	-	-	-	-	-	-
FET	7.23	2.56	73	-	-	-	-	-	-
FEVPEF/FVC %	24.04	8.56	71	-	-	-	-	-	-
FEV05/FVC %	67.4	11.2	73	110.3	37.0%	38.4%	5.5%	8.2%	72.6%
FEV1/FEV3 %	85.4	6.4	69	101.5	23.2%	31.9%	10.1%	13.0%	66.7%
FEV1/FVC %	80.4	8.9	73	105.9	19.2%	32.9%	4.1%	6.8%	75.3%
FEV3/FVC %	92.9	4.5	69	104.9	14.5%	29.0%	1.4%	4.3%	85.5%
FEV6/FVC %	97.5	2.7	56	104.1	5.4%	41.1%	1.8%	3.6%	83.9%
PEF/FEV1	3.16	0.87	73	119.0	39.7%	45.2%	6.8%	11.0%	68.5%
PEF/FVC	2.56	0.85	73	128.4	43.8%	49.3%	4.1%	9.6%	65.8%
FEF25-75/FVC	0.97	0.56	73	159.6	34.2%	37.0%	2.7%	5.5%	72.6%
FEF50/FVC	1.03	0.32	73	-	-	-	-	-	-
FEF75/FVC	0.34	0.21	73	251.3	26.0%	35.6%	0.0%	0.0%	87.7%
FEF25-75/PEF %	37.5	14.6	73	122.9	16.4%	21.9%	2.7%	11.0%	65.8%
FEF50/PEF %	54.32	20.91	73	-	-	-	-	-	-
FEF75/PEF %	13.5	6.6	73	191.8	15.1%	20.5%	0.0%	1.4%	79.5%
PIF/PEF	0.64	0.21	73	-	-	-	-	-	-
TLC	4.14	1.01	50	70.1	0.0%	0.0%	72.0%	78.0%	4.0%
RV	1.43	0.52	50	69.7	0.0%	0.0%	44.0%	62.0%	6.0%
FRC	2.56	0.71	50	79.8	0.0%	0.0%	40.0%	58.0%	20.0%
sGaw	0.29	0.30	38	126.1	18.4%	21.1%	18.4%	28.9%	50.0%
DLCO	7.48	3.39	66	34.8	0.0%	0.0%	98.5%	98.5%	0.0%
KCO	1.99	0.76	66	54.4	0.0%	0.0%	84.8%	89.4%	1.5%
VA	3.82	1.02	66	65.0	0.0%	0.0%	80.3%	81.8%	1.5%
DACO	7.48	3.39	66	40.3	0.0%	0.0%	97.0%	98.5%	0.0%
DACOpp/FVCpp	0.57	0.25	66	-	-	-	-	-	-
DACOpp/TLCpp	0.60	0.26	49	-	-	-	-	-	-
TLCpp/FVCpp	0.91	0.15	49	-	-	-	-	-	-
central convexity	18.97	27.08	49	-	-	-	-	-	-
peripheral convexity	59.37	19.79	72	-	-	-	-	-	-

Table S13. Parameters from all last DLCO studies.

last DLCO all	-	-	-	-	>5%	>10%	<5%	<10%	-
	Mean	SD	n	% pred	ULN	ULN	LLN	LLN	≥100%
FEVPEF	0.59	0.22	176	-	-	-	-	-	-
FEV05	1.63	0.46	182	81.0	3.8%	4.9%	26.4%	42.9%	19.8%
FEV1	1.94	0.57	182	75.9	1.1%	2.2%	43.4%	52.7%	12.1%
FEV3	2.26	0.68	175	74.8	0.6%	2.3%	47.4%	58.3%	14.3%
FEV6	2.46	0.76	144	75.8	1.4%	2.8%	47.9%	59.7%	16.7%
FVC	2.40	0.77	182	71.1	1.1%	1.1%	55.5%	69.2%	11.0%
FEF25-75	2.26	1.00	182	114.7	8.2%	13.7%	7.7%	12.1%	58.8%
FEF50	3.21	1.36	182	-	-	-	-	-	-
FEF75	0.81	0.49	182	156.6	8.8%	15.4%	2.7%	4.9%	71.4%
PEF	6.40	2.04	182	97.1	14.3%	22.0%	9.9%	18.1%	43.4%
PIF	3.85	1.33	182	-	-	-	-	-	-
FET	7.35	2.36	182	-	-	-	-	-	-
FEVPEF/FVC %	25.48	7.44	176	-	-	-	-	-	-
FEV05/FVC %	69.16	9.97	182	112.9	38.5%	47.3%	3.3%	4.9%	81.3%
FEV1/FEV3 %	86.66	5.97	175	102.9	20.6%	38.9%	6.3%	8.0%	77.1%
FEV1/FVC %	81.58	7.95	182	106.9	16.5%	30.2%	2.7%	5.5%	82.4%
FEV3/FVC %	93.46	4.18	175	105.4	16.0%	30.3%	0.6%	2.3%	86.9%
FEV6/FVC %	97.26	6.46	144	104.6	5.6%	43.8%	1.4%	2.8%	85.4%
PEF/FEV1	3.38	0.92	182	127.8	44.5%	54.4%	3.8%	4.9%	79.7%

(Table) contd.....

last DLCO all	-	-	-	-	>5%	>10%	<5%	<10%	-
PEF/FVC	2.77	0.84	182	139.0	50.0%	57.7%	2.7%	5.5%	81.3%
FEF25-75/FVC	1.00	0.47	182	161.7	34.6%	42.9%	2.2%	4.9%	79.1%
FEF50/FVC	1.09	0.36	182	-	-	-	-	-	-
FEF75/FVC	0.36	0.23	182	260.5	31.3%	42.9%	0.5%	0.5%	88.5%
FEF25-75/PEF %	36.61	14.72	182	119.0	11.5%	19.2%	5.5%	10.4%	63.7%
FEF50/PEF %	52.12	19.97	182	-	-	-	-	-	-
FEF75/PEF %	13.41	8.33	182	193.1	15.4%	19.2%	1.1%	2.2%	79.1%
PIF/PEF	0.63	0.21	182	-	-	-	-	-	-
TLC	3.77	1.05	130	66.1	0.0%	0.0%	77.7%	85.4%	3.8%
RV	1.36	0.56	130	67.5	0.0%	0.8%	47.7%	61.5%	10.0%
FRC	2.43	0.77	130	77.4	0.0%	0.8%	44.6%	59.2%	16.9%
sGaw	0.34	0.40	109	145.2	28.4%	31.2%	7.3%	18.3%	56.9%
DLCO	7.77	3.46	182	37.1	0.0%	0.0%	97.8%	99.5%	0.0%
KCO	2.27	0.88	182	61.2	0.0%	0.5%	73.6%	81.9%	5.5%
VA	3.50	1.00	182	61.7	0.0%	0.0%	84.6%	87.9%	1.1%
DACO	7.77	3.46	182	43.7	0.0%	0.0%	94.5%	97.3%	0.0%
DACOpp/FVCpp	0.65	0.28	182	-	-	-	-	-	-
DACOpp/TLCpp	0.68	0.27	130	-	-	-	-	-	-
TLCpp/FVCpp	0.92	0.18	130	-	-	-	-	-	-
central convexity	32.65	20.24	145	-	-	-	-	-	-
peripheral convexity	63.01	18.75	176	-	-	-	-	-	-

Table S14. Parameters from last DLCO studies of <10 pack-year smoking.

last DLCO <10 py		-	-	-	>5%	>10%	<5%	<10%	-
	Mean	SD	n	%pred	ULN	ULN	LLN	LLN	>100%
FEVPEF	0.59	0.21	81	-	-	-	-	-	-
FEV05	1.55	0.45	84	80.8	3.6%	4.8%	25.0%	41.7%	19.0%
FEV1	1.83	0.54	84	74.3	1.2%	1.2%	44.0%	54.8%	6.0%
FEV3	2.09	0.64	82	73.1	1.2%	2.4%	51.2%	62.2%	9.8%
FEV6	2.29	0.69	66	74.3	3.0%	3.0%	50.0%	63.6%	10.6%
FVC	2.23	0.72	84	68.4	0.0%	0.0%	57.1%	76.2%	6.0%
FEF25-75	2.20	0.92	84	116.8	8.3%	14.3%	8.3%	14.3%	63.1%
FEF50	3.11	1.18	84	-	-	-	-	-	-
FEF75	0.82	0.55	84	165.2	10.7%	16.7%	2.4%	7.1%	69.0%
PEF	6.16	2.03	84	97.6	15.5%	19.0%	8.3%	14.3%	42.9%
PIF	3.72	1.33	84	-	-	-	-	-	-
FET	7.20	2.22	84	-	-	-	-	-	-
FEVPEF/FVC %	27.10	7.10	81	-	-	-	-	-	-
FEV05/FVC %	70.96	8.85	84	115.6	40.5%	53.6%	1.2%	2.4%	85.7%
FEV1/FEV3 %	87.59	5.28	82	103.9	19.5%	43.9%	3.7%	4.9%	82.9%
FEV1/FVC %	82.85	7.31	84	108.1	15.5%	29.8%	1.2%	4.8%	88.1%
FEV3/FVC %	94.21	3.65	82	106.2	19.5%	34.1%	0.0%	0.0%	90.2%
FEV6/FVC %	98.11	1.81	66	105.4	6.1%	47.0%	0.0%	1.5%	89.4%
PEF/FEV1	3.44	0.85	84	129.9	47.6%	59.5%	2.4%	2.4%	86.9%
PEF/FVC	2.83	0.75	84	142.6	54.8%	66.7%	2.4%	3.6%	89.3%
FEF25-75/FVC	1.04	0.45	84	167.5	34.5%	44.0%	1.2%	3.6%	83.3%
FEF50/FVC	1.16	0.40	84	-	-	-	-	-	-
FEF75/FVC	0.39	0.27	84	288.9	35.7%	48.8%	1.2%	1.2%	89.3%
FEF25-75/PEF %	37.70	15.73	84	121.8	11.9%	20.2%	7.1%	7.1%	64.3%
FEF50/PEF %	52.88	19.22	84	-	-	-	-	-	-
FEF75/PEF %	14.29	10.14	84	210.9	20.2%	25.0%	1.2%	1.2%	81.0%
PIF/PEF	0.63	0.21	84	-	-	-	-	-	-
TLC	3.52	1.03	58	64.4	0.0%	0.0%	82.8%	89.7%	5.2%
RV	1.34	0.62	58	67.8	0.0%	1.7%	50.0%	58.6%	13.8%
FRC	2.32	0.80	58	76.9	0.0%	0.0%	44.8%	60.3%	17.2%
sGaw	0.38	0.51	51	165.6	35.3%	39.2%	0.0%	13.7%	60.8%

(Table) contd.....

last DLCO <10 py		-	-	-	>5%	>10%	<5%	<10%	-
DLCO	7.87	3.56	84	38.7	0.0%	0.0%	97.6%	100.0%	0.0%
KCO	2.49	0.94	84	66.7	0.0%	1.2%	63.1%	72.6%	9.5%
VA	3.25	0.95	84	59.3	0.0%	0.0%	88.1%	91.7%	0.0%
DACO	7.87	3.56	84	46.2	0.0%	0.0%	91.7%	96.4%	0.0%
DACOpp/FVCpp	0.70	0.29	84	-	-	-	-	-	-
DACOpp/TLCpp	0.74	0.29	58	-	-	-	-	-	-
TLCpp/FVCpp	0.94	0.21	58	-	-	-	-	-	-
central concavity	23.64	19.39	72	-	-	-	-	-	-
peripheral concavity	58.57	20.37	79	-	-	-	-	-	-

Table S15. Parameters from last DLCO studies of >20 pack-year smoking.

last DLCO >20 py	-	-	-	-	>5%	>10%	<5%	<10%	-
	Mean	SD	n	% pred	ULN	ULN	LLN	LLN	≥100%
FEVPEF	0.61	0.23	68	-	-	-	-	-	-
FEV05	1.72	0.46	70	81.5	1.4%	2.9%	24.3%	38.6%	20.0%
FEV1	2.07	0.58	70	77.9	1.4%	2.9%	40.0%	50.0%	17.1%
FEV3	2.48	0.69	66	77.7	0.0%	1.5%	40.9%	54.5%	19.7%
FEV6	2.67	0.79	55	78.7	0.0%	3.6%	43.6%	54.5%	23.6%
FVC	2.63	0.80	70	74.5	2.9%	2.9%	52.9%	62.9%	15.7%
FEF25-75	2.33	1.12	70	112.4	8.6%	12.9%	7.1%	10.0%	52.9%
FEF50	3.34	1.55	70	-	-	-	-	-	-
FEF75	0.82	0.45	70	149.2	8.6%	14.3%	1.4%	1.4%	70.0%
PEF	6.47	2.09	70	93.5	10.0%	22.9%	14.3%	24.3%	38.6%
PIF	3.95	1.35	70	-	-	-	-	-	-
FET	7.40	2.51	70	-	-	-	-	-	-
FEVPEF/FVC %	23.85	7.75	68	-	-	-	-	-	-
FEV05/FVC %	67.1	11.0	70	109.9	35.7%	37.1%	5.7%	7.1%	74.3%
FEV1/FEV3 %	85.1	6.7	66	101.0	21.2%	31.8%	9.1%	12.1%	66.7%
FEV1/FVC %	80.0	9.0	70	105.5	18.6%	32.9%	5.7%	8.6%	77.1%
FEV3/FVC %	92.9	4.6	66	104.7	13.6%	28.8%	1.5%	4.5%	83.3%
FEV6/FVC %	97.4	2.7	55	104.0	3.6%	41.8%	1.8%	3.6%	81.8%
PEF/FEV1	3.19	0.9	70	120.3	41.4%	45.7%	7.1%	10.0%	70.0%
PEF/FVC	2.58	0.9	70	129.2	44.3%	50.0%	4.3%	10.0%	67.1%
FEF25-75/FVC	0.95	0.5	70	155.4	34.3%	37.1%	2.9%	5.7%	72.9%
FEF50/FVC	1.01	0.30	70	-	-	-	-	-	-
FEF75/FVC	0.33	0.20	70	236.6	25.7%	35.7%	0.0%	0.0%	87.1%
FEF25-75/PEF %	36.6	14.4	70	119.7	14.3%	20.0%	2.9%	12.9%	64.3%
FEF50/PEF %	52.98	20.82	70	-	-	-	-	-	-
FEF75/PEF %	13.1	6.5	70	182.1	12.9%	17.1%	0.0%	1.4%	80.0%
PIF/PEF	0.64	0.21	70	-	-	-	-	-	-
TLC	4.15	1.00	50	70.7	0.0%	0.0%	70.0%	78.0%	4.0%
RV	1.44	0.51	50	71.7	0.0%	0.0%	42.0%	60.0%	6.0%
FRC	2.58	0.70	50	80.9	0.0%	0.0%	38.0%	56.0%	20.0%
sGaw	0.29	0.29	39	125.7	17.9%	20.5%	17.9%	28.2%	51.3%
DLCO	7.61	3.38	70	35.3	0.0%	0.0%	98.6%	98.6%	0.0%
KCO	2.01	0.75	70	55.0	0.0%	0.0%	84.3%	88.6%	1.4%
VA	3.83	1.00	70	65.2	0.0%	0.0%	80.0%	81.4%	1.4%
DACO	7.61	3.38	70	40.9	0.0%	0.0%	97.1%	98.6%	0.0%
DACOpp/FVCpp	0.58	0.26	70	-	-	-	-	-	-
DACOpp/TLCpp	0.60	0.26	50	-	-	-	-	-	-
TLCpp/FVCpp	0.92	0.15	50	-	-	-	-	-	-
central convexity	20.54	20.11	49	-	-	-	-	-	-
peripheral convexity	60.22	17.85	69	-	-	-	-	-	-

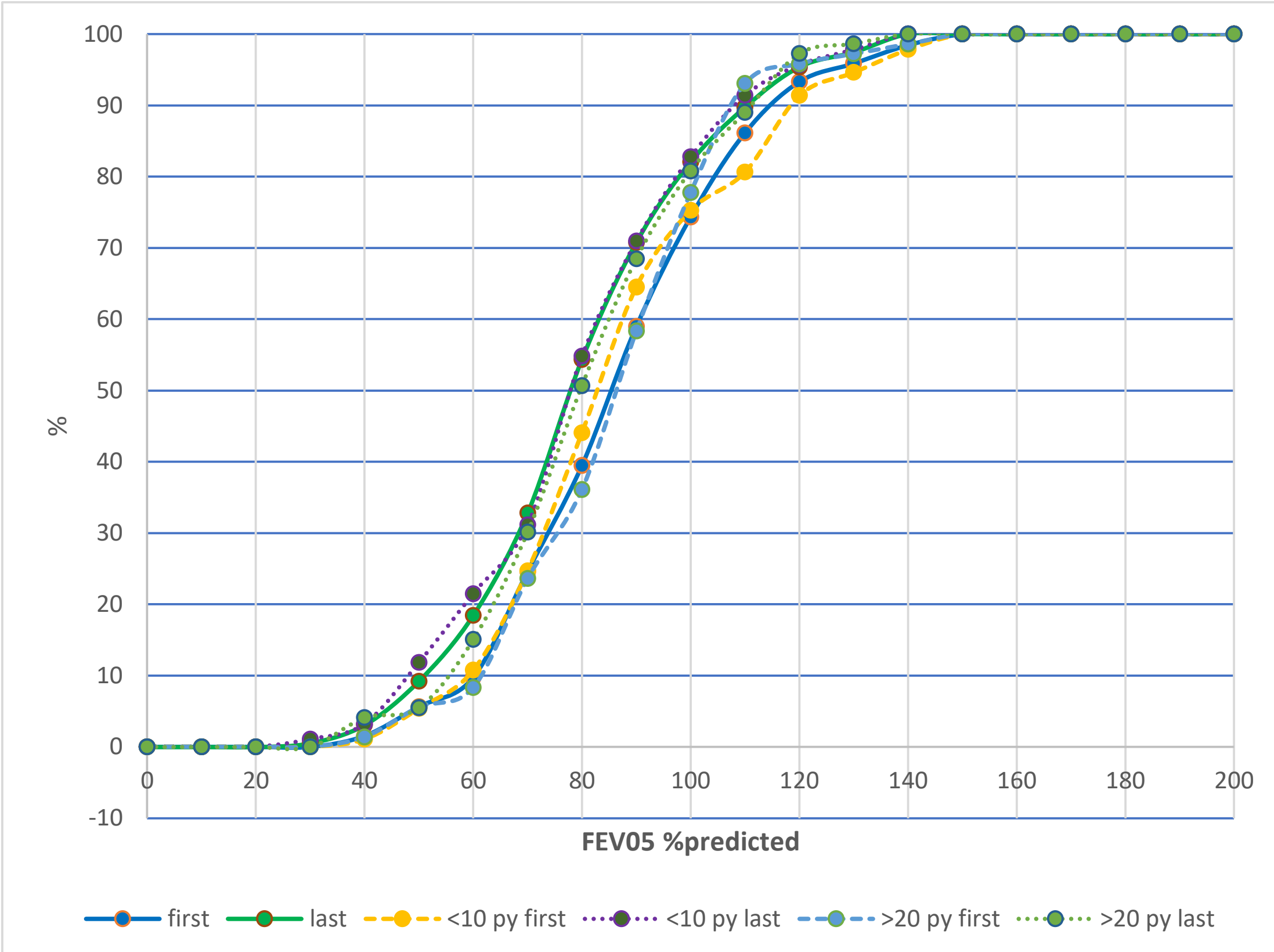

100
90
80
70
60
50
40
30
20
10
0
-10
%
0
20
40
60
80
100
120
140
160
180
200
FEV05 %predicted
first
last
<10 py first
<10 py last
>20 py first
>20 py last

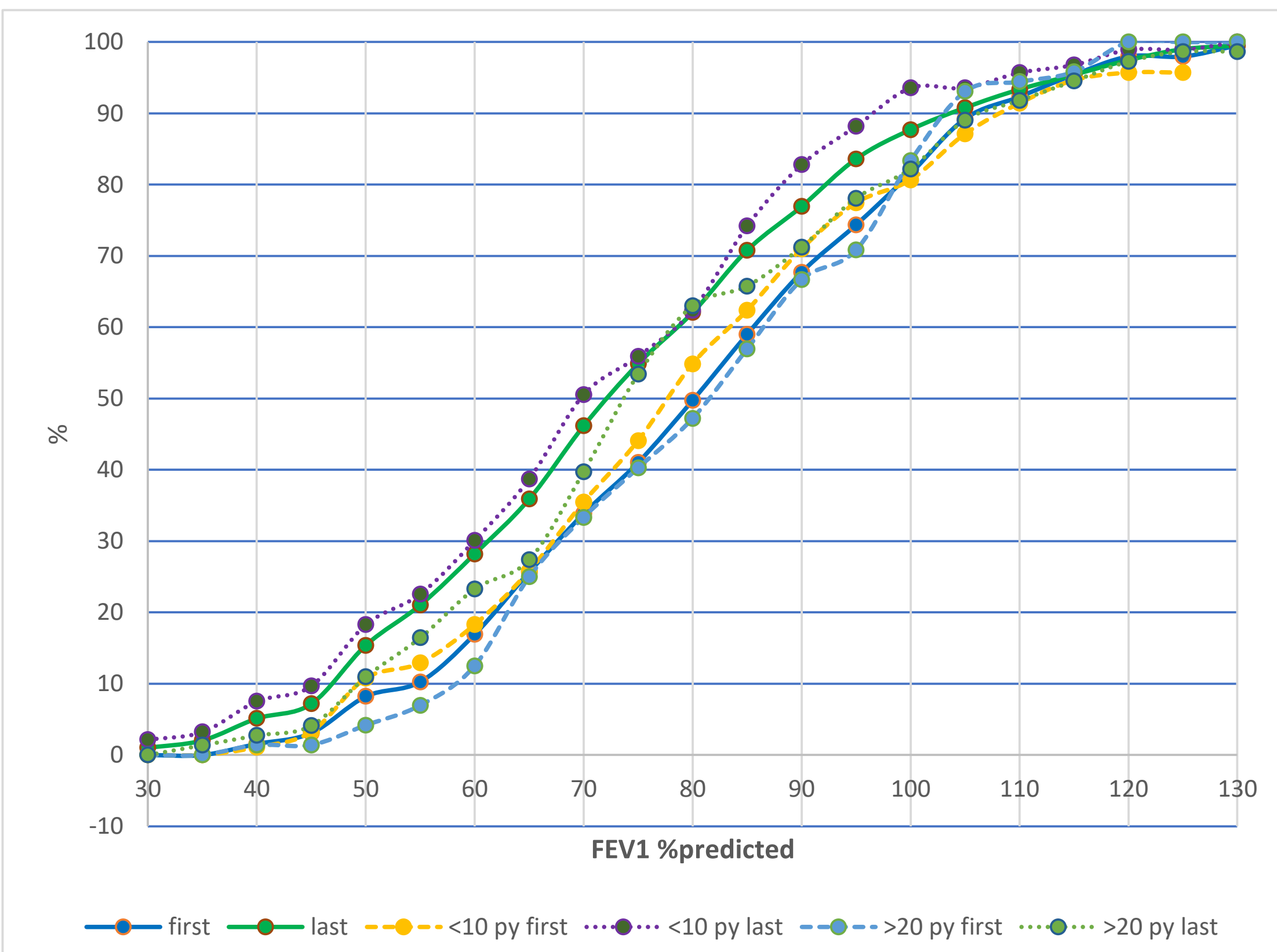

100
90
80
70
60
50
40
30
20
10
0
-10
%
30
40
50
60
70
80
90
100
110
120
130
FEV1 %predicted
first
last
<10 py first
<10 py last
>20 py first
>20 py last

Fig. contd.....

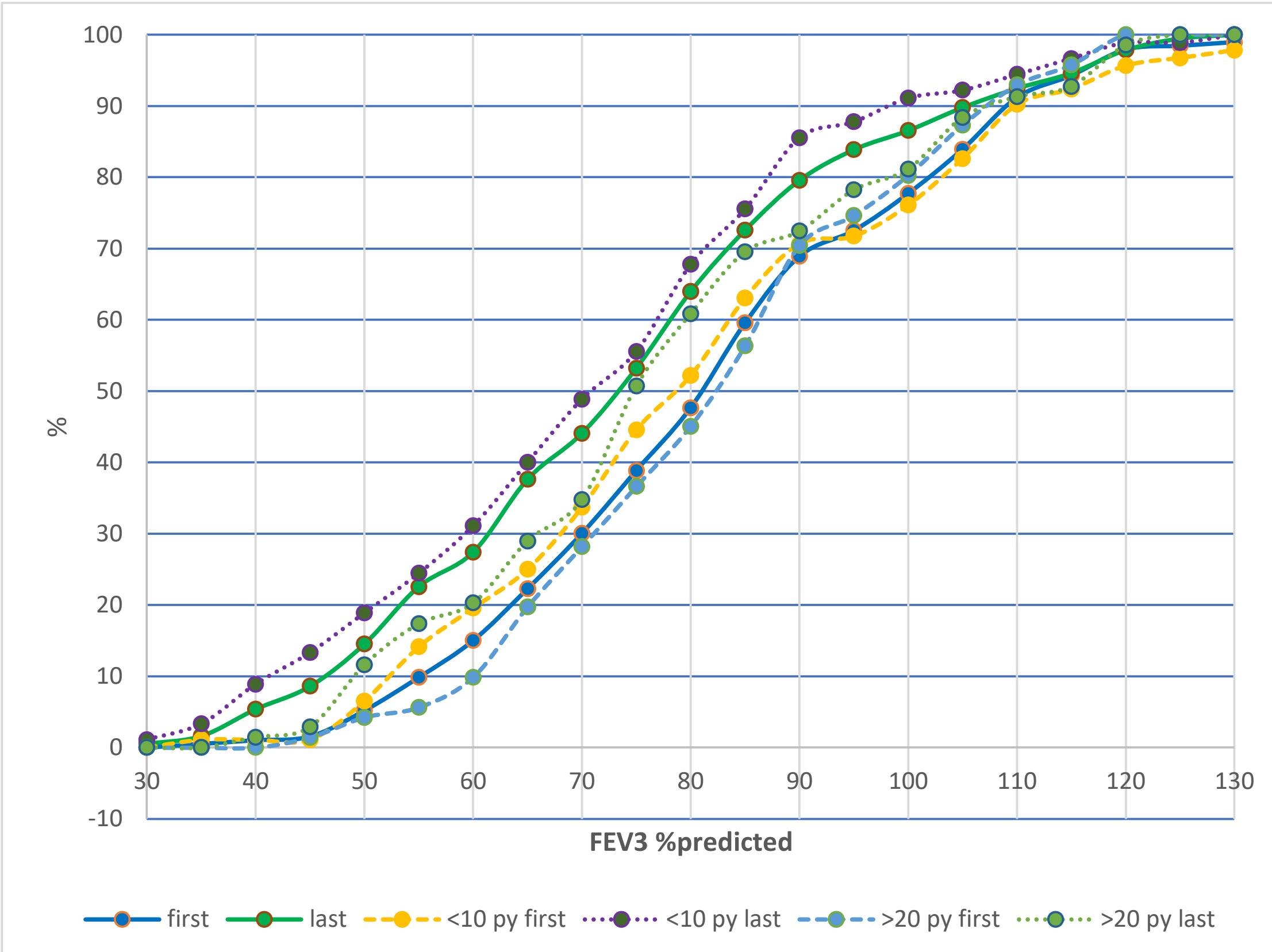

100
90
80
70
60
50
40
30
20
10
0
-10
%
30
40
50
60
70
80
90
100
110
120
130
FEV3 %predicted
first
last
<10 py first
<10 py last
>20 py first
>20 py last

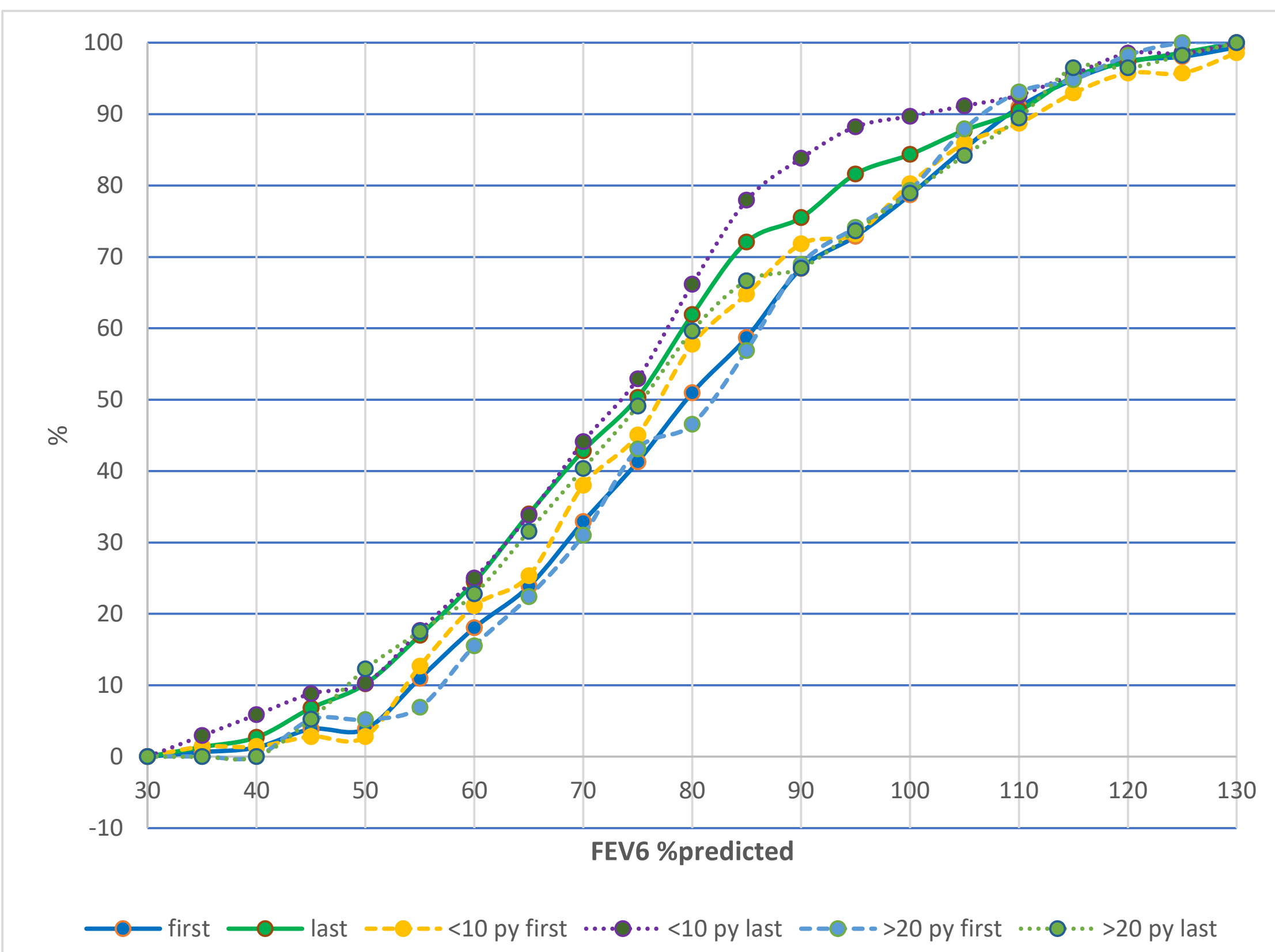

100
90
80
70
60
50
40
30
20
10
0
-10
%
30
40
50
60
70
80
90
100
110
120
130
FEV6 %predicted
first
last
<10 py first
<10 py last
>20 py first
>20 py last

Fig. contd.....

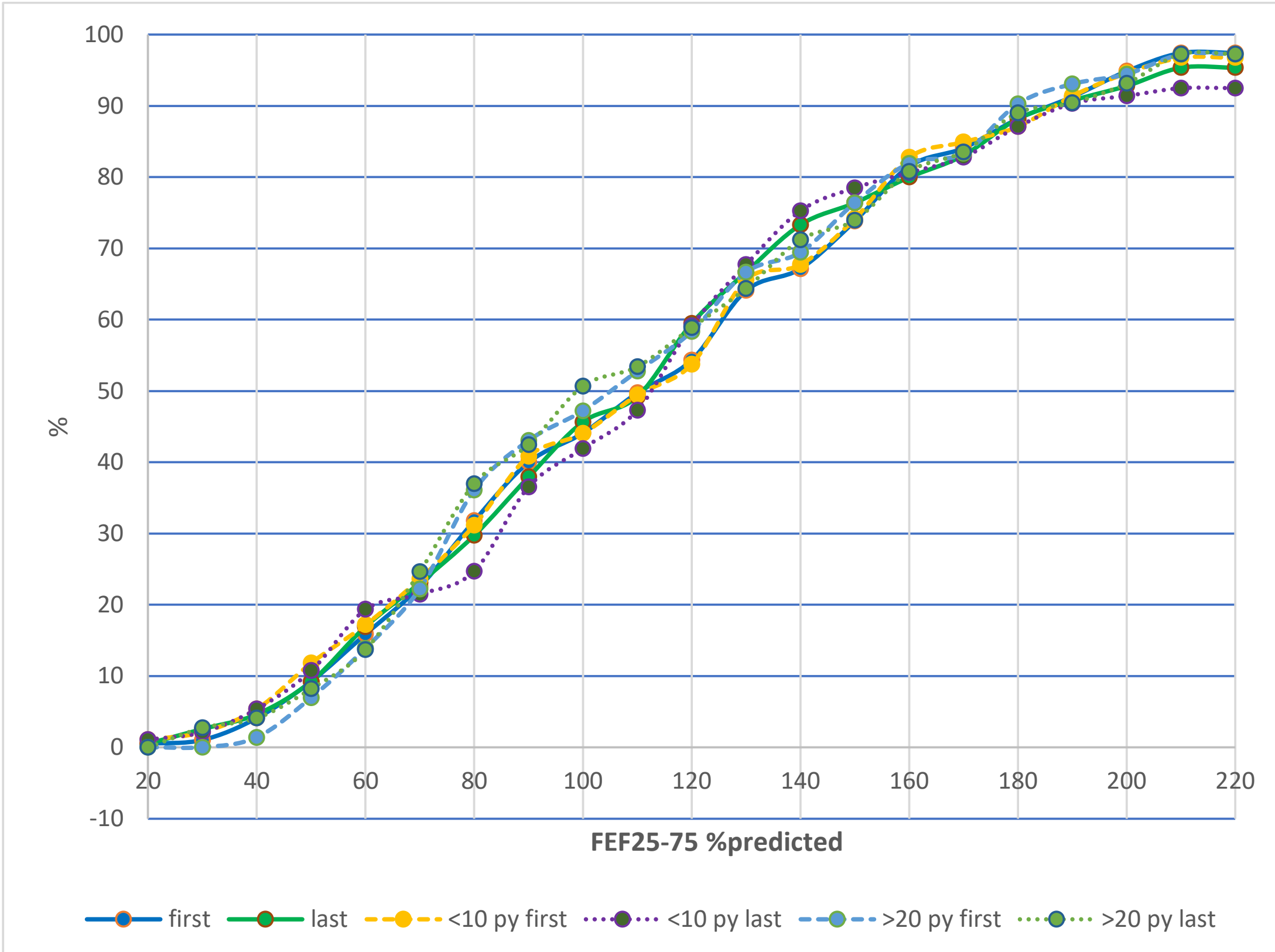
%
FEF25-75 %predicted
first
last
<10 py first
<10 py last
>20 py first
>20 py last

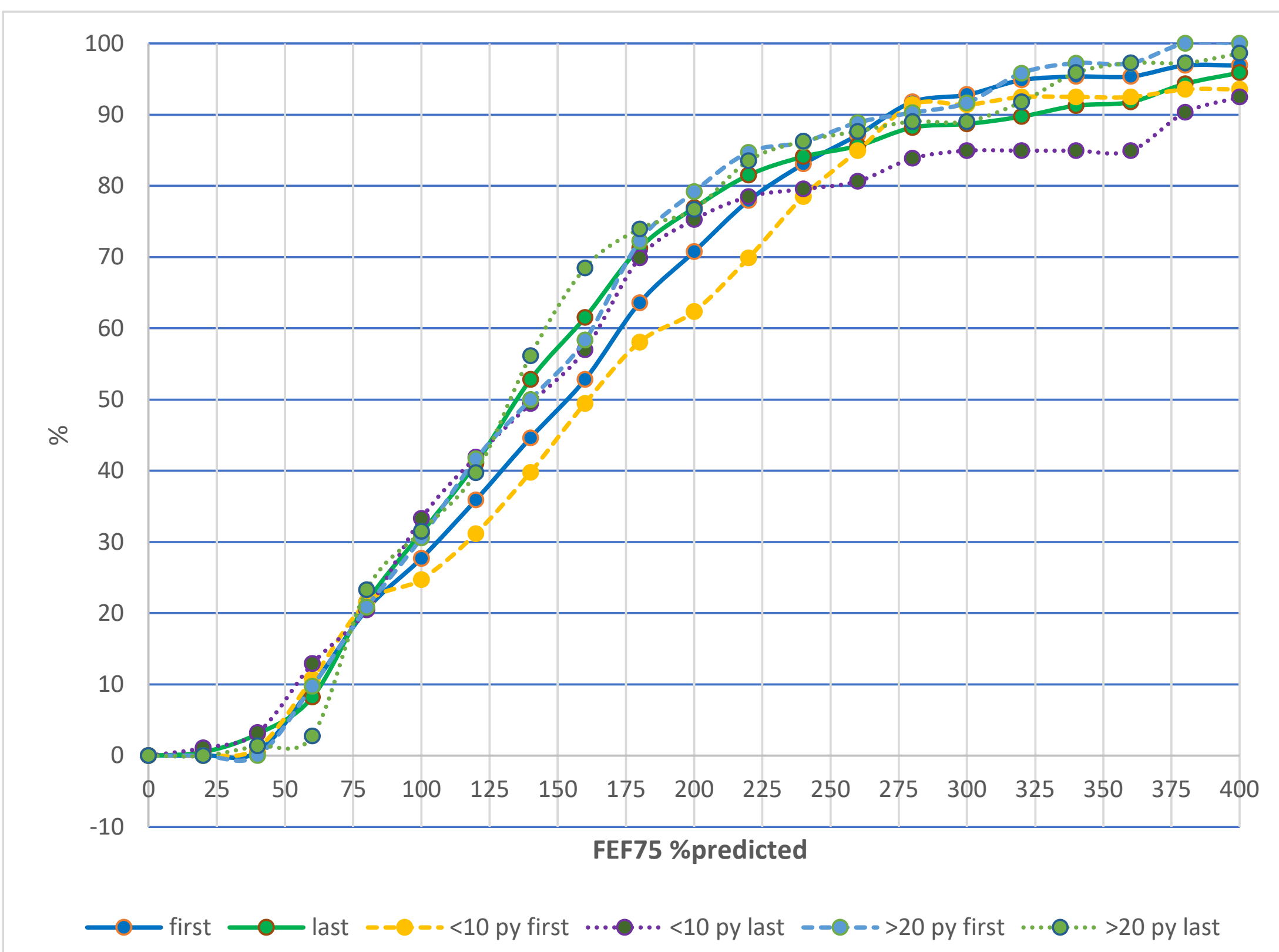
%
FEF75 %predicted
first
last
<10 py first
<10 py last
>20 py first
>20 py last

Fig. contd.....

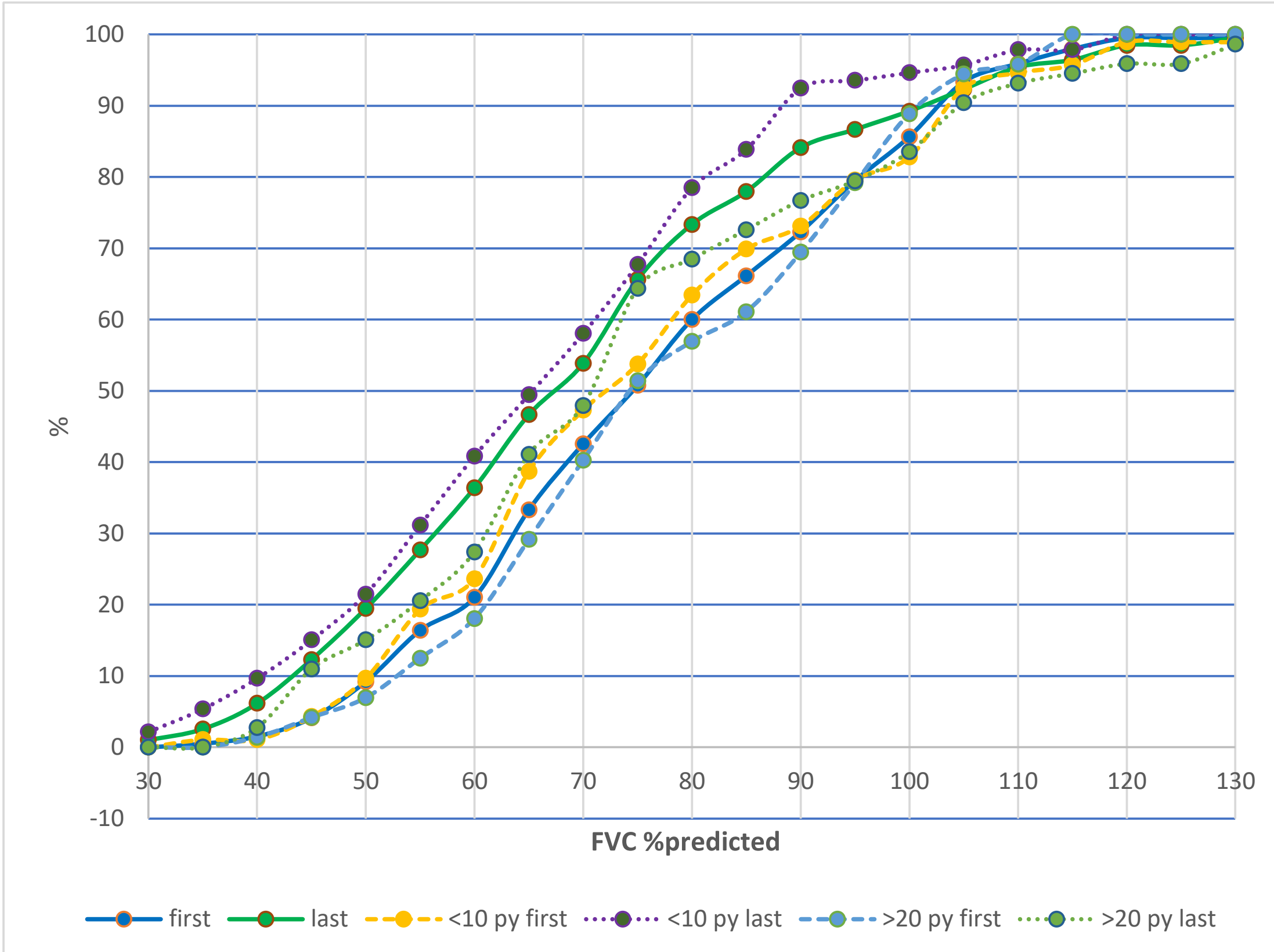
100
90
80
70
60
50
40
30
20
10
0
-10
%
30
40
50
60
70
80
90
100
110
120
130
FVC %predicted
first
last
<10 py first
<10 py last
>20 py first
>20 py last

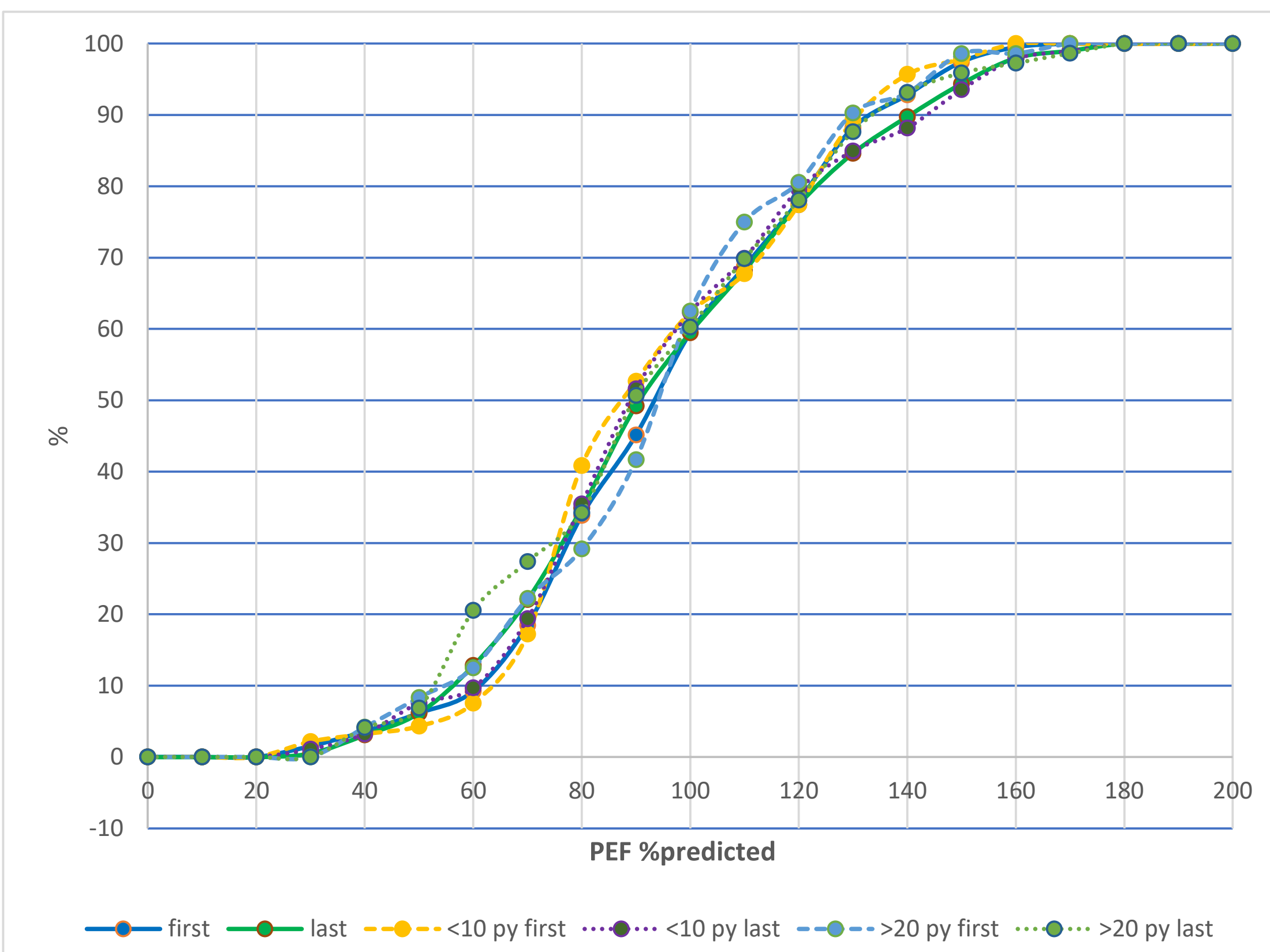
100
90
80
70
60
50
40
30
20
10
0
-10
%
0
20
40
60
80
100
120
140
160
180
200
PEF %predicted
first
last
<10 py first
<10 py last
>20 py first
>20 py last

Fig. contd.....

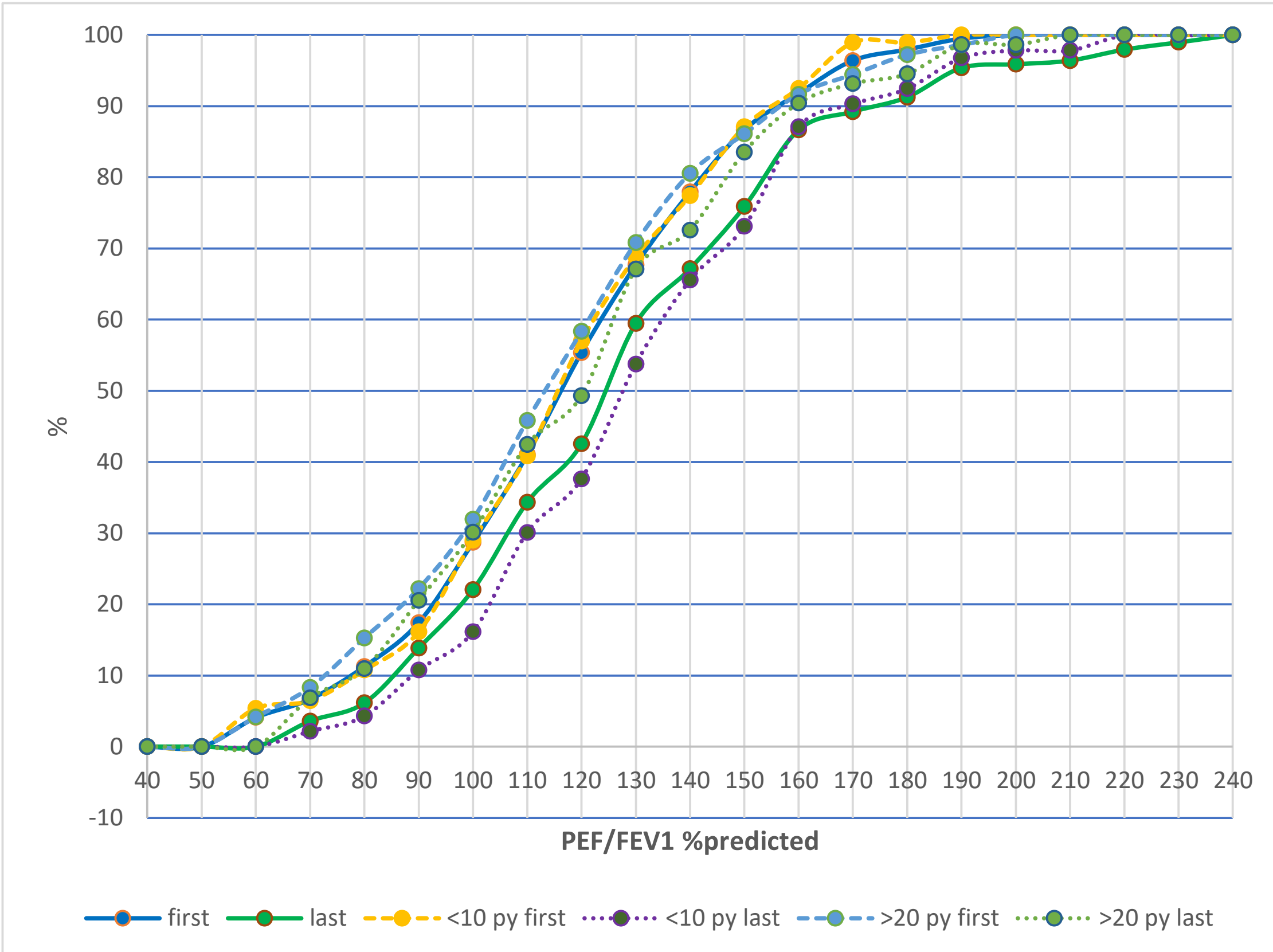
%
PEF/FEV1 %predicted
first
last
<10 py first
<10 py last
>20 py first
>20 py last

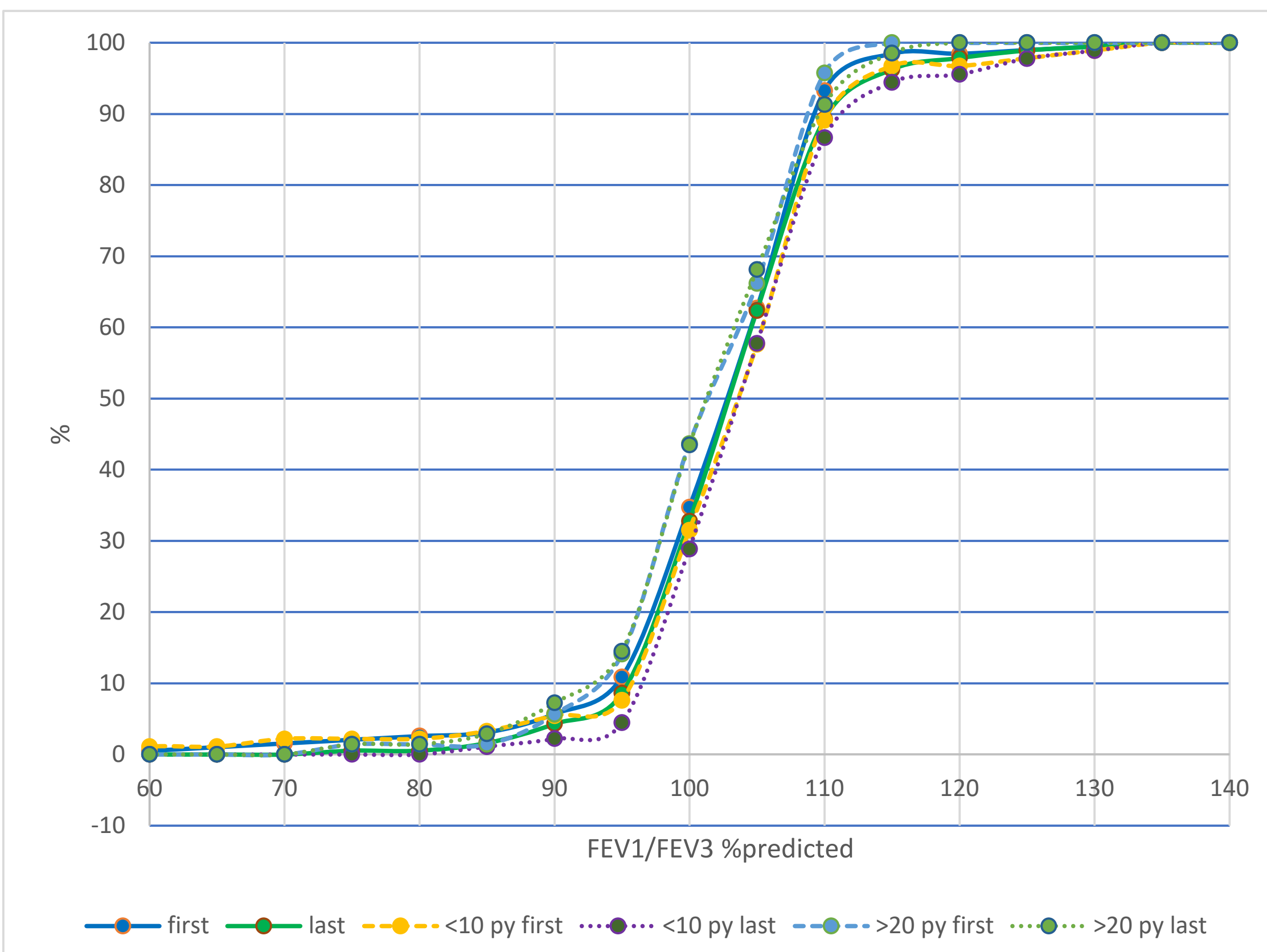
%
FEV1/FEV3 %predicted
first
last
<10 py first
<10 py last
>20 py first
>20 py last

Fig. contd.....

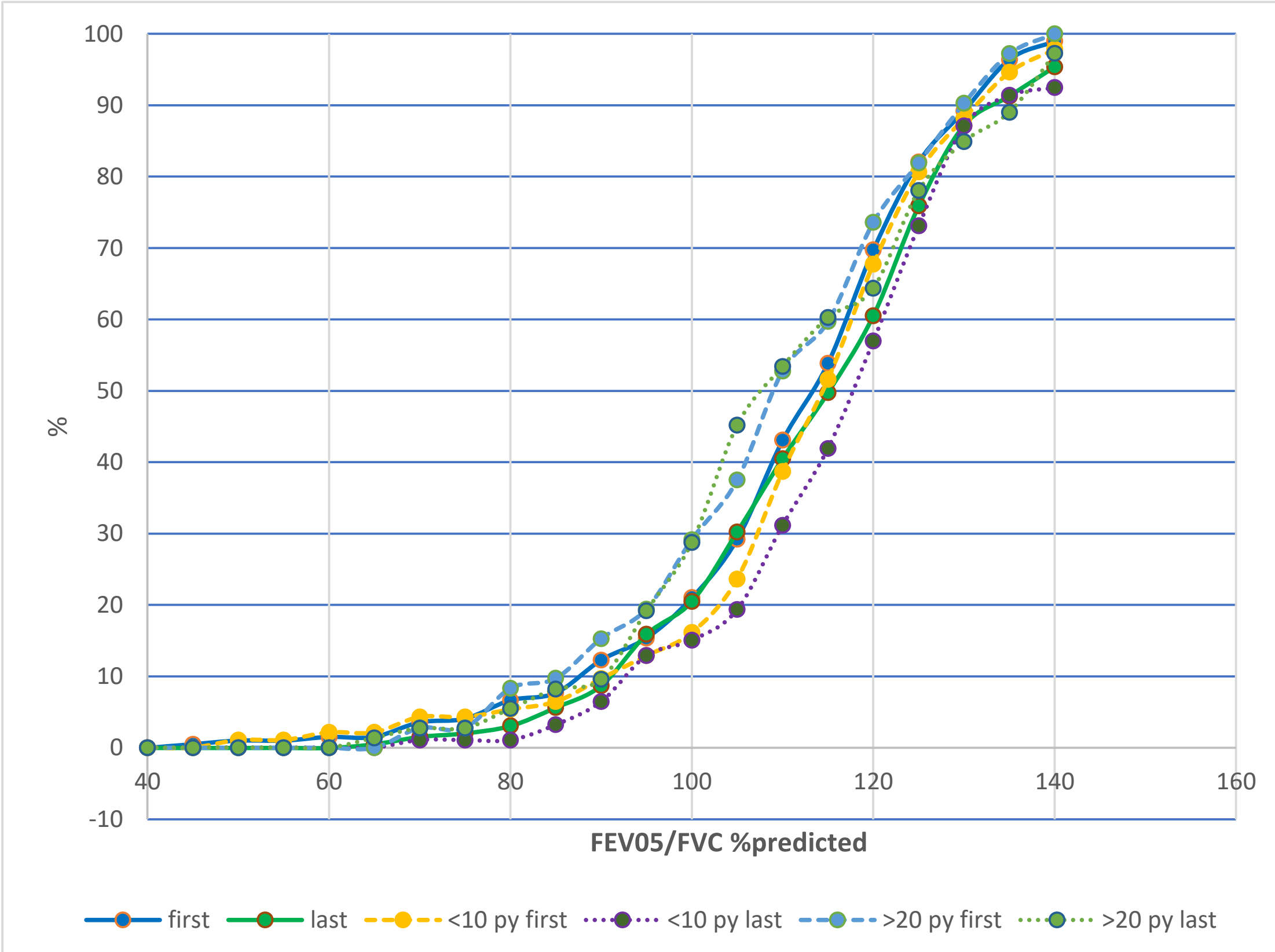
100
90
80
70
60
50
40
30
20
10
0
-10
%
40
60
80
100
120
140
160
FEV05/FVC %predicted
first
last
<10 py first
<10 py last
>20 py first
>20 py last

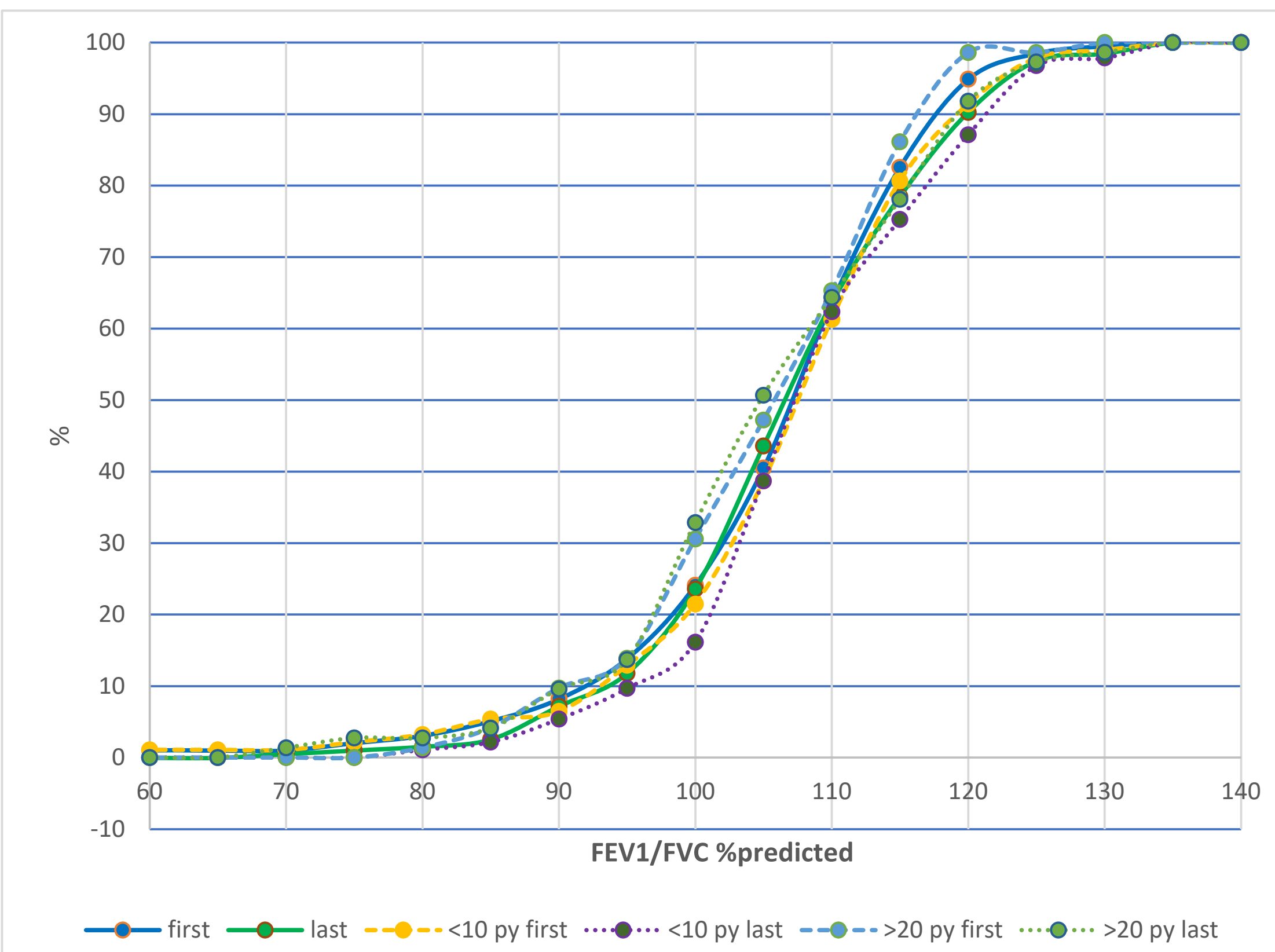
100
90
80
70
60
50
40
30
20
10
0
-10
%
60
70
80
90
100
110
120
130
140
FEV1/FVC %predicted
first
last
<10 py first
<10 py last
>20 py first
>20 py last

Fig. contd.....

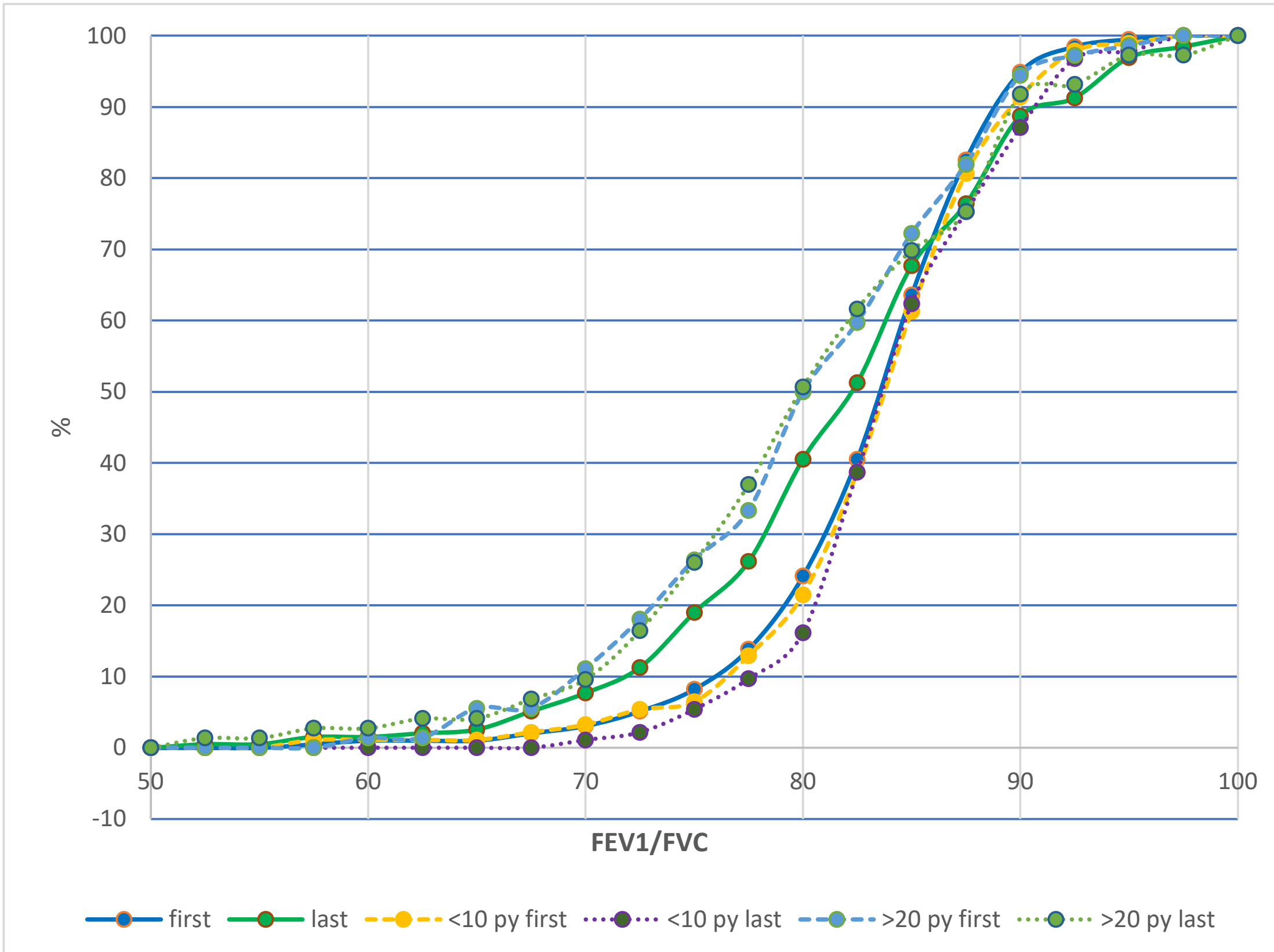

100
90
80
70
60
50
40
30
20
10
0
-10
%
50
60
70
80
90
100
FEV1/FVC
first
last
<10 py first
<10 py last
>20 py first
>20 py last

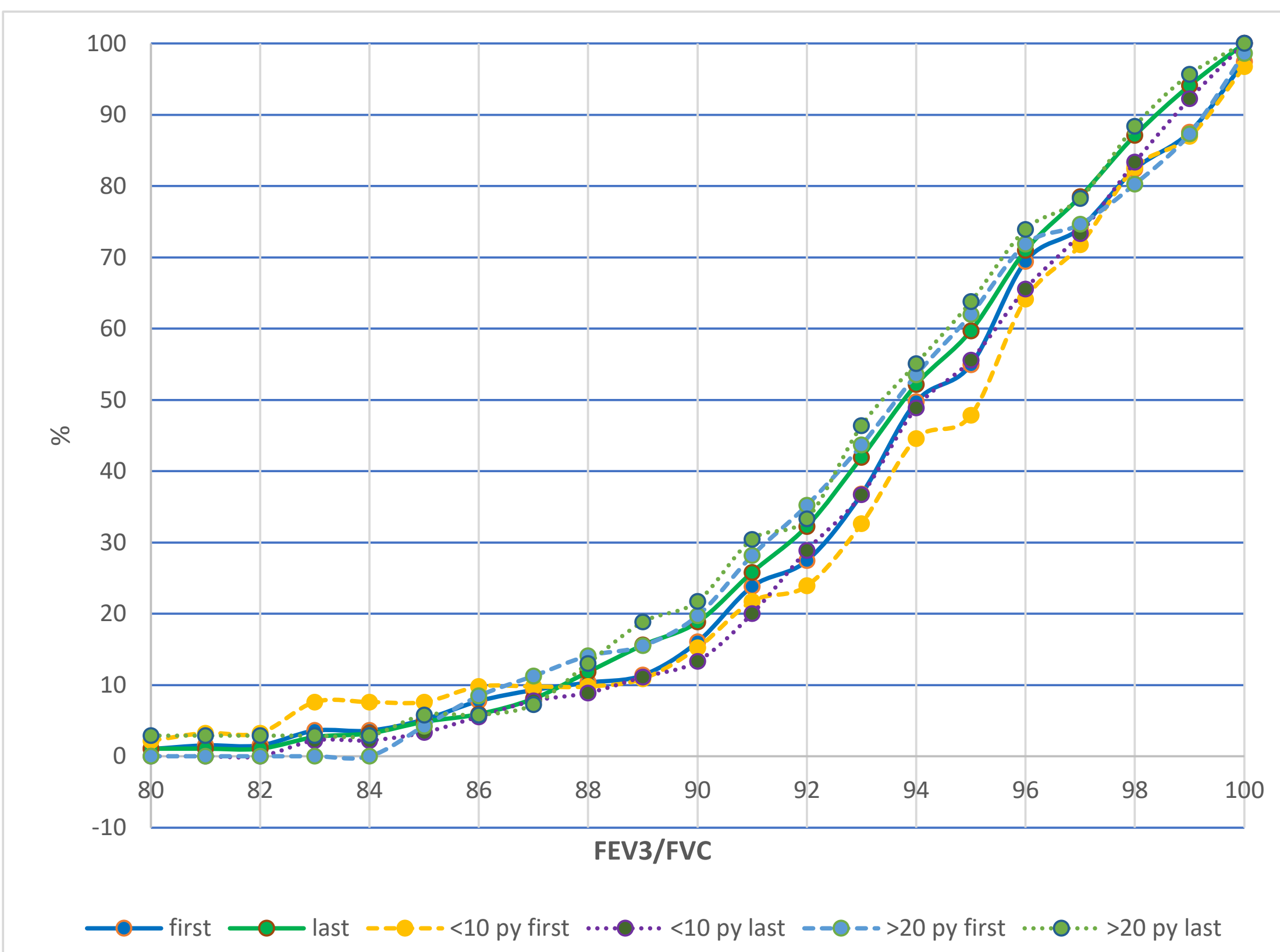

100
90
80
70
60
50
40
30
20
10
0
-10
%
80
82
84
86
88
90
92
94
96
98
100
FEV3/FVC
first
last
<10 py first
<10 py last
>20 py first
>20 py last

Fig. contd.....

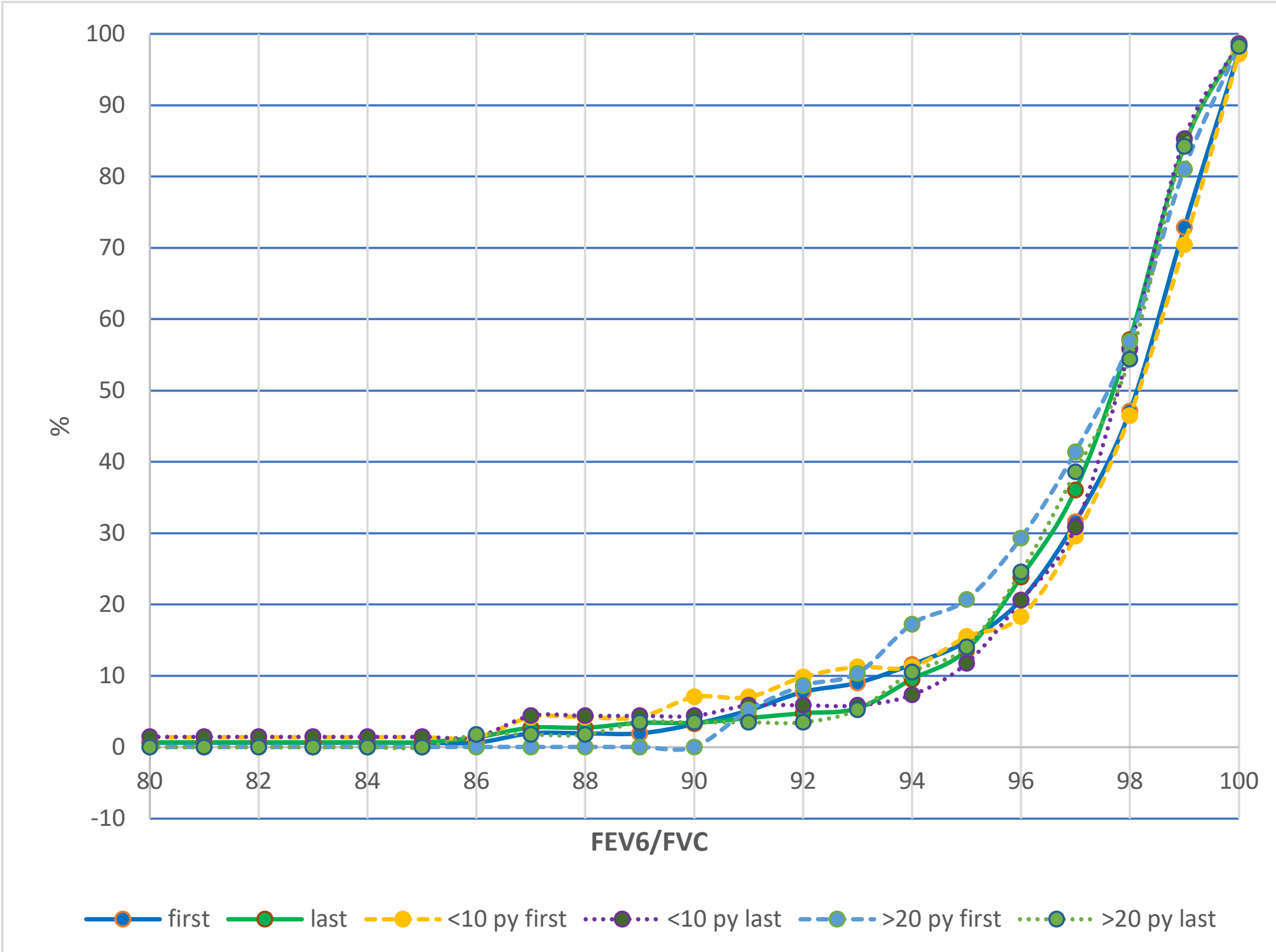

100
90
80
70
60
50
40
30
20
10
0
-10
%
80
82
84
86
88
90
92
94
96
98
100
FEV6/FVC
first
last
<10 py first
<10 py last
>20 py first
>20 py last

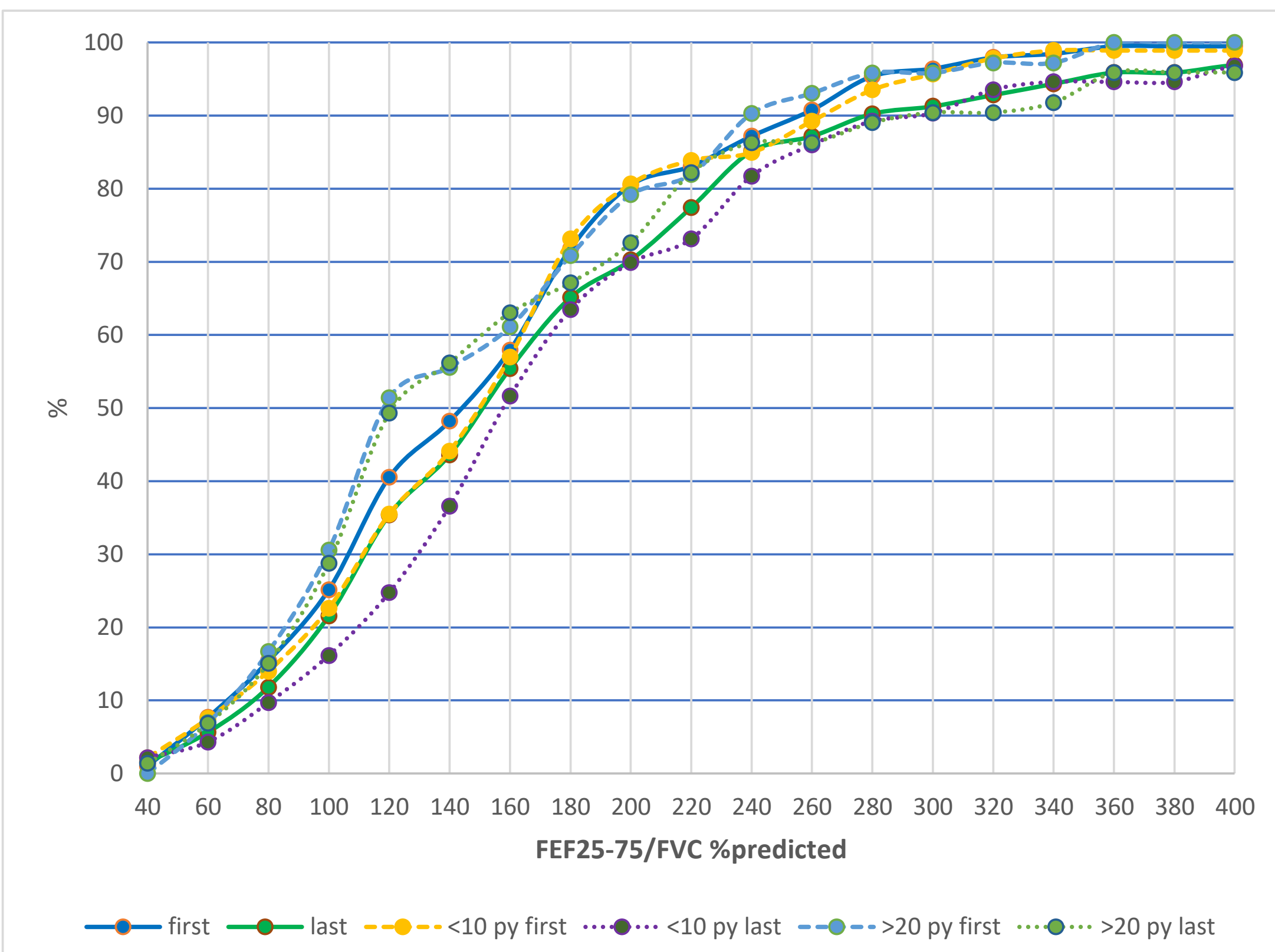

100
90
80
70
60
50
40
30
20
10
0
%
40
60
80
100
120
140
160
180
200
220
240
260
280
300
320
340
360
380
400
FEF25-75/FVC %predicted
first
last
<10 py first
<10 py last
>20 py first
>20 py last

Fig. contd.....

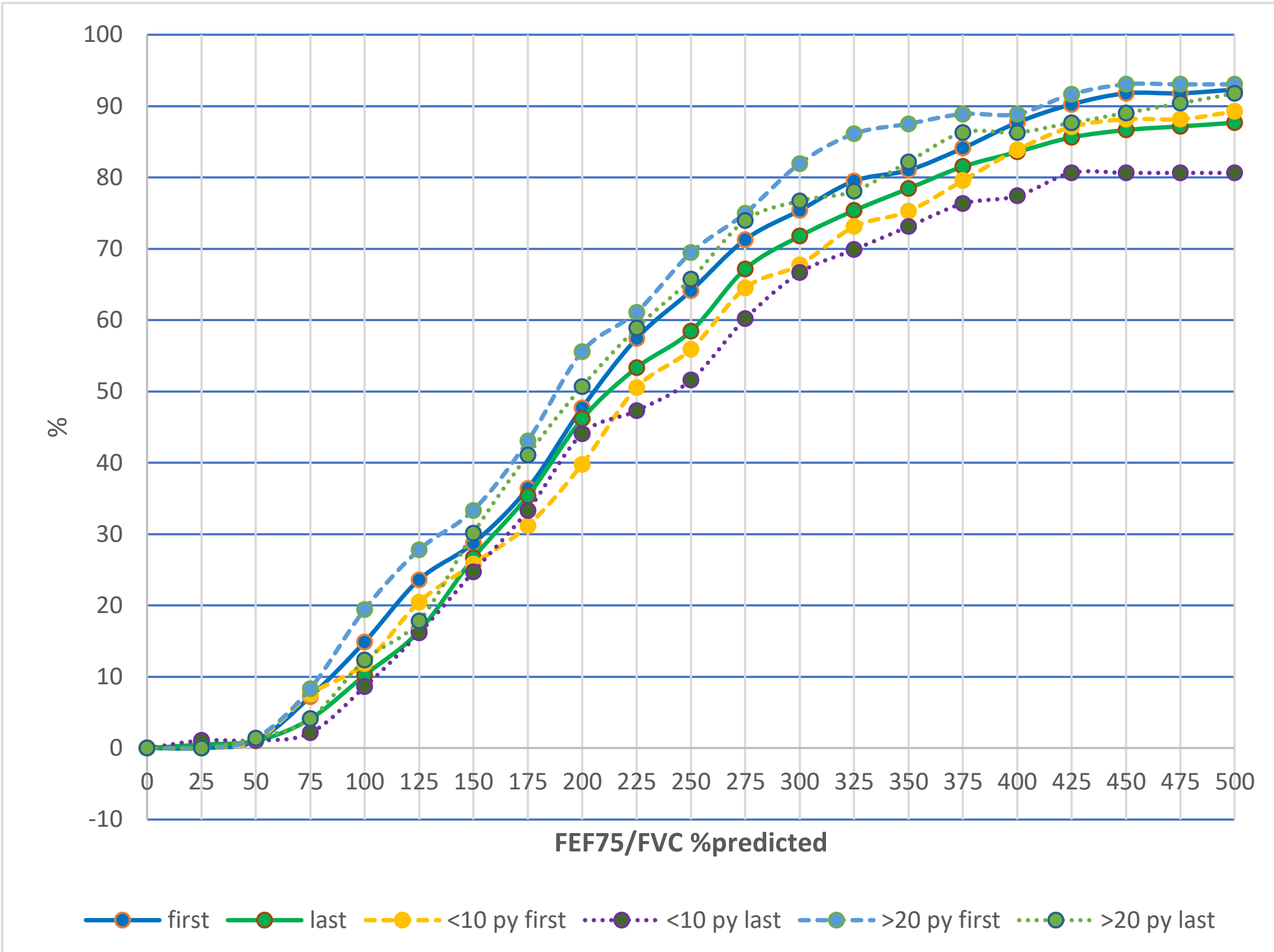
100
90
80
70
60
50
40
30
20
10
0
-10
%
0 25 50 75 100 125 150 175 200 225 250 275 300 325 350 375 400 425 450 475 500
FEF75/FVC %predicted
first
last
<10 py first
<10 py last
>20 py first
>20 py last

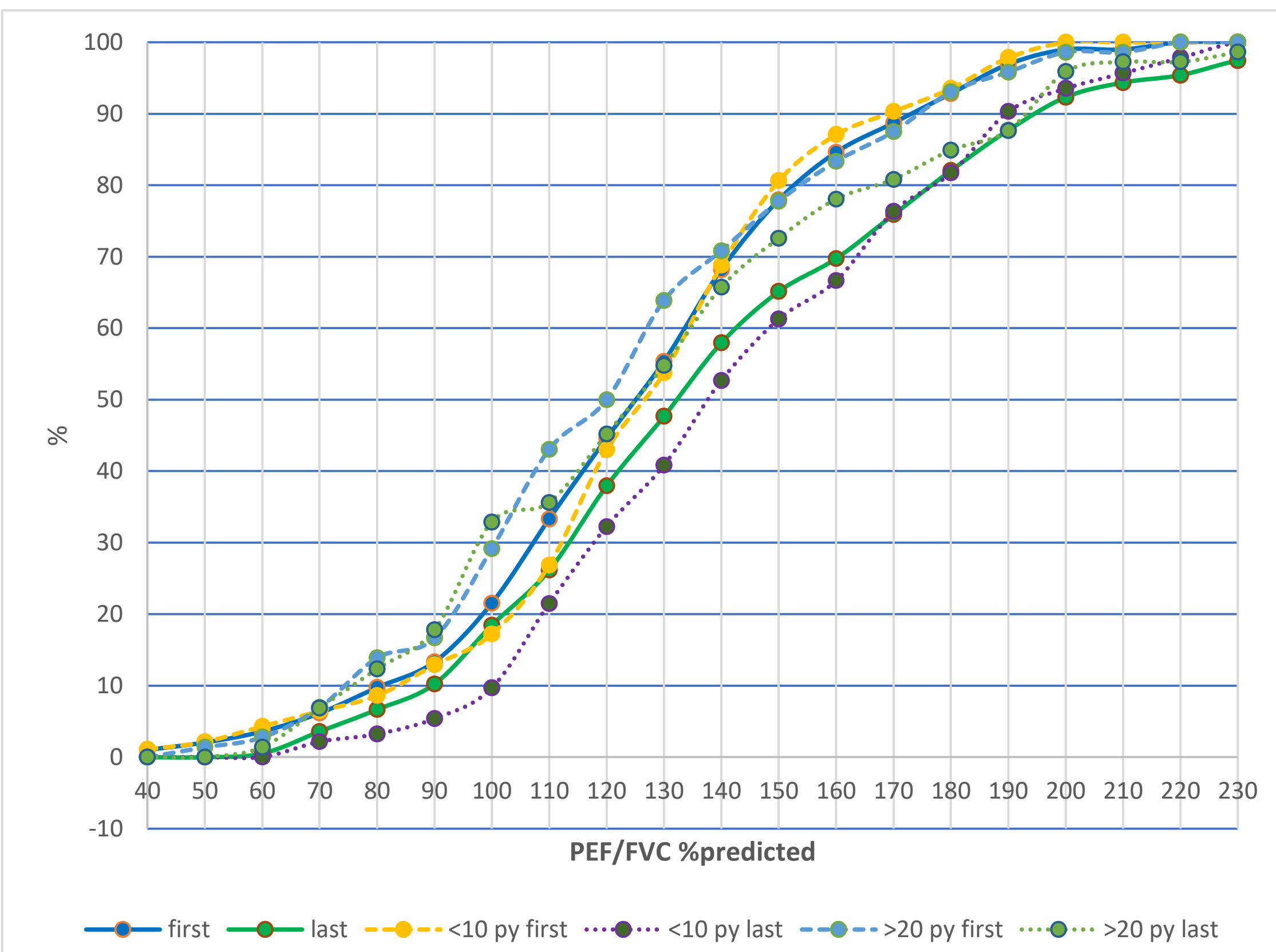
100
90
80
70
60
50
40
30
20
10
0
-10
%
40 50 60 70 80 90 100 110 120 130 140 150 160 170 180 190 200 210 220 230
PEF/FVC %predicted
first
last
<10 py first
<10 py last
>20 py first
>20 py last

Fig. contd.....

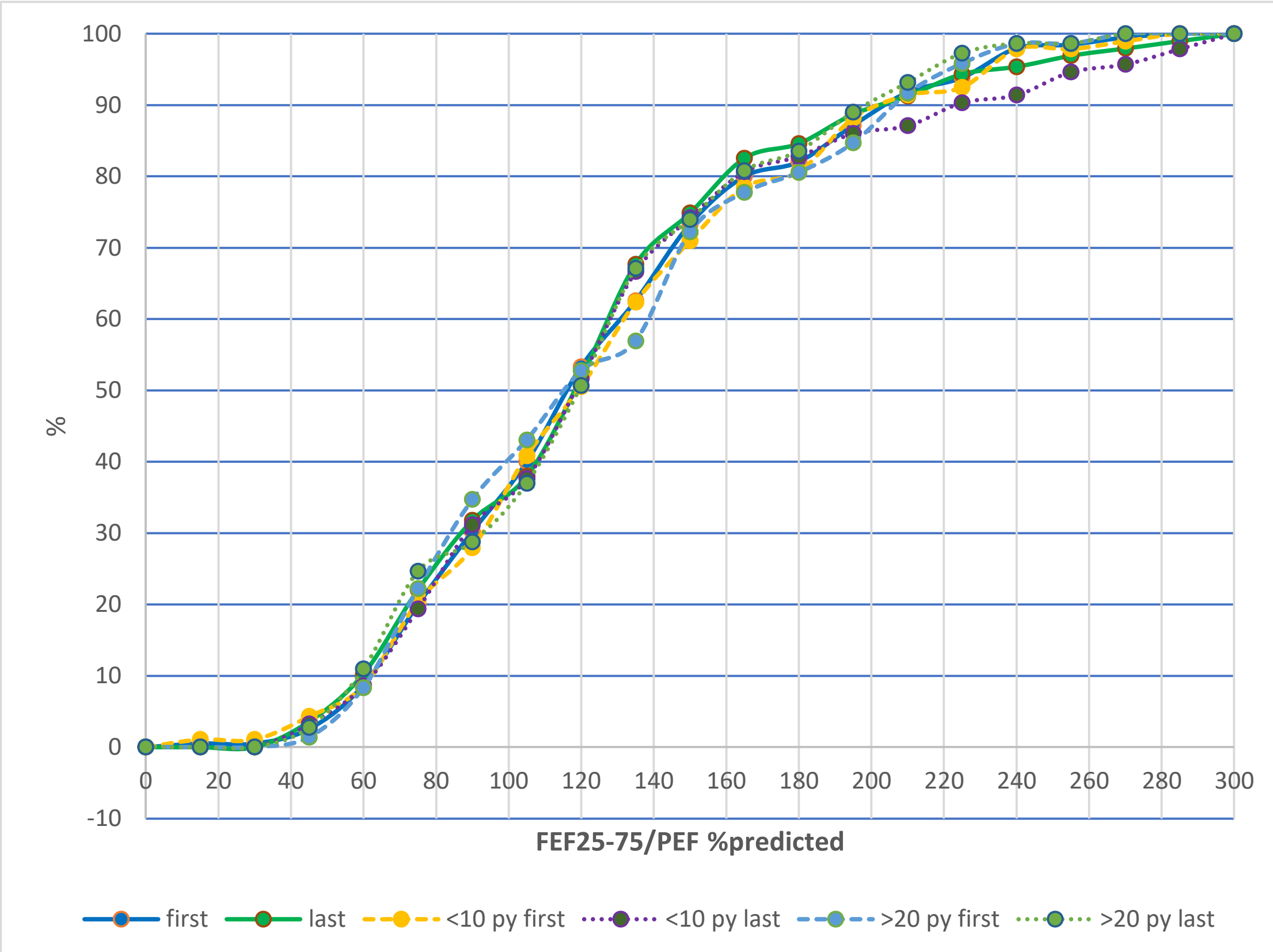
%
FEF25-75/PEF %predicted
first
last
<10 py first
<10 py last
>20 py first
>20 py last

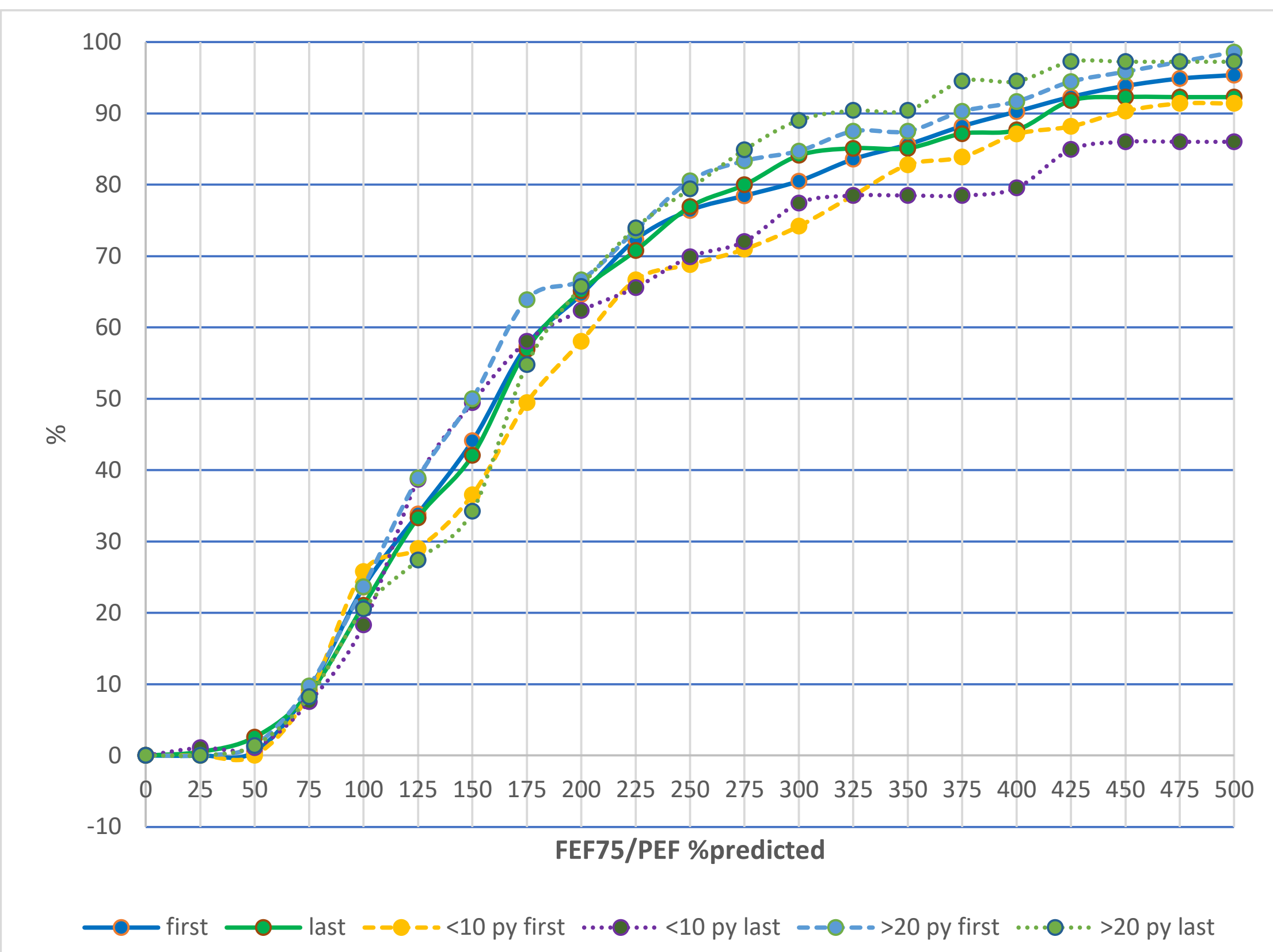
%
FEF75/PEF %predicted
first
last
<10 py first
<10 py last
>20 py first
>20 py last

Fig. contd.....

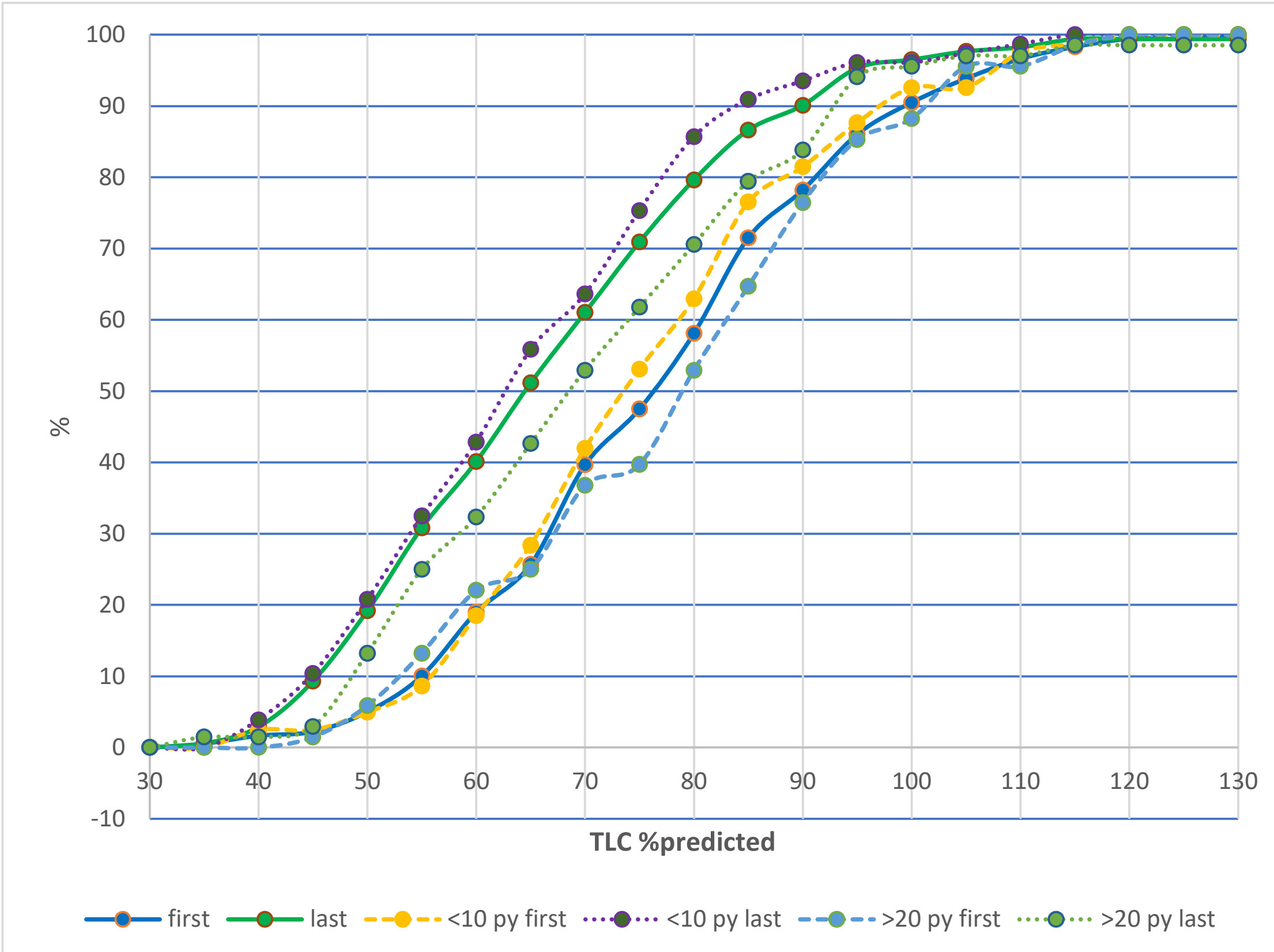
100
90
80
70
60
50
40
30
20
10
0
-10
%
30 40 50 60 70 80 90 100 110 120 130
TLC %predicted
first
last
<10 py first
<10 py last
>20 py first
>20 py last

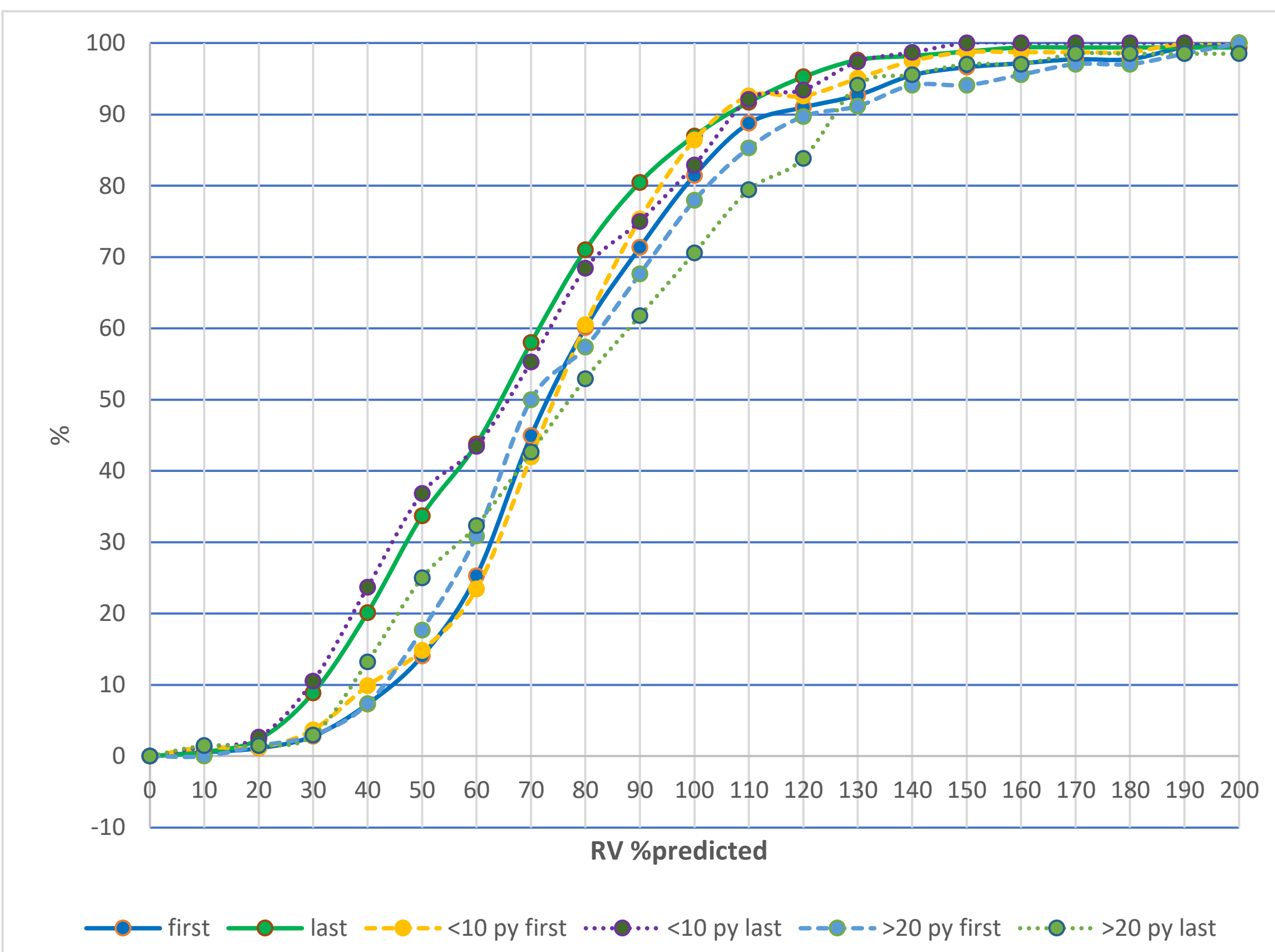
100
90
80
70
60
50
40
30
20
10
0
-10
%
0 10 20 30 40 50 60 70 80 90 100 110 120 130 140 150 160 170 180 190 200
RV %predicted
first
last
<10 py first
<10 py last
>20 py first
>20 py last

Fig. contd.....

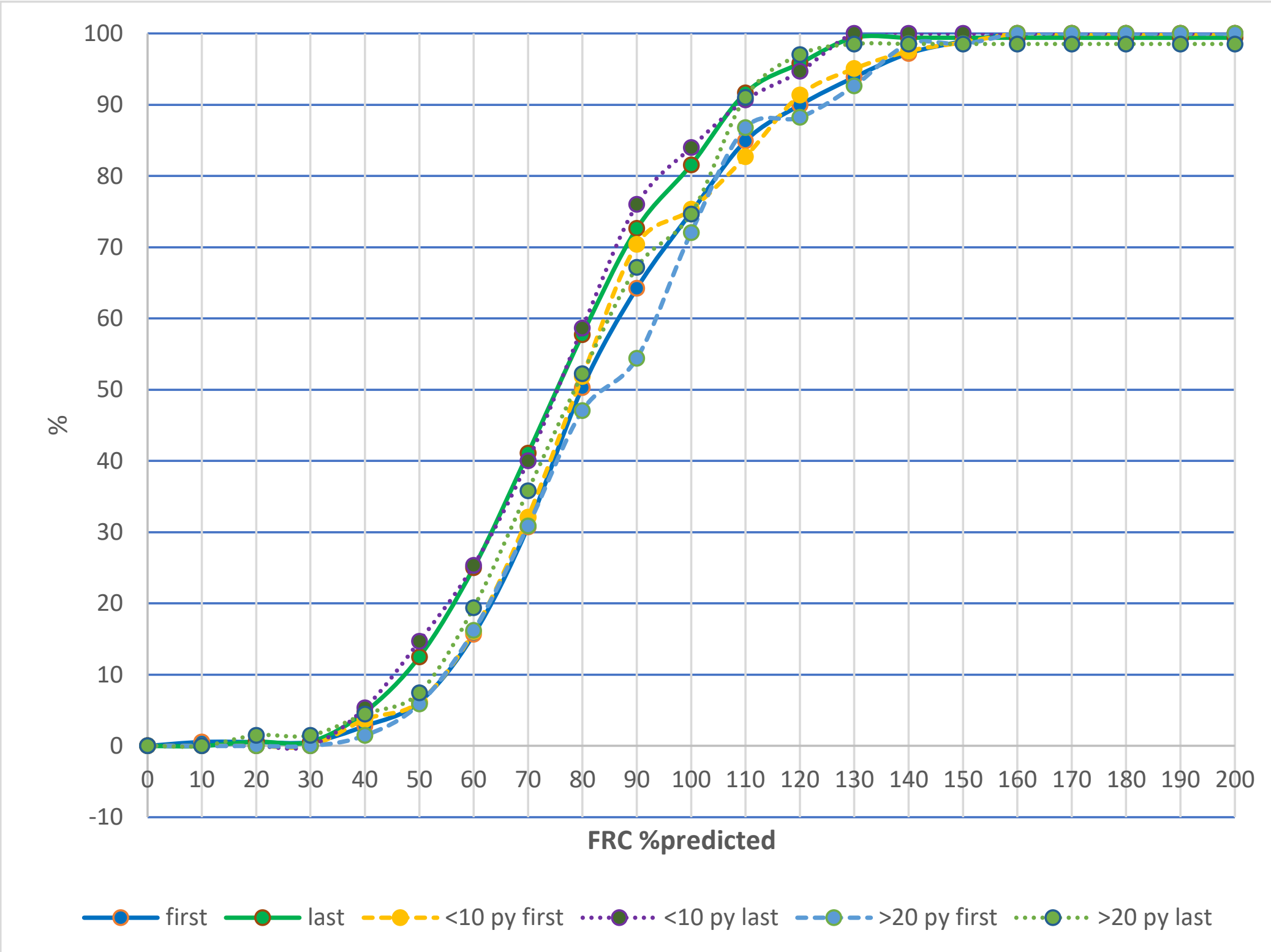
100
90
80
70
60
50
40
30
20
10
0
-10
%
0 10 20 30 40 50 60 70 80 90 100 110 120 130 140 150 160 170 180 190 200
FRC %predicted
first
last
<10 py first
<10 py last
>20 py first
>20 py last

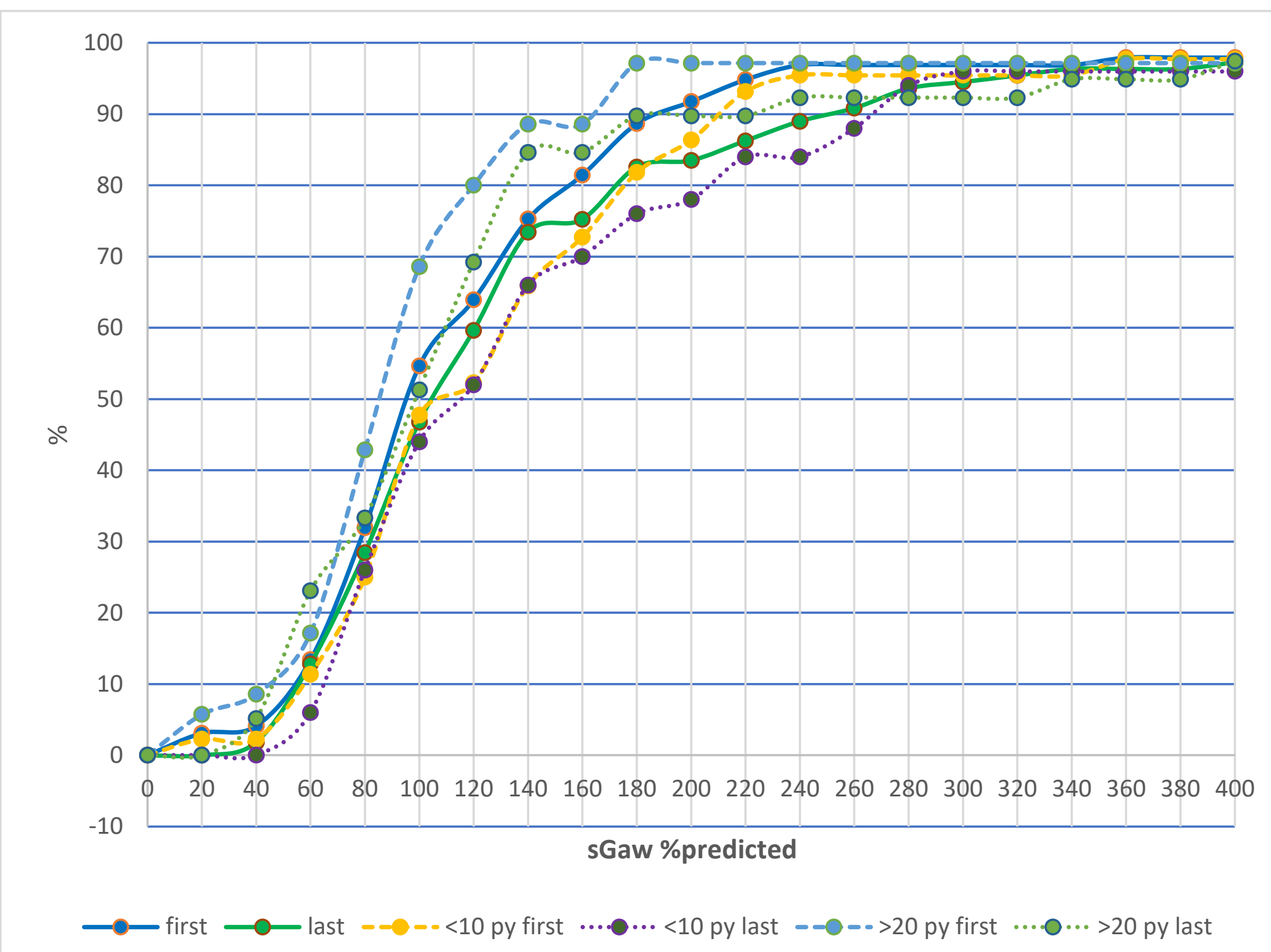
100
90
80
70
60
50
40
30
20
10
0
-10
%
0 20 40 60 80 100 120 140 160 180 200 220 240 260 280 300 320 340 360 380 400
sGaw %predicted
first
last
<10 py first
<10 py last
>20 py first
>20 py last

Fig. contd.....

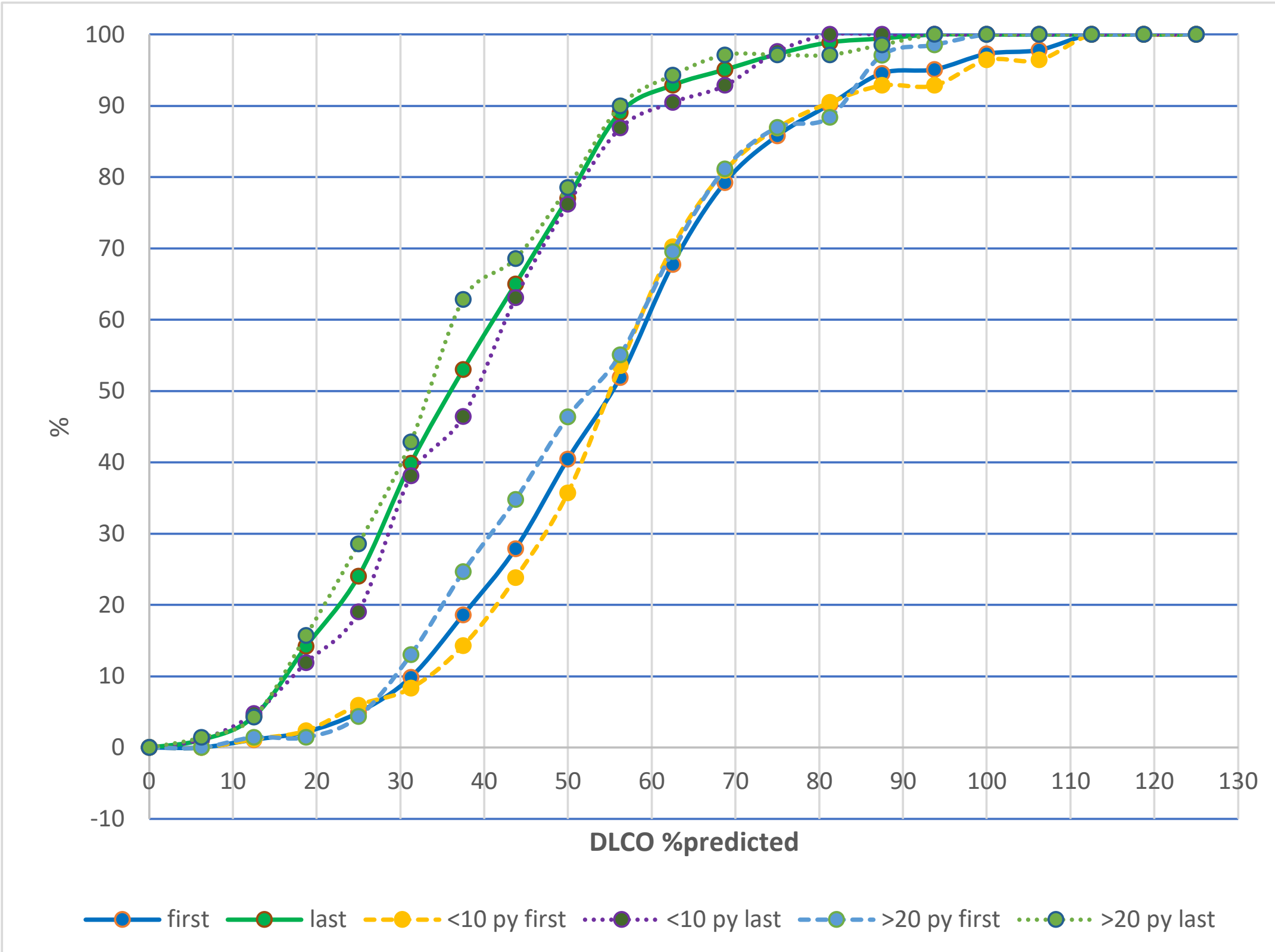
%
DLCO %predicted
first
last
<10 py first
<10 py last
>20 py first
>20 py last

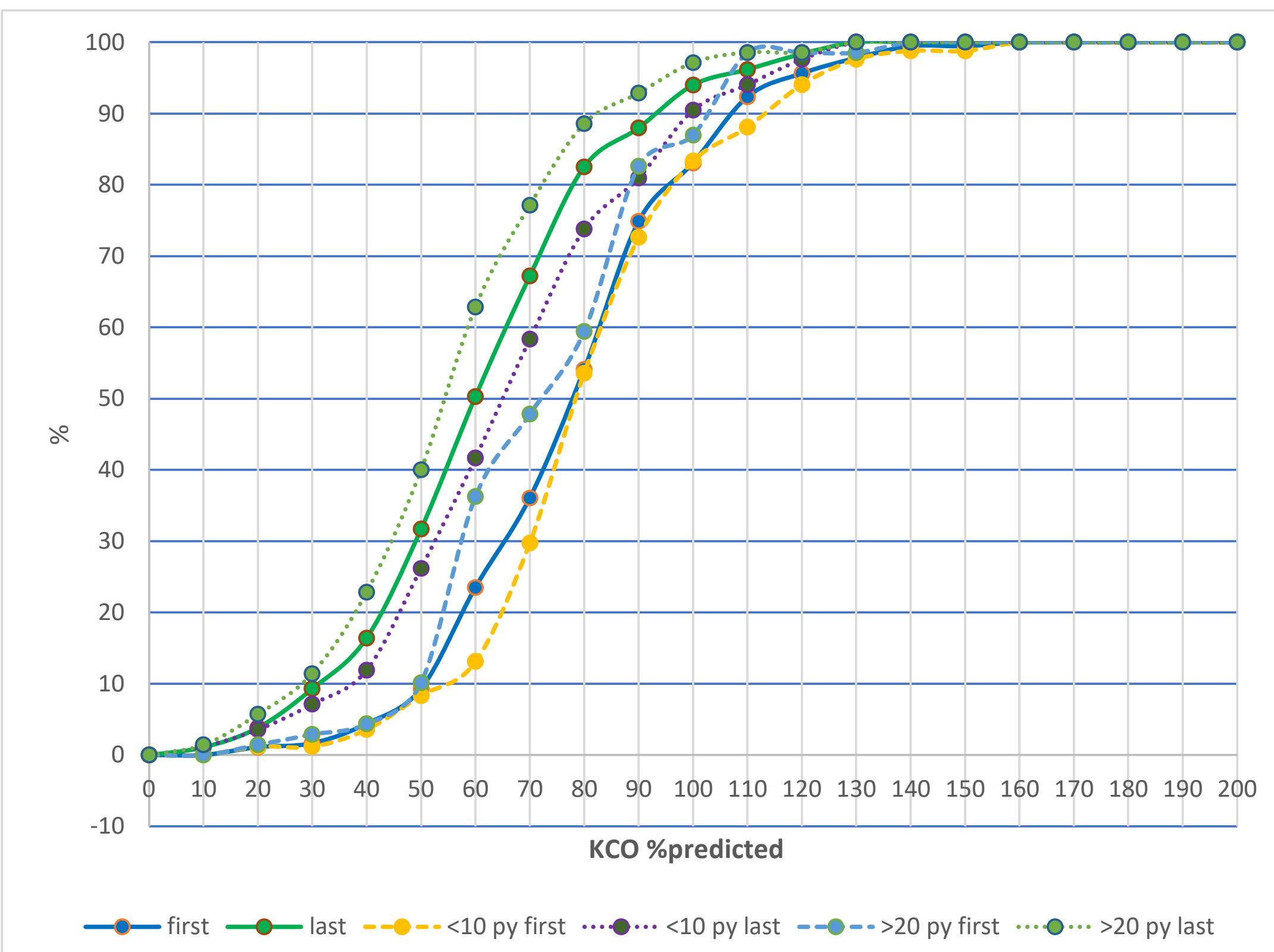
%
KCO %predicted
first
last
<10 py first
<10 py last
>20 py first
>20 py last

Fig. contd.....

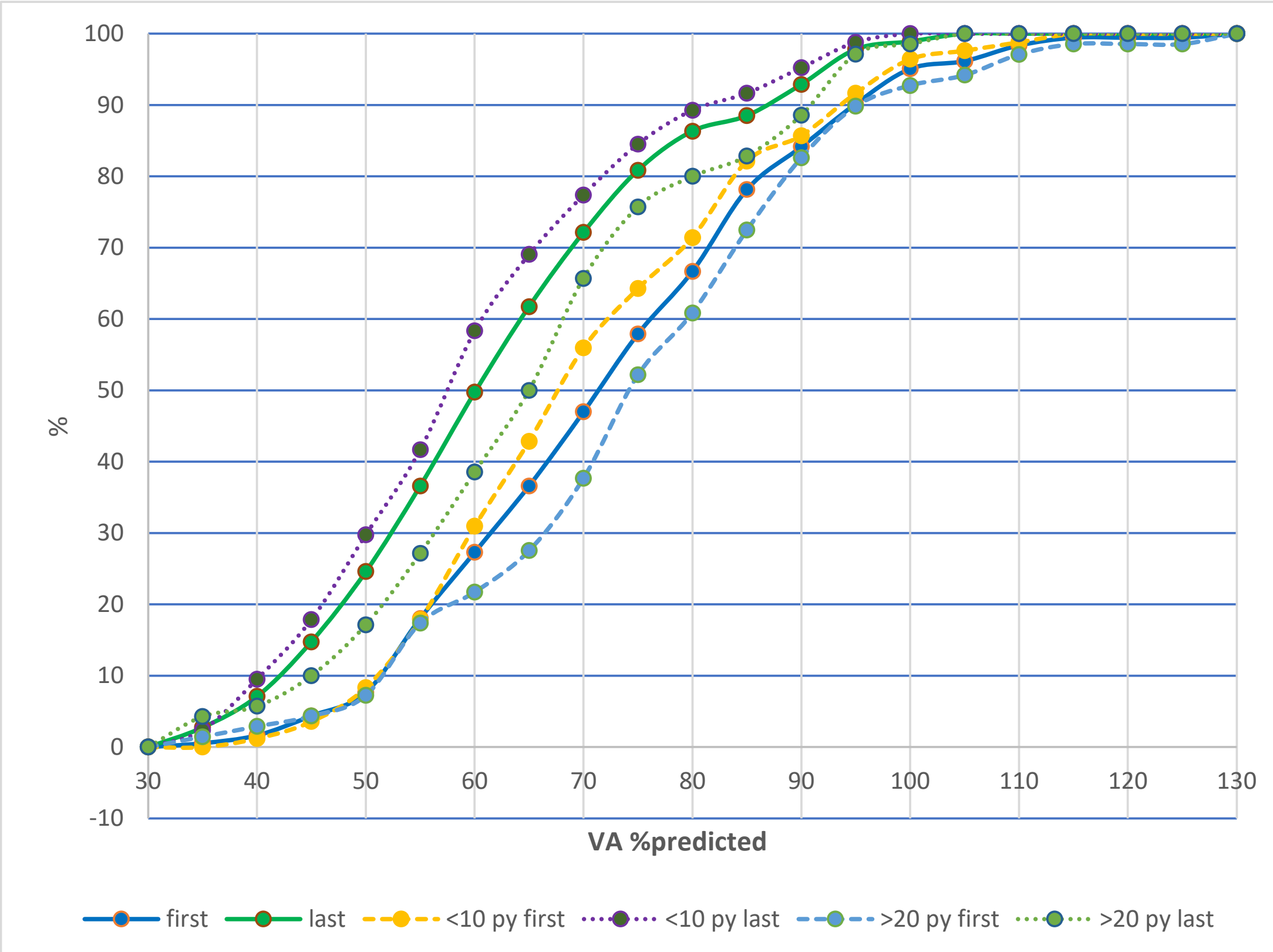
100
90
80
70
60
50
40
30
20
10
0
-10
%
30
40
50
60
70
80
90
100
110
120
130
VA %predicted
first
last
<10 py first
<10 py last
>20 py first
>20 py last

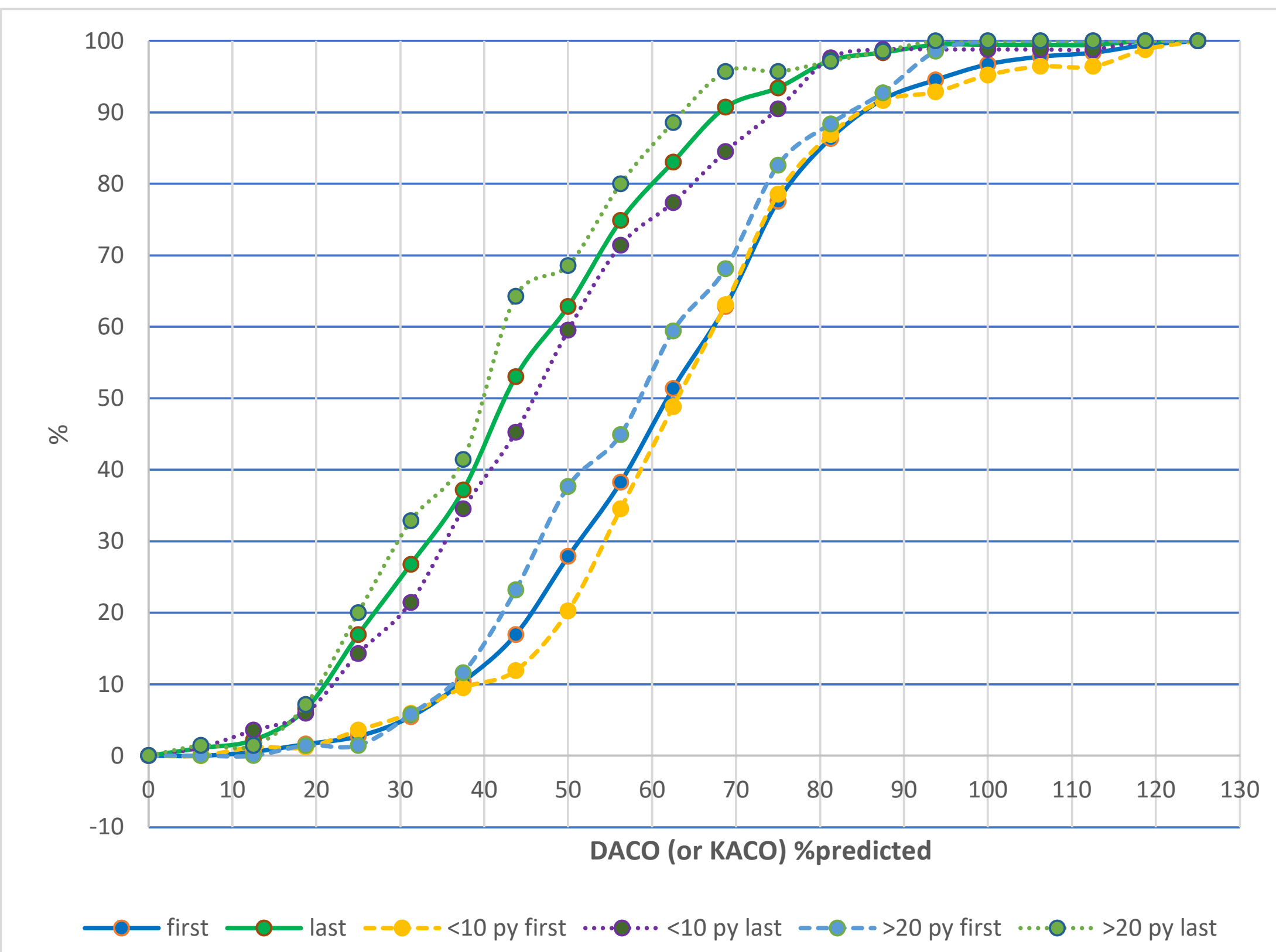
100
90
80
70
60
50
40
30
20
10
0
-10
%
0
10
20
30
40
50
60
70
80
90
100
110
120
130
DACO (or KACO) %predicted
first
last
<10 py first
<10 py last
>20 py first
>20 py last

Fig. contd.....

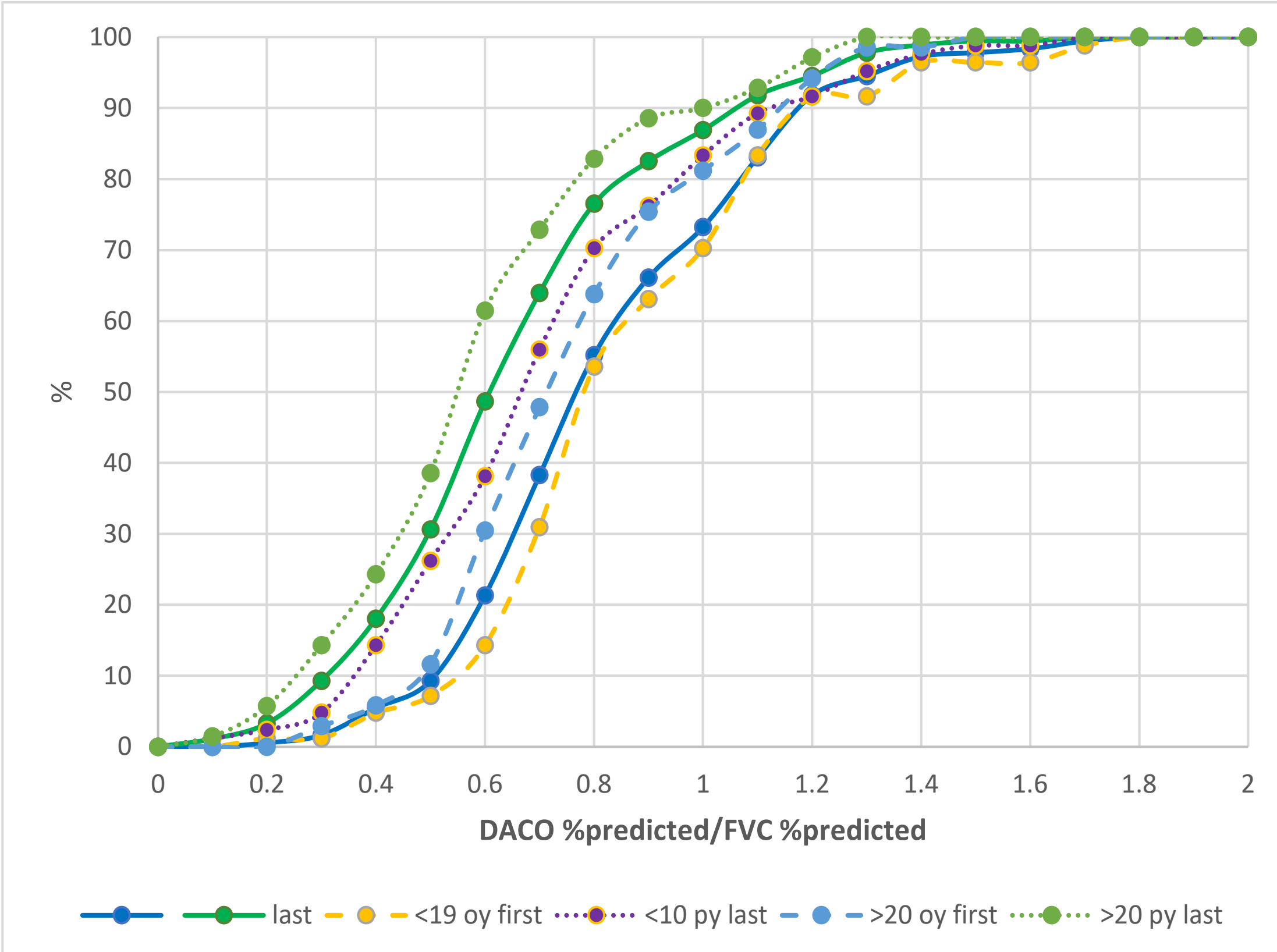
100
90
80
70
60
50
40
30
20
10
0
%
0
0.2
0.4
0.6
0.8
1
1.2
1.4
1.6
1.8
2
DACO %predicted/FVC %predicted
last
<19 oy first
<10 py last
>20 oy first
>20 py last

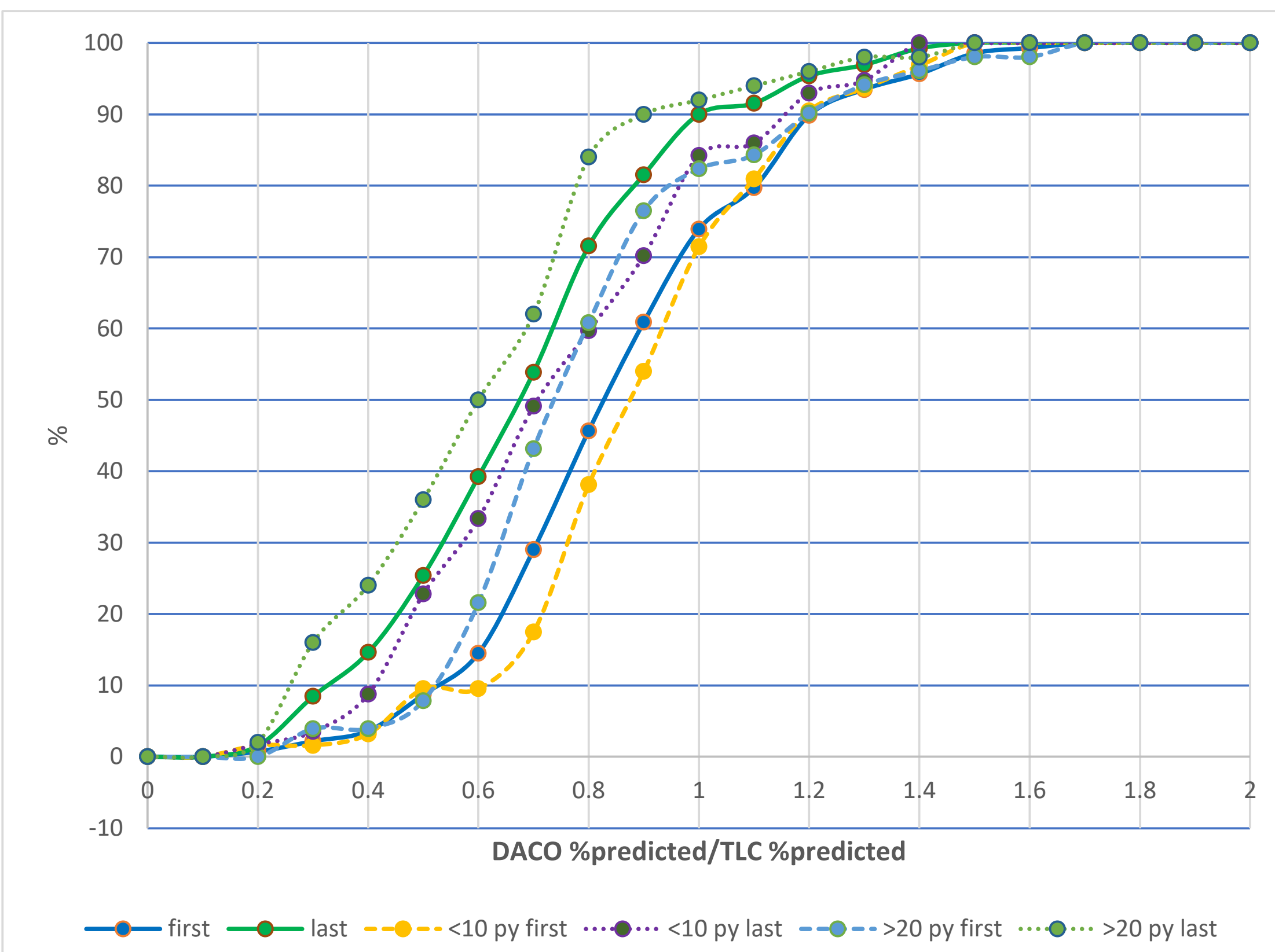
100
90
80
70
60
50
40
30
20
10
0
-10
%
0
0.2
0.4
0.6
0.8
1
1.2
1.4
1.6
1.8
2
DACO %predicted/TLC %predicted
first
last
<10 py first
<10 py last
>20 py first
>20 py last

Fig. contd.....

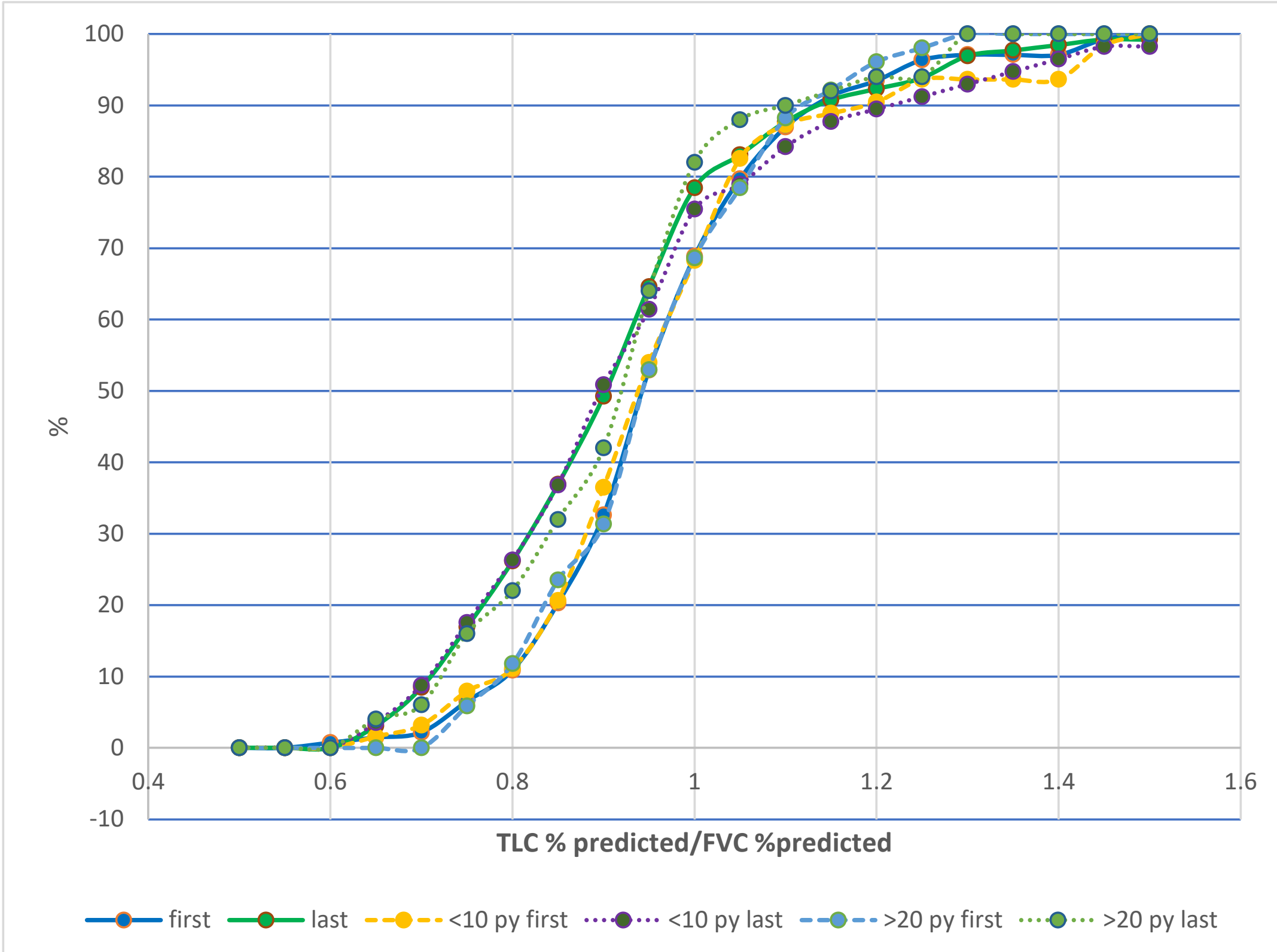
100
90
80
70
60
50
40
30
20
10
0
-10
%
0.4
0.6
0.8
1
1.2
1.4
1.6
TLC % predicted/FVC %predicted
first
last
<10 py first
<10 py last
>20 py first
>20 py last

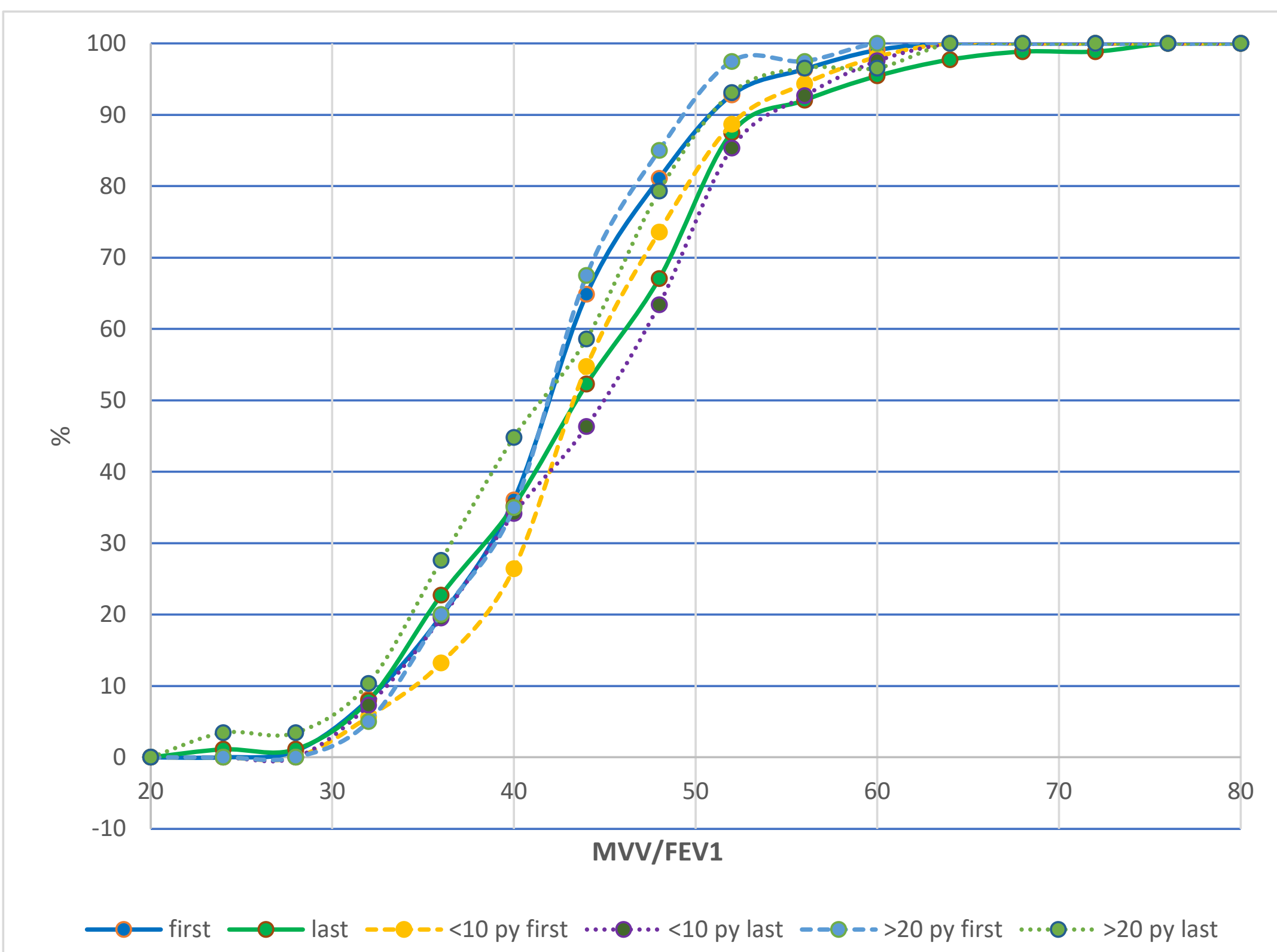
100
90
80
70
60
50
40
30
20
10
0
-10
%
20
30
40
50
60
70
80
MVV/FEV1
first
last
<10 py first
<10 py last
>20 py first
>20 py last

Fig. contd.....

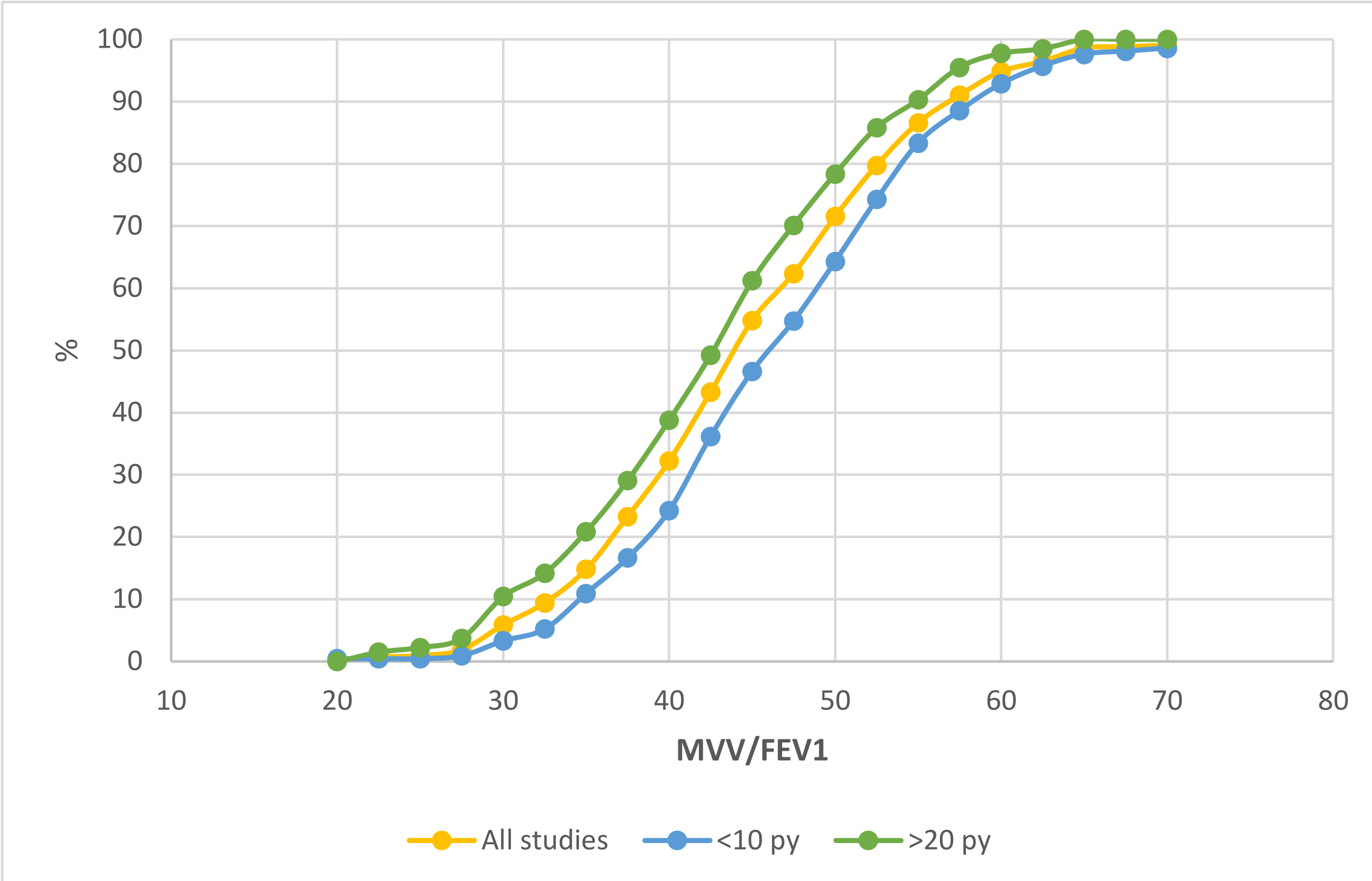
100
90
80
70
60
50
40
30
20
10
0
%
10
20
30
40
50
60
70
80
MVV/FEV1
All studies
<10 py
>20 py

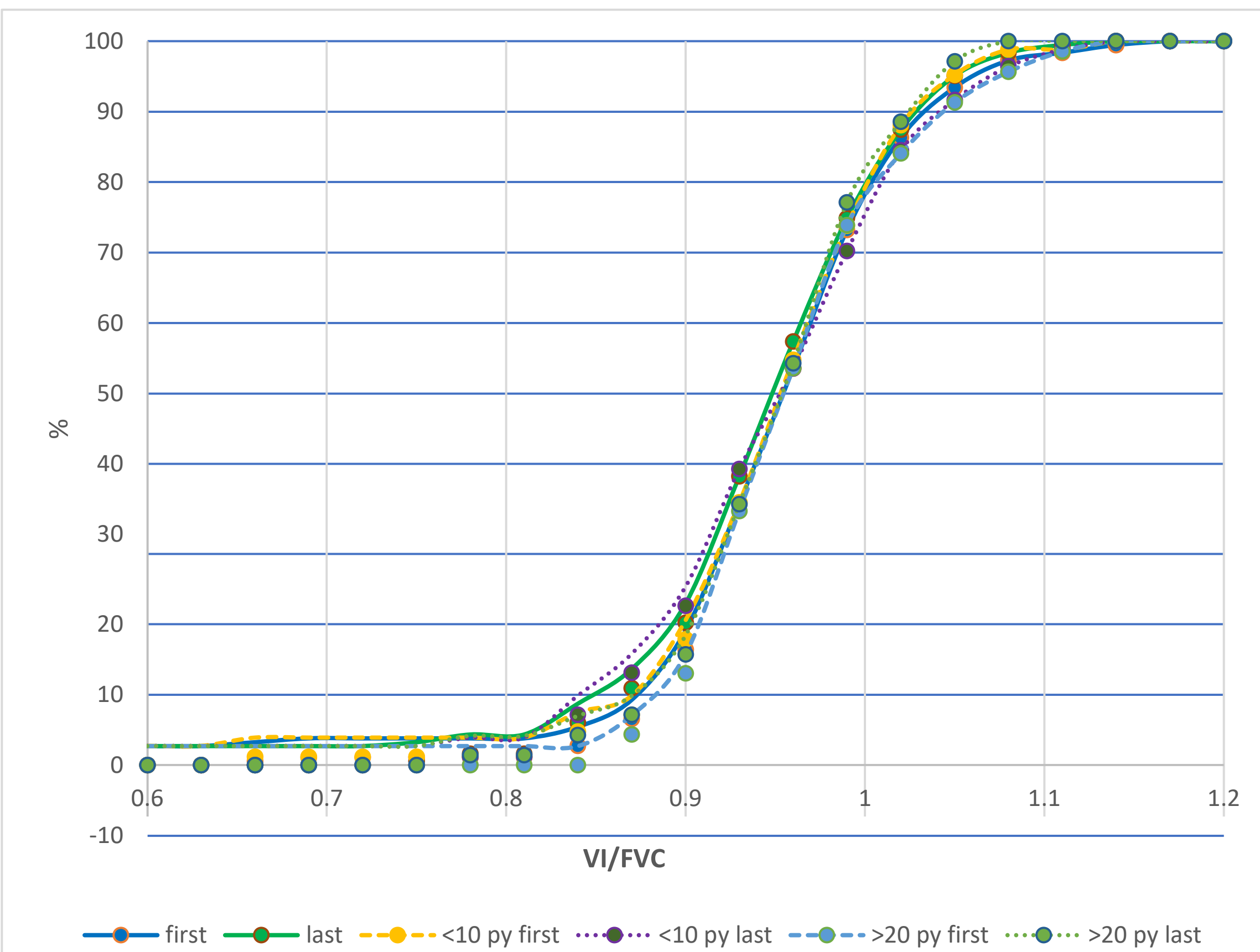
100
90
80
70
60
50
40
30
20
10
0
-10
%
0.6
0.7
0.8
0.9
1
1.1
1.2
VI/FVC
first
last
<10 py first
<10 py last
>20 py first
>20 py last

Fig. contd.....

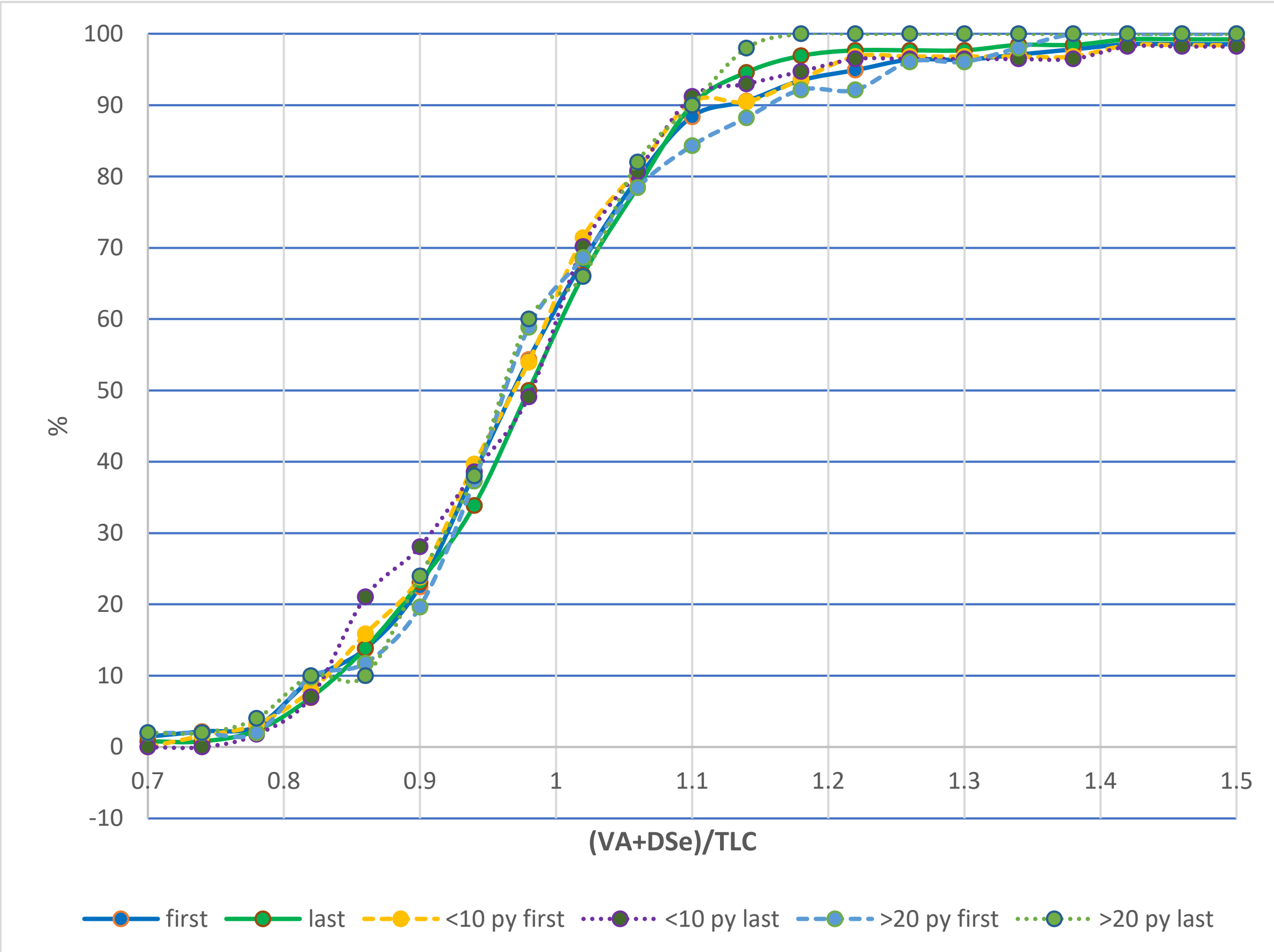

Supplementary Figures. Graphs of the cumulative % of studies having parameter values ≤ that on the x-axis for first and last studies by smoking status of % predicted parameters FEV05, FEV1, FEV3, FEV6, FEF25-75, FEF75, FVC, PEF, PEF/FEV1, FEV1/FEV3, FEV05/FVC, FEV1/FVC, FEV1/FVC ratio, FEV3/FVC, FEV6/FVC, FEF25-75/FVC, FEF75/FVC, PEF/FVC, FEF25-75/PEF, FEF75/PEF, TLC, RV, FRC, sGaw, DLCO, KCO, VA, DACO (or KACO); of DACO %predicted/FVC %predicted, DACO %predicted/TLC %predicted, TLC %predicted/FVC %predicted; and of MVV/FEV1, VI/FVC, and (VA+DSe)/TLC.
